# Mitochondrial transcription factor A in RORγt^+^ lymphocytes regulate small intestine homeostasis and metabolism

**DOI:** 10.1038/s41467-021-24755-9

**Published:** 2021-07-22

**Authors:** Zheng Fu, Joseph W. Dean, Lifeng Xiong, Michael W. Dougherty, Kristen N. Oliff, Zong-ming E. Chen, Christian Jobin, Timothy J. Garrett, Liang Zhou

**Affiliations:** 1 Department of Infectious Diseases and Immunology, College of Veterinary Medicine, University of Florida, Gainesville, FL USA; 2 Department of Medicine, University of Florida, Gainesville, FL USA; 3 Department of Laboratory Medicine and Pathology, Mayo Clinic, Rochester, MN USA; 4 Department of Pathology, Immunology and Laboratory Medicine, University of Florida, Gainesville, FL 32608 USA

**Keywords:** Adaptive immunity, Lymphocytes, Mucosal immunology, Crohn's disease

## Abstract

RORγt^+^ lymphocytes, including interleukin 17 (IL-17)-producing gamma delta T (γδT17) cells, T helper 17 (Th17) cells, and group 3 innate lymphoid cells (ILC3s), are important immune regulators. Compared to Th17 cells and ILC3s, γδT17 cell metabolism and its role in tissue homeostasis remains poorly understood. Here, we report that the tissue milieu shapes splenic and intestinal γδT17 cell gene signatures. Conditional deletion of mitochondrial transcription factor A (*Tfam*) in RORγt^+^ lymphocytes significantly affects systemic γδT17 cell maintenance and reduces ILC3s without affecting Th17 cells in the gut. In vivo deletion of *Tfam* in RORγt^+^ lymphocytes, especially in γδT17 cells, results in small intestine tissue remodeling and increases small intestine length by enhancing the type 2 immune responses in mice. Moreover, these mice show dysregulation of the small intestine transcriptome and metabolism with less body weight but enhanced anti-helminth immunity. IL-22, a cytokine produced by RORγt^+^ lymphocytes inhibits IL-13-induced tuft cell differentiation in vitro, and suppresses the tuft cell-type 2 immune circuit and small intestine lengthening in vivo, highlighting its key role in gut tissue remodeling.

## Abstract (web summary)

RORγt is known to play critical roles in the regulation of a number of immune cell subsets. Here the authors implicate mitochondrial transcription factor A in the regulation of intestinal RORγt^ +^ lymphocyte homeostasis and metabolic control in a murine in vivo model.

## Introduction

RORγt^+^ lymphocytes play an important role in immune homeostasis and disease pathogenesis, and include interleukin-17 (IL-17)-producing gamma delta T (γδT17) cells, T helper 17 (Th17) cells, and group 3 innate lymphoid cells (ILC3s). Recent studies showed that cell metabolism is critical for host immunity regulation^1–4^. Progress has been made toward better understanding the metabolic regulation of Th17 cells and ILC3s. For example, Th17 cells, compared to quiescent naive T cells, exhibit decreased oxidative phosphorylation and increased glucose metabolism-orchestrated anabolism^5^. In contrast, ILC3s have been shown to rely on an mTORC1-dependent metabolic program that integrates glycolysis and mitochondrial production of reactive oxygen species (ROS) for ILC3 proliferation and function^6^. γδT17 cells are a subset of innate-like T lymphocytes expressing the invariant gamma delta TCR (TCRγδ), and play a pivotal role in inflammation and autoimmunity as well as in cancer^7–14^. However, the current knowledge about the metabolic regulation in γδT cells, especially in γδT17 cells, is limited.

Human and mouse mitochondrial genomes contain protein-coding genes that are essential for the mitochondrial respiratory chain^15,16^. *Tfam* is a nuclear gene encoding transcription factor crucial for mitochondrial DNA replication and transcription^17–19^. Germline knockout of *Tfam* or conditional deletion of *Tfam* in hematopoietic stem cells (HSC) in mice leads to embryonic lethality^20,21^. Tfam deficiency severely affects mitochondrial respiration in T regulatory (T_reg_) cells and their maintenance and suppressive function^22^. Whether Tfam-mediated mitochondrial respiration plays a role in γδT cell maintenance or function remains unknown.

Environmental stimuli play an important role in shaping local tissue immunity^23–28^. Transforming growth factor-β (TGF-β), which promotes Foxp3 expression, is enriched in the gut to favor intestinal T_reg_ differentiation^23^. Furthermore, bile acid metabolites have been shown to control T helper (Th)17 and T_reg_ cell differentiation and also modulate RORγt^+^ T_reg_ cell homeostasis in the gut^24,25^. An emerging concept of transcriptional imprinting on tissue-specific immune cells to allow them to adapt or respond to certain environmental stimuli has recently also been appreciated^29,30^. For example, the aryl hydrocarbon receptor (Ahr), a ligand-dependent transcription factor and environmental sensor that can be activated by food and microbiome-derived metabolites in the gut^29,31^, is highly expressed by intestinal T_reg_ cells and innate lymphoid cells (ILCs) compared with their counterparts in other tissues^32–34^. Whether and how tissue milieu shapes γδT17 cell gene signature have yet to be described.

The small intestine is the major organ for nutrient absorption, and also provides important immunity against mucosal pathogens. However, dysregulation of immune responses can cause inflammation and tissue damage of the small intestine (e.g., Crohn’s disease). Modulation of the crosstalk between gut epithelial cells and immune cells are important for gut tissue homeostasis and disease pathogenesis. Tuft cells, a subset of specialized chemosensory epithelial cells, secrete IL-25 for ILC2 activation to control parasitic infection of helminths and protists^35–38^. Reciprocally, ILC2s can secrete IL-13 to promote intestinal tuft cell differentiation and proliferation. Enhanced ILC2 function has been shown to increase the number of small intestinal secretory epithelial cells including tuft cells, resulting in tissue remodeling and lengthening^38^. However, host cellular mechanism responsible for regulation of the tuft cell–ILC2 circuit in mice is unclear.

Here we show that γδT17 cells were highly dependent on mitochondrial metabolism for their maintenance. Intestinal γδT17 cells had a unique transcriptional program with an adaptation to environmental stimuli by engaging the Ahr pathway and TGF-β pathway. Genetic ablation of *Tfam* in RORγt^+^ lymphocytes led to a systemic decrease of γδT17 cells and a reduction of ILC3s but not Th17 cells in the gut, concomitant with the substantial increase of intestinal type 2 immune responses (ILC2s and Th2 cells), and small intestine tissue remodeling and inflammation in mice. Our data further indicated a key role for RORγt^+^ lymphocyte effector cytokine IL-22 in regulation of the tuft cell-type 2 immune circuit and small intestine lengthening.

## Results

### Tfam deficiency affects γδT17 cell maintenance

**Fig. 1:**
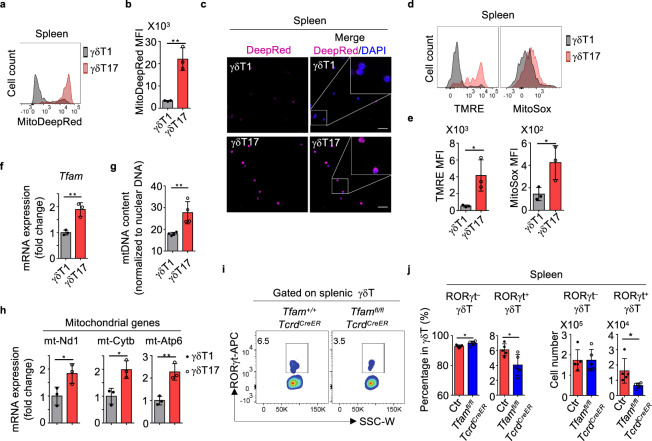
Tfam deficiency affects γδT1 and γδT17 cells differentially. **a** MitoTracker-Deep Red staining in splenic γδT1 and γδT17 cells by flow cytometry. Representative data of three independent experiments. **b** MitoTracker-Deep Red mean fluorescence intensity (MFI) in **a** (*n* = 3 for each group) (**P = 0.0032). Compiled data from one experiment. **c** Confocal analysis of MitoTracker-Deep Red staining in splenic γδT1 and γδT17 cells. Scale bar, 50 µm. Representative data of two independent experiments. **d** Tetramethylrhodamine, ethyl ester (TMRE) and MitoSox^TM^ Red staining in splenic γδT1 and γδT17 cells by flow cytometry. Representative data of three independent experiments. **e** TMRE (*P = 0.0281) and MitoSox^TM^ MFI (*P = 0.0404) in **d** (*n* = 3 for each group). Compiled data from one experiment. **f** Expression of *Tfam* measured by quantitative real-time PCR (qRT-PCR) in the splenic γδT1 and γδT17 cells (*n* = 3 for each group) (**P = 0.0053). Each dot indicated a biological repeat of individual RNA sample pooled from two mice. Compiled data from two independent experiments. **g** Mitochondrial DNA content measured by qPCR (mt-Nd1 compared to nuclear gene *Hbb*) in splenic γδT1 and γδT17 cells (*n* = 4 for each group) (**P = 0.0091). Compiled data from two independent experiments. **h** Expression of mitochondrial gene mt-Nd1 (*P = 0.0427), mt-Cytb (*P = 0.0177), and mt-Atp6 (**P = 0.0062) by qRT-PCR in splenic γδT1 and γδT17 cells (*n* = 3 for each group). Each dot indicated a biological repeat of individual RNA sample pooled from two mice. Compiled data from two independent experiments. **i** RORγt expression by splenic γδT cells by flow cytometry in mice with indicated genotypes. Representative data of three independent experiments. Mice were treated with tamoxifen daily for 5 days. Data were collected 3 weeks after the last tamoxifen injection. **j** RORγt^–^ γδT and RORγt^+^γδT (i.e., γδT17) cell percentages (RORγt^–^ γδT, *P = 0.0299; RORγt^+^ γδT, *P = 0.0218) within total splenic γδT cells as shown in **i** and absolute cell numbers (RORγt^–^ γδT, *P* = 0.9856; RORγt^+^ γδT: *P = 0.0311) (*n* = 5 for each group). Compiled data from two independent experiments. Ctr included *Tfam*^*+/+*^*Tcrd*^*CreER*^ and *Tfam*^*fl/+*^*Tcrd*^*CreER*^. Data are shown as mean ± SD in **b**, **e**–**h**, **j**.

Compared to Th17 cells and ILC3s, little is known about metabolic regulation of γδT17 cells. Two populations of γδT cells could be defined based on CD44 and CD45RB surface expression in the spleen^39–41^. CD44^medium^CD45RB^high^ γδT cells, AKA γδT1 cells, expressed T-bet and produced IFN-γ. In contrast, CD44^high^CD45RB^–^ γδT cells, AKA γδT17 cells, expressed RORγt and produced IL-17 (Supplementary Fig. 1a, b). In order to characterize the mitochondrial metabolisms of these two subsets of γδT cells, we first determined the mitochondrial content and activity. Compared with γδT1 cells, splenic γδT17 cells contained more mitochondrial mass and/or potential as revealed by mitochondrial staining of MitoTracker-Deep Red (Fig. 1a–c). Higher mitochondrial membrane potential in γδT17 cells was further indicated by tetramethylrhodamine ethyl ester (TMRE) staining (Fig. 1d, e). Furthermore, γδT17 cells generated more mitochondrial-specific reactive oxygen species (ROS), an indicator of mitochondrial activity as revealed by MitoSOX™ Red staining (Fig. 1d, e). *Tfam* expression, which is critical for regulating mitochondrial gene replication and transcription^20^, was higher in γδT17 cells than in γδT1 cells (Fig. 1f). Accordingly, elevated mitochondrial DNA content, mitochondrial gene expression (e.g., mt-Nd1, mt-Cytb, mt-Atp6), and cellular ATP production were observed in γδT17 cells than in γδT1 cells (Fig. 1g, h and Supplementary Fig. 1c). However, uptake of 2-(*N*-(7-nitrobenz-2-oxa-1,3-diazol-4-yl) amino)-2-deoxyglucose (2-NBDG), a fluorescent glucose analog for monitoring glucose uptake, did not show significant difference between γδT17 and γδT1 cells (Supplementary Fig. 1d, e). In addition, expression of several key genes involved in glycolysis (e.g., *Slc2a1*, *Slc2a3*, *Hk1*, *Pkm*, *Pdk1*, and *Ldha*) did not show a significant difference between γδT17 cells and γδT1 cells (Supplementary Fig. 1f). Together, these data suggest that γδT17 cells exhibit higher mitochondrial mass and activity compared with γδT1 cells.

To further understand the role of mitochondrial metabolism in γδT cells, we crossed the mice carrying loxP-flanked *Tfam* alleles (*Tfam*^*fl/fl*^) with *Tcrd*^*CreER*^ mice in which Cre recombinase nuclear localization and subsequently gene deletion in γδT cells are induced by tamoxifen treatment^42^ to specifically delete *Tfam* in γδT cells (hereafter as *Tfam*^*fl/fl*^*Tcrd*^*CreER*^ mice). Results showed that deletion of the floxed *Tfam* locus in both γδT1 and γδT17 cells, but not in non-γδT lymphocytes in *Tfam*^*fl/fl*^*Tcrd*^*CreER*^ mice upon tamoxifen treatment (Supplementary Fig. 1g), resulting in decreased mitochondrial mass and activity in both γδT1 and γδT17 cells of *Tfam*^*fl/fl*^*Tcrd*^*CreER*^ mice compared with their littermate controls (Supplementary Fig. 1h, i). Notably, while splenic RORγt^–^ γδT cells, which include γδT1 cells, did not show a significant reduction, RORγt^+^ γδT (i.e., γδT17) cell percentages and numbers were significantly decreased in *Tfam*^*fl/fl*^*Tcrd*^*CreER*^ mice compared to their littermate controls upon tamoxifen treatment (Fig. 1i, j). Together, these results suggested that Tfam is important in regulating γδT17 but not γδT1 cell maintenance, consistent with the higher mitochondrial content and activity of γδT17 cells compared with γδT1 cells.

### Tfam is critical for ILC3 and γδT17 cell maintenance in the gut

Under steady-state condition, γδT17 cells, together with ILC3s and Th17 cells that express RORγt, are predominantly present in the mucosal tissues such as the intestine^34,43^. Unlike RORγt^–^ γδT cells predominantly in the intraepithelial lymphocyte (IEL) compartment, γδT17 cells were found in the lamina propria lymphocytes (LPL) of the gut (Supplementary Fig. 2 for gating strategy and Supplementary Fig. 3a). Because of relatively inefficient deletion of Tfam in γδT17 cells of *Tfam*^*fl/fl*^*Tcrd*^*CreER*^ mice (Supplementary Fig. 1g) and to further determine the role of *Tfam* in γδT17 cells, we crossed *Tfam*^*fl/fl*^ mice with *Rorc-cre* mice (hereafter as *Tfam*^*fl/fl*^*Rorc-cre* mice) in which Tfam was deleted in RORγt^+^ lymphocytes. Results showed an efficient deletion of the floxed *Tfam* locus in RORγt^+^ lymphocytes, including γδT17 cells, ILC3s, and Th17 cells (Supplementary Fig. 3b). There were no significant changes in CD4, CD8 single (SP) or double positive (DP) cells in the thymi of *Tfam*^*fl/fl*^*Rorc-cre* mice compared to littermate controls (Supplementary Fig. 3c, d). In addition, CD4^+^ or CD8^+^ T cells were not significantly changed in the spleens of *Tfam*^*fl/fl*^*Rorc-cre* mice (Supplementary Fig. 3e, f), suggesting that Tfam deficiency did not affect CD4^+^ or CD8^+^ T cell development.

**Fig. 2:**
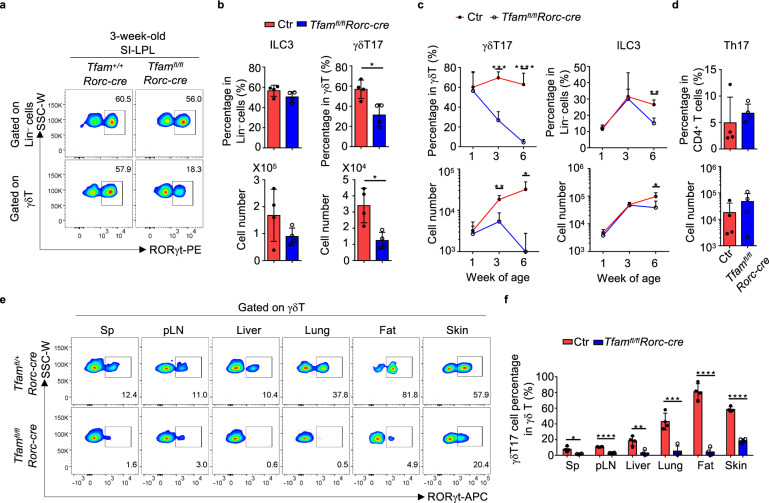
Tfam is critical for γδT17 cell and ILC3 maintenance. **a** Flow cytometry analyses of ILC3s (upper panel, gated on lineage-negative lymphocytes) and γδT17 cells (lower panel, gated on γδT cells) from small intestine lamina propria (LPL) of 3-week-old mice of indicated genotypes. Representative data of three independent experiments. Lineage markers in **b** and **c** include CD3, CD5, CD19, Ly6G, CD11b, and CD11c. **b** γδT17 cell percentages in total γδT cells (percentage, *P = 0.0120; cell number, *P = 0.0106), and ILC3 percentages in lineage-negative (Lin^–^) cells (upper panel) (*P* = 0.1575) and cell numbers (lower panel) (*P* = 0.1725) from the small intestine of mice shown in **a** (*n* = 4 for each group). Compiled data from two independent experiments. **c** γδT17 cell percentages in total γδT cells and ILC3 percentage in lineage-negative cells (upper panel) (γδT17: week 3, ***P = 0.0002, week 6, ****P < 0.0001; ILC3: week 6, **P = 0.0019) and cell numbers (lower panel) (γδT17: week 3, **P = 0.0035, week 6, *P = 0.0101; ILC3: week 6, *P = 0.0396) of the large intestine of mice at indicated ages (*n* = 4 for each group). The rest time points showed no significance between Ctr and *Tfam*^*fl/fl*^*Rorc-cre* (*P* > 0.05). Compiled data from two independent experiments. **d** Th17 cell percentages in total CD4^+ ^T cells (upper panel) (*P* = 0.1705) and cell numbers (lower panel) (*P* = 0.0950) of the large intestine of 6-week-old age mice (*n* = 4 for each group). Compiled data from two independent experiments. **e** RORγt expression in γδT cells of various organs (Sp spleen, pLN peripheral lymph nodes) of mice with indicated genotypes measured by flow cytometry. Representative data of three independent experiments. **f** γδT17 cell percentages in total γδT cells from the various organs as shown in **e** (*n* = 4 for each group). Sp, *P = 0.0121; pLN, ****P < 0.0001; liver, **P = 0.0043; lung, ***P = 0.0009; fat, ****P < 0.0001; skin, ****P < 0.0001. Compiled data from two independent experiments. Ctr included *Tfam*^*+/+*^*, Tfam*^*fl/+*^*, Tfam*^*fl/fl*^*, Tfam*^*+/+*^*Rorc-cre*, and *Tfam*^*fl/+*^*Rorc-cre* mice. Data are shown as mean ± SD in **b**–**d**, **f**.

Intriguingly, at week 3 of age, we observed a reduction of RORγt^+^ lymphocytes, especially γδT17 cells in the small intestine of *Tfam*^*fl/fl*^*Rorc-cre* mice (Fig. 2a, b). The cellular ATP production was also decreased in Tfam-deficient γδT17 cells compared with control γδT17 cells (Supplementary Fig. 3g). We noticed inconsistent success rate of small intestinal lymphocyte preparation in older mice, presumably due to tissue and cellular changes in the gut (see below). Thus, we examined dynamic changes of lymphocytes in the large intestine of mice at different ages. In 1-week-old *Tfam*^*fl/fl*^*Rorc-cre* mice, both γδT17 cells and ILC3s did not show a significant change compared to their littermate control mice, suggesting that Tfam deletion does not affect the early development of γδT17 cells and ILC3s (Fig. 2c and Supplementary Fig. 3h). In 3-week-old *Tfam*^*fl/fl*^*Rorc-cre* mice, γδT17 cells were significantly decreased, while ILC3s did not show an obvious change compared to their littermate control mice (Fig. 2c and Supplementary Fig. 3i). By 6 weeks of age, γδT17 cells were markedly reduced, and a substantial reduction of ILC3s was also apparent (Fig. 2c and Supplementary Fig. 3j). In contrast, Th17 cells had no significant change in *Tfam*^*fl/fl*^*Rorc-cre* mice at 6 weeks of age compared to littermate control mice (Fig. 2d and Supplementary Fig. 3j). It has been reported that the T cell receptor (TCR) chain Vγ2 or Vγ4 represented the majority of γδT17 cells in different tissues^11,44–46^. Consistently, the majority of γδT17 cells in the gut LPL expressed the TCR chain Vγ2 or Vγ4, and both RORγt^+^Vγ2^+^ and RORγt^+^Vγ4^+^ were markedly reduced in 6-week-old *Tfam*^*fl/fl*^*Rorc-cre* mice (Supplementary Fig. 3k, l). Of note, γδT17 cells also showed a substantial systemic decrease in various lymphoid and non-lymphoid tissues of the mice (i.e., spleen (Sp), peripheral lymph nodes (pLN), liver, lung, fat, and skin) of *Tfam*^*fl/fl*^*Rorc-cre* mice at 6 weeks of age (Fig. 2e, f), suggesting a crucial requirement for Tfam in systemic γδT17 cell maintenance. On a per cell basis, similar amounts of IL-17 and IL-22 were made by Tfam-deficient γδT17 cells and ILC3s compared to Tfam-sufficient cells, suggesting that Tfam does not regulate the effector cytokine production of γδT17 cells and ILC3s (Supplementary Fig. 3m–p). Together, these data indicate that Tfam deficiency in RORγt^+^ lymphocytes selectively affect the maintenance of γδT17 cells and ILC3s but not Th17 cells.

### *Tfam* deficiency in γδT17 cells perturb the small intestine homeostasis

**Fig. 3:**
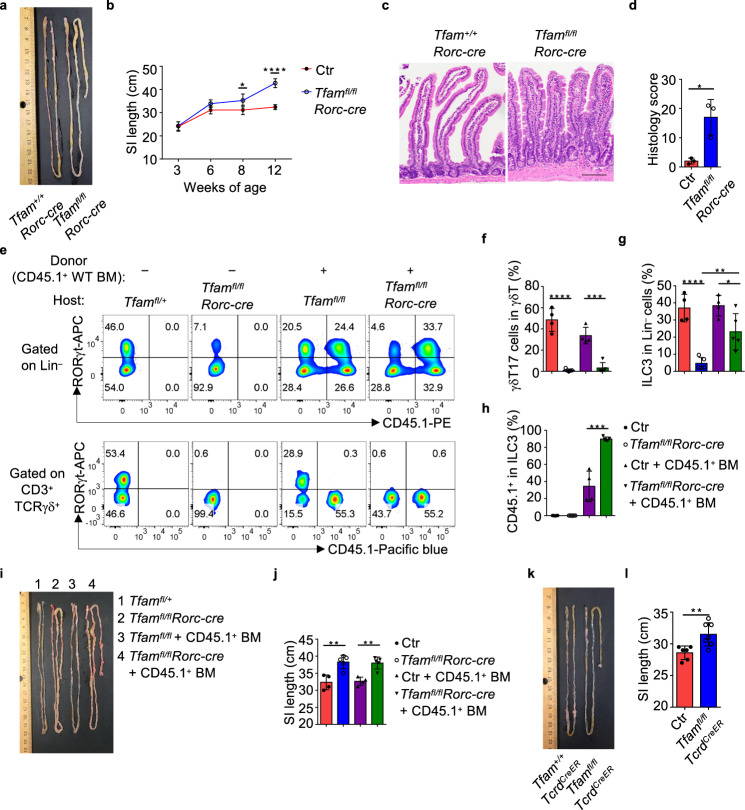
Tfam deficiency in γδT17 cells cause small intestine lengthening. **a** Picture of the small intestine of 8-week-old littermate *Tfam*^+/+^ *Rorc-cre* and *Tfam*^*fl/fl*^*Rorc-cre* mice. Representative data of three independent experiments. **b** Small intestine length of mice with indicated genotypes at different ages (week 3, *P* = 0.8542; week 6, *P* = 0.056; week 8, *P = 0.03853; week 12, ****P < 0.0001). Ctr, *n* = 5 for 3-week-old and 12-week-old age, *n* = 4 for 6-week-old age, *n* = 3 for 8-week-old age, *Tfam*^*fl/fl*^*Rorc-cre*, *n* = 4. Compiled data from three independent experiments. **c** Hematoxylin and eosin (H&E) staining of the small intestine from 12-week-old mice with indicated genotypes. Representative data of two independent experiments. Scale bar, 100 µm. **d** Histology score of the small intestine (*n* = 3 for each group) (*P = 0.0135). Each dot represented the data from an individual mouse. Compiled data from one experiment. **e** RORγt and CD45.1 expression in Lin^–^ cells (upper panel) or total γδT cells (lower panel) measured by flow cytometry in Ctr or *Tfam*^*fl/fl*^*Rorc-cre* mice with or without CD45.1^+^ wild-type (WT) bone marrow (BM) transfer. Representative data of two independent experiments. **f** Percentages of γδT17 cells in total γδT cells (Ctr vs. *Tfam*^*fl/fl*^*Rorc-cre*, ****P < 0.0001; Ctr + CD45.1^+^ BM vs. *Tfam*^*fl/fl*^*Rorc-cre * + CD45.1^+^ BM transfer, ***P = 0^.^0002). **g** Percentages of ILC3s in Lin^–^ cells (Ctr vs. *Tfam*^*fl/fl*^*Rorc-cre*, ****P < 0.0001; Ctr + CD45.1^+^ BM vs. *Tfam*^*fl/fl*^*Rorc-cre* + CD45.1^+^ BM, *P = 0.0382; *Tfam*^*fl/fl*^*Rorc-cre* vs. *Tfam*^*fl/fl*^*Rorc-cre* + CD45.1^+^ BM, **P = 0.0059). **h** Percentages of CD45.1^+^ ILC3s in total ILC3s (Ctr + CD45.1^+ ^BM vs. *Tfam*^*fl/fl*^*Rorc-cre* + CD45.1^+ ^BM, ***P = 0.0002). **i** Picture of the small intestine. **j** Small intestine length. Compiled data from two independent experiments in **f**–**h**, **j** (Ctr vs. *Tfam*^*fl/fl*^*Rorc-cre*, **P = 0.0037; Ctr + CD45.1^+^ BM vs. *Tfam*^*fl/fl*^*Rorc-cre* + CD45.1^+^ BM, **P = 0.0014) (*n* = 4 for Ctr with or without BM transfer groups and *n* = 5 for *Tfam*^*fl/fl*^*Rorc-cre* mice with or without BM transfer groups). **k** Picture of the small intestine of mice with indicated genotypes. Five-week-old age mice were treated with tamoxifen (2 mg/mouse by intraperitoneal injection) daily for 5 days. Data were collected 3 weeks after the last tamoxifen injection. **l** Small intestine length. Compiled data from three independent experiments (*n* = 6 for Ctr, *n* = 7 for *Tfam*^*fl/fl*^*Tcrd*^*CreER*^ mice). Ctr included *Tfam*^*+/+*^, *Tfam*^*fl/+*^, *Tfam*^*fl/fl*^*, Tfam*^*+/+*^*Rorc-cre*, and *Tfam*^*fl/+*^*Rorc-cre* mice in **b**, **d**, **f**–**h**, **j** (**P = 0.0060). Ctr included *Tfam*^*+/+*^*Tcrd*^*CreER*^ and *Tfam*^*fl/+*^*Tcrd*^*CreER*^ mice in **l**. Data are shown as mean ± SD in **b**, **d**, **f**–**h**, **j**, **l**.

Upon close examination of the intestinal phenotype of *Tfam*^*fl/fl*^*Rorc-cre* mice at different ages, we noted that compared to their littermate controls, *Tfam*^*fl/fl*^*Rorc-cre* mice developed a progressive small intestine lengthening starting from 8-week-old age (Fig. 3a, b). In contrast, the large intestine length had no difference even until 12-week-old age (Supplementary Fig. 4a). Histological analysis showed pathological changes, including increased inflammatory infiltration in intraepithelial and lamina propria compartments, goblet cell and crypt hyperplasia, as well as muscularis propria hypertrophy in the small intestine of *Tfam*^*fl/fl*^*Rorc-cre* mice (Fig. 3c, d).

To further determine the effect of Tfam deletion in T cells, we generated *Tfam*^*fl/fl*^*Cd4-cre* mice, in which Tfam was deleted in CD4^+^ and CD8^+^ T cells. Our data showed that *Tfam*^*fl/fl*^*Cd4-cre* mice had normal peripheral CD4^+^ and CD8^+^ T cells (Supplementary Fig. 4b, c). Furthermore, *Tfam*^*fl/fl*^*Cd4-cre* mice had normal ILC3s and γδT17 cells (Supplementary Fig. 4d, e). Of note, *Tfam*^*fl/fl*^*Cd4-cre* mice did not have increased small intestine length compared with their littermate controls even at older age (3-month-old) (Supplementary Fig. 4f, g). In addition, *Tfam*^*fl/fl*^*Cd4-cre* mice and *Tfam*^*fl/fl*^*Rorc-cre* mice had reduced T_reg_ cells but normal Th17 cells in the gut (Supplementary Fig. 4h–k), consistent with our previous findings showing an important role of Tfam in regulating gut T_reg_ cells^22^. Since Tfam is deleted in CD4^+^ T cells in both *Tfam*^*fl/fl*^*Cd4-cre* and *Tfam*^*fl/fl*^*Rorc-cre* mice, these data suggest that the intestine lengthening observed in *Tfam*^*fl/fl*^*Rorc-cre* mice may be independent of CD4^+^ T cells, including Th17 and T_reg_ cells.

We further transferred the congenically marked wild-type (WT) bone marrow (BM) from the adult CD45.1/CD45.1 mice to the half-lethally irradiated 4-week-old littermate control or *Tfam*^*fl/fl*^*Rorc-cre* mice and examined their phenotypes 5 months after the bone marrow transfer. γδT17 cells were not restored after WT BM transfer (Fig. 3e, f), consistent with the fact that γδT17 cells can be generated from the fetal thymus but not the adult bone marrow^47,48^. Although about twofold reduced compared to WT mice, significant restoration of ILC3s (specifically, CD45.1^+^ donor-derived) by BM transfer (Fig. 3e, g, h) could not revert the phenotype of small intestine lengthening in *Tfam*^*fl/fl*^*Rorc-cre* mice (Fig. 3i, j). Of note, these data did not rule out the contribution of ILC3s, but suggested that Tfam deficiency in γδT17 cells was likely a major contributor to small intestine lengthening in *Tfam*^*fl/fl*^*Rorc-cre* mice. This interpretation was further supported by small intestine lengthening in *Tfam*^*fl/fl*^*Tcrd*^*CreER*^ mice, which had deletion of *Tfam* in γδT cells but not in ILC3s (Fig. 3k, l and Supplementary Fig. 4l–n). Collectively, our results suggest that Tfam plays a pivotal role in regulating γδT17 cell maintenance and small intestine tissue remodeling.

### Tissue-specific transcriptome signature of γδT17 cells

**Fig. 4:**
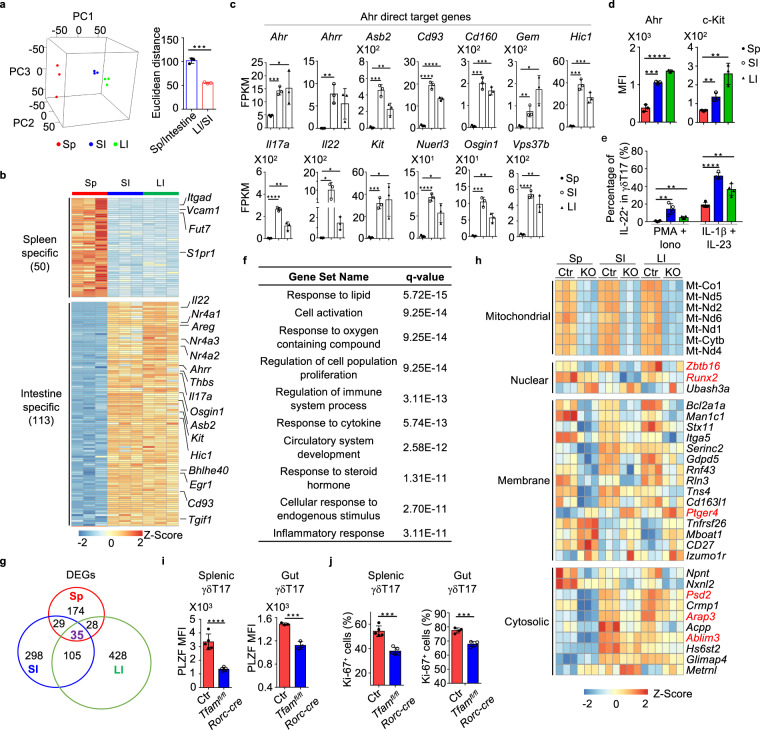
Gene profiling reveals tissue-specific transcriptomic signature of γδT17 cells. **a** Left panel, principal component analysis (PCA) plot of the RNA-seq of Tfam-sufficient splenic (Sp), small intestinal (SI), and large intestinal (LI) γδT17 cells from 3-week-old (*n* = 3 for each group) mice. γδT17 cells of each sample were sorted from an individual mouse. Right panel, Euclidean distance comparison of the transcriptome of each splenic γδT17 cell sample and the mean intestine centroid vs. the mean small intestine centroid to each large intestinal γδT17 cell sample (*n* = 3 for each group) (***P = 0.0002). **b** Heatmap of tissue-specific genes (TSGs) identified by the RNA-seq in Sp, SI, and LI γδT17 cells. Genes were ranked in a descending order based on the fold changes of expression (Sp/[SI and LI average mean]; [SI and LI average mean]/Sp). **c** RNA-seq FPKM values of Ahr direct target genes of Sp, SI, and LI γδT17 cells (mean ± SD) (*n* = 3 for each group) (Sp vs. SI: *Ahr*, ***P = 0.0005, *Ahrr*, **P = 0.0037, *Asb2*, ***P = 0.0004, *Cd93*, ****P < 0.0001, *Cd160*, ***P = 0.0002, *Gem*, **P = 0.0096, *Hic1*, ***P = 0.0002, *Il17a*, ****P < 0.0001, *Il22*, *P = 0.0180, *Kit*, ***P = 0.0004, *Nuerl3*, ****P < 0.0001, *Osgin1*, ***P = 0.0002, *Vps37b*, ****P < 0.0001; Sp vs. LI: *Ahr*, *P = 0.0460, *Ahrr*, *P* = 0.0506, *Asb2*, **P = 0.0076, *Cd93*, ****P < 0.0001, *Cd160*, ***P = 0.0001, *Gem*, *P = 0.0108, *Hic1*, ***P = 0.0008, *Il17a*, **P = 0.0071, *Il22*, *P = 0.0171, *Kit*, *P = 0.0166, *Nuerl3*, *P = 0.0137, *Osgin1*, **P = 0.0027, *Vps37b*, **P = 0.0034). FPKM, fragments per kilobase of exon model per million mapped fragments. **d** Ahr and c-Kit expression in Sp, SI, and LI γδT17 cells by flow cytometry (*n* = 4 for each group) (Sp vs. SI: Ahr, ***P = 0.0003, c-Kit, **P = 0.0049; Sp vs. LI: Ahr, ****P < 0.0001, c-Kit, **P = 0.0039). Compiled data from one experiment. **e** Percentages of IL-22^+^ cells in Sp, SI, and LI γδT17 cells by flow cytometry (*n* = 4 for each group) (Sp vs. SI: PMA + Iono, **P = 0.0046, IL-1β + IL-23, ****P < 0.0001; Sp vs. LI: PMA + Iono, **P = 0.0021, IL-1β + IL-23, **P = 0.0027). Cells were stimulated with or without PMA (20 ng/ml) plus ionomycin (Iono) (1 μg/ml) but with IL-1β (10 ng/ml) plus IL-23 (10 ng/ml) for 6 h. Compiled data from one experiment. **f** GO analysis of the 113 TSGs in γδT17 cells. **g** Pie graph of differentially expressed genes (DEGs) between Ctr and Tfam-deficient γδT17 cells from Sp, SI, and LI. **h** Heatmap of the 35 core Tfam-regulated DEGs in γδT17 cells. KO indicated *Tfam*^*fl/fl*^*Rorc-cre* mice. **i** Left panel, PLZF expression in splenic γδT17 cells from mice with indicated genotypes by flow cytometry (*n* = 5 for each group) (splenic, ****P < 0.0001; gut, ***P = 0.0008). Compiled data from two independent experiments. Right panel, PLZF expression in gut γδT17 cells from mice with indicated genotypes by flow cytometry (*n* = 3 for each group). Compiled data from one experiment. **j** Left panel, Ki-67 expression in splenic γδT17 cells from 3-week-old mice with indicated genotypes by flow cytometry (*n* = 5 for each group) (spleen, ***P = 0.0001; gut, ***P = 0.0003). Compiled data from two independent experiments. Right panel, Ki-67 expression in gut γδT17 cells from 1-week-old mice with indicated genotypes by flow cytometry (*n* = 4 for each group). Compiled data from one experiment. Ctr included *Tfam*^*fl/fl*^, *Tfam*^+/+^*Rorc-cre*, and *Tfam*^*fl/+*^*Rorc-cre* mice in **g**–**j**. Data are shown as mean ± SD in **a**, **c**–**e**, **i**, **j**.

To determine the potential tissue specificity of γδT17 cells, we performed transcriptome analyses of γδT17 cells from the spleen, small and large intestines by RNA sequencing (RNA-seq). Results showed distinct transcriptional signatures among splenic, small and large intestinal γδT17 cells (Fig. 4a, b), suggesting a tissue imprinting on γδT17 cell transcriptome. Of note, principal component analysis (PCA) indicated that the transcriptional signature of splenic γδT17 cells was more distinct from those of small and large intestinal γδT17 cells (Fig. 4a). Next, we defined the tissue-specific gene (TSG) sets among splenic, small and large intestinal γδT17 cells (criteria: FPKM ≥ 4, fold change ≥ 10, *q* ≤ 0.05). Comparing intestinal (including small and large intestines) and splenic γδT17 cells, a total of 163 TSGs (50 TSGs in the splenic γδT17 cells vs. 113 TSGs in the intestinal γδT17 cells) were identified (Fig. 4b and Supplementary Table 1). Among spleen TSGs, *S1pr1* that controls naïve T cell egress from lymph nodes, and genes involved in cell adhesion (e.g., *Itgad*, *Vcam1, Fut7)* were identified (Fig. 4b), suggesting a unique mechanism of regulation that involves migration and/or retaining γδT17 cells in the spleen.

Several observations emerged after careful assessment of intestinal γδT17 TSGs. Expression of aryl hydrocarbon receptor (*Ahr*), an environment sensor that is preferentially expressed in CD4^+^ T cells and ILCs in the gut compared with other tissues^32,33,49^, was higher in gut γδT17 cells than spleen γδT17 cells (Fig. 4c). Accordingly, a group of genes (*Ahrr, Asb2, Cd93, Cd160, Gem, Hic1, Il17a, Il22, Kit, Neurl3, Osgin1, Vps37b)*, all of which have been identified as Ahr direct targets in CD4^+^ T cells by ChIP-seq^49^, were also expressed substantially higher in the gut compared with the spleen (Fig. 4b, c). Higher expression of Ahr and c-Kit (encoded by *Kit*) in intestinal γδT17 cells compared with splenic γδT17 cells was observed at the protein level by FACS (Fig. 4d and Supplementary Fig. 5a, b). In addition, intestinal γδT17 cells (especially the small intestinal γδT17 cells) had higher IL-22 protein expression compared with splenic γδT17 cells, even upon stimulation of IL-1β and IL-23 that could substantially enhance IL-22 expression by γδT17 cells in all tissues (Fig. 4e and Supplementary Fig. 5c). Of note, genes in transforming growth factor beta (TGF-β) signaling pathway (*Tgif1* and *Thbs1* (refs. ^50–52^)) were also among the TSGs of intestinal γδT17 cells (Fig. 4b and Supplementary Fig. 5d), in line with the fact that TGF-β is highly enriched in the gut^53^. Thus, these data suggested an adaptation to the gut milieu by γδT17 cells through engagement of the Ahr pathway and TGF-β pathway.

Gene Ontology (GO) analysis of 113 intestinal TSGs and 50 splenic TSGs further showed that gut γδT17 TSGs were more enriched in cell activation, and cytokine and inflammatory responses compared with splenic TSGs (Fig. 4f and Supplementary Fig. 5e). Consistently, T cell effectors (e.g., *Il17a, Il17f*, *Il22*, and *Areg*) and activation molecules (*Bhlhe40, Egr1, Nr4a1*, *Nr4a2*, and *Nr4a3*) were among TSGs of intestinal γδT17 cells (Fig. 4b and Supplementary Table 1)^54–58^. In contrast to the distinct expression patterns of gut and spleen γδT17 cells, comparing the small and large intestinal WT γδT17 cells, a limited number of TSGs, 12 for small intestinal and 7 for large intestinal γδT17 cells, were identified (Supplementary Table 1). Together, these results suggested that intestinal γδT17 cells adopt a unique gene signature with a more activated feature compared with splenic γδT17 cells, consistent with the immunologically stimulating environment of the gut.

### Tfam deficiency affects PLZF expression and γδT17 cell proliferation

To determine the mechanisms by which Tfam deficiency affects the systemic γδT17 cell maintenance, we performed transcriptome analyses by comparing Tfam-sufficient (Ctr) and -deficient (KO) γδT17 cells and defined differentially expressed genes (DEGs, FPKM ≥ 1, fold change≥1.5, *q* ≤ 0.05) in multiple tissues. A total of 265 splenic DEGs (75 up- and 190 down-regulated genes), 467 small intestinal DEGs (224 up- and 243 down-regulated genes), and 596 large intestinal DEGs (193 up- and 403 down-regulated genes) were identified in Tfam-deficient γδT17 cells (Supplementary Fig. 5f–h and Supplementary Tables 2–4).

Examination of 35 core DEGs that were shared among splenic, small, and large intestinal DEGs (Fig. 4g) indicated pathways that were compromised by Tfam deficiency. As expected, mitochondrial genes (mt-Co1, mt-Nd1, mt-Nd2, mt-Nd4, mt-Nd5, mt-Nd6, mt-Cytb) were down-regulated in Tfam-deficient γδT17 cells in all tissues examined (Fig. 4h), consistent with the key role of Tfam in regulating mitochondrial gene transcription. We also noted an altered expression of genes involved in several fundamental biological processes, such as the actin cytoskeleton remodeling pathway for cell motility and growth and transcriptional regulation pathway for cell proliferation in Tfam-deficient γδT17 cells. For example, a group of genes involved in small GTPase signaling or related to cytoskeleton regulation (*Psd2*, *Arap3*, *Ablim3*)^59^ was down-regulated in Tfam-deficient γδT17 cells (Fig. 4h). *Ptger4*, which has been shown to suppress B cell proliferation^60^, was increased, while *Runx2*, critical for osteoblast proliferation^61^, was markedly decreased in Tfam-deficient γδT17 cells (Fig. 4h).

*Zbtb16* showed a consistent decrease in splenic, small and large intestinal Tfam-deficient γδT17 cells compared with littermate control γδT17 cells (Fig. 4h). Since promyelocytic leukemia zinc-finger protein (PLZF, encoded by *Zbtb16*) has been shown to play a key role in promoting γδT17 cell development and proliferation^45^. We further determined its protein expression by flow cytometry. PLZF expression was decreased in both splenic and intestinal Tfam-deficient γδT17 cells (Fig. 4i). Consistent with altered expression of multiple genes involved in cell proliferation, Ki-67 staining and BrdU incorporation were reduced in Tfam-deficient splenic and gut γδT17 cells (Fig. 4j and Supplementary Fig. 5i). However, BrdU-positive cells were similar between control and Tfam-deficient ILC3s at 3-week-old age (Supplementary Fig. 5j). Furthermore, BrdU incorporation in ILC3s was not decreased upon deletion of Tfam in mice at 8-week-old age, when ILC3s showed a significant reduction (Supplementary Fig. 5j and Fig. 2c), suggesting that a yet-to-be determined mechanism other than proliferation defect may lead to the decrease of ILC3s in *Tfam*^*fl/fl*^*Rorc-cre* mice. No significant difference in apoptosis was observed upon deletion of *Tfam* in γδT17 cells (Supplementary Fig. 5k, l). Together, we conclude that Tfam deficiency in γδT17 cells alters a selected set of core gene expression, resulting in defective cell proliferation.

### Tfam deficiency causes transcriptomic changes of small intestine tissue remodeling

**Fig. 5:**
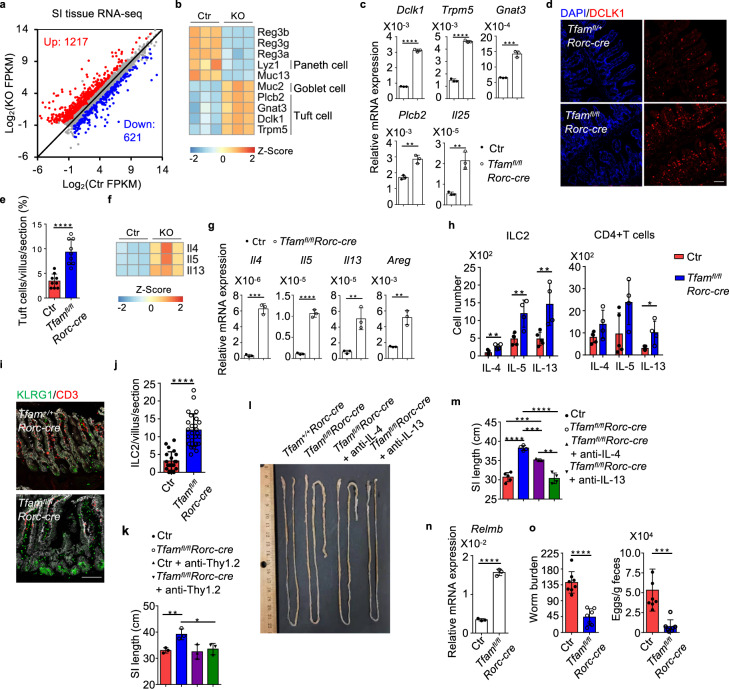
RORγt-specific ablation of Tfam leads to small intestine tissue remodeling with enhanced tuft cell–ILC2 circuit. Ctr included *Tfam*^*+/+*^*Rorc-cre* and *Tfam*^*fl/+*^*Rorc-cre* mice in **a**, **b**, **f**. KO indicated *Tfam*^*fl/fl*^*Rorc-cre* mice. **a** Differential gene expression by RNA-seq analysis of small intestine tissues of Ctr (*n* = 3) and KO mice (*n* = 3) at 12-week-old age (fold change ≥1.5, *q* value ≤0.05). **b** Heatmap of indicated gene expression from RNA-seq analysis of small intestine tissues of Ctr and KO mice. Each sample represented one individual mouse. **c** Analysis of tuft cell-related gene expression by qRT-PCR in the small intestine tissues of 12-week-old Ctr and *Tfam*^*fl/fl*^*Rorc-cre* mice (*n* = 3) (*Dclk1*, ****P < 0.0001; *Trpm5*, ****P < 0.0001; *Gnat3*, ***P = 0.0002; *Plcb2*, **P = 0.0023; *Il25*, **P = 0.0030). Representative data of three independent experiments. Ctr included *Tfam*^*+/+*^, *Tfam*^*fl/+*^, *Tfam*^*fl/fl*^, *Tfam*^*+/+*^*Rorc-cre*, and *Tfam*^*fl/+*^*Rorc-cre* mice in **c**, **e**, **g**–**o**. **d** Confocal analysis of tuft cells by DCLK1 staining in the small intestine (ileum) of 12-week-old mice with indicated genotypes. Red, DCLK1 staining. Blue, DAPI nuclear staining. Scale bar, 200 µm. Representative data from three independent experiments. **e** Quantification of tuft cells (DCLK1^+^) in each small intestine villus viewed by confocal microscopy (*n* = 9 villi for each group) (****P < 0.0001). Compiled data from two independent experiments. **f** Heatmap of indicated gene expression from RNA-seq analysis of small intestine tissues of Ctr and KO mice. **g** Analysis of *Il4*, *Il5*, *Il13*, and *Areg* expression by qRT-PCR in small intestine tissues of 12-week-old Ctr and *Tfam*^*fl/fl*^*Rorc-cre* mice (*n* = 3) (*Il4*, ***P = 0.0001; *Il5*, ****P < 0.0001; *Il13*, **P = 0.0066; *Areg*, **P = 0.0023). Representative data of three independent experiments. **h** IL-4, IL-5, and IL-13 expression in ILC2s or CD4^+^ T cells by flow cytometry from the small intestine draining lymph nodes (siLN) of Ctr (*n* = 5) and *Tfam*^*fl/fl*^*Rorc-cre* (*n* = 4) mice at 12-week-old age (ILC2: IL-4, **P = 0.0082; IL-5, **P = 0.0064; IL-13, **P = 0.0097; CD4^+ ^T cells: IL-4, *P* = 0.0929; IL-5, *P* = 0.0652; IL-13, *P = 0.0194). Compiled data from two independent experiments. **i** Confocal analysis of ILC2s (CD3^–^KLRG1^+^) in the small intestine (ileum) of 12-week-old mice with indicated genotypes. Red, CD3 staining. Green, KLRG1 staining. White, EpCAM staining. Scale bar, 200 µm. Representative data of three independent experiments. **j** Quantification of ILC2s (CD3^–^KLRG1^+^) in each small intestine villus viewed by confocal microscopy (****P < 0.0001). Ctr, *n* = 21, *Tfam*^*fl/fl*^*Rorc-cre*, *n* = 22. Compiled data from two independent experiments. **k** Small intestine length in Ctr or *Tfam*^*fl/fl*^*Rorc-cre* mice treated with anti-Thy1.2 neutralizing antibody for 3 weeks (*n* = 3 for each group) (Ctr vs*. Tfam*^*fl/fl*^*Rorc-cre*, **P = 0.0098; *Tfam*^*fl/fl*^*Rorc-cre* vs. *Tfam*^*fl/fl*^*Rorc-cre* + anti-Thy1.2, *P = 0.0312). Compiled data from two independent experiments. **l** Picture of small intestines in mice of indicated genotypes treated with anti-IL-4 antibody (100 µg/mouse) or anti-IL-13 antibody (50 µg/mouse) twice a week from 6-week-old age for 3 weeks by intraperitoneal cavity injection. Representative data of two independent experiments. **m** Small intestine length. Compiled data from two independent experiments (Ctr vs. *Tfam*^*fl/fl*^*Rorc-cre*, ****P < 0.0001; Ctr vs. *Tfam*^*fl/fl*^*Rorc-cre* + anti-IL-4, ***P = 0.0009; *Tfam*^*fl/fl*^*Rorc-cre* vs. *Tfam*^*fl/fl*^*Rorc-cre* + anti-IL-4, ***P = 0.0006; *Tfam*^*fl/fl*^*Rorc-cre* vs. *Tfam*^*fl/fl*^*Rorc-cre* + anti-IL-13, ****P < 0.0001; *Tfam*^*fl/fl*^*Rorc-cre* + anti-IL-4 vs. *Tfam*^*fl/fl*^*Rorc-cre* + anti-IL-13, **P = 0.0022). *n* = 5 for Ctr, *n* = 4 for *Tfam*^*fl/fl*^*Rorc-cre* mice; *n* = 3 for *Tfam*^*fl/fl*^*Rorc-cre* mice treated with anti-IL-4; *n* = 4 for *Tfam*^*fl/fl*^*Rorc-cre* mice treated with anti-IL-13. **n** Analysis of *Relmb* expression by qRT-PCR in the small intestine tissues of 12-week-old Ctr and *Tfam*^*fl/fl*^*Rorc-cre* mice (*n* = 3) mice at 14 days after *Hpb* infection (****P < 0.0001). Representative data of three independent experiments. **o** Left panel, *Hpb* worm burden in small intestine lumen of Ctr (*n* = 8) and *Tfam*^*fl/fl*^*Rorc-cre* mice (*n* = 7) at 14 days after *Hpb* infection (****P < 0.0001). Right panel, *Hpb* egg counts per gram feces in Ctr (*n* = 8) and *Tfam*^*fl/fl*^*Rorc-cre* mice (*n* = 7) mice at 14 days after *Hpb* infection (***P = 0.0008). Compiled data from three independent experiments. Data are shown as mean ± SD in **c**, **e**, **g**, **h**, **j**, **k**, **m**–**o**. *, **, ***, **** indicated *P* values ≤0.05, 0.01, 0.001, 0.0001, respectively.

To determine potential mechanisms underlying the lengthened small intestine in *Tfam*^*fl/fl*^*Rorc-cre* mice, we performed small intestine tissue gene expression profiling by RNA-seq. A total of 1838 DEGs (FPKM ≥ 1, fold change ≥ 1.5, *q* ≤ 0.05) were identified, with 1217 genes up-regulated and 621 genes down-regulated in small intestine tissue of *Tfam*^*fl/fl*^*Rorc-cre* mice compared with control mice (Fig. 5a, Supplementary Fig. 6a and Supplementary Table 5). Among them, genes involved in insulin growth factor (*Igf*) signaling (i.e., *Igf1, Igf2*, *Igf1r*, *Igf2r*, *Igfbp2*, *Igfbp4*, *Igfbp6*, and *Igfbp7*), which have been shown to promote intestine epithelial proliferation^62^, were significantly up-regulated (Supplementary Fig. 6b, c), and thus potentially contributed to the increased small intestine length in *Tfam*^*fl/fl*^*Rorc-cre* mice. Genes expressed by secretory epithelial cells were also changed in *Tfam*^*fl/fl*^*Rorc-cre* mice. For example, *Reg3a*, *Reg3b*, *Reg3g*, target genes of IL-22 (ref. ^63^), were down-regulated in small intestinal tissue of *Tfam*^*fl/fl*^*Rorc-cre* mice compared with littermate control mice (Fig. 5b), consistent with the reduction of IL-22-producing cells (ILC3s and γδT17 cells). *Lyz1*, which encodes the Paneth cell-specific lysozyme, was also decreased in the small intestine of *Tfam*^*fl/fl*^*Rorc-cre* mice (Fig. 5b). In contrast, *Muc2*, which is expressed by goblet cells, was increased in the small intestine of *Tfam*^*fl/fl*^*Rorc-cre* mice (Fig. 5b). Consistently, periodic acid–Schiff (PAS) staining further indicated an increase of goblet cells in the small intestine of *Tfam*^*fl/fl*^*Rorc-cre* mice (Supplementary Fig. 6d). Of note, several genes that are highly expressed by tuft cells (e.g., *Dclk1*, *Trpm5*, *Gnat3*, *Plcb2*, and *Il25*) were up-regulated in the small intestine of *Tfam*^*fl/fl*^*Rorc-cre* mice (Fig. 5b, c), suggesting an increase of tuft cells in the small intestine of *Tfam*^*fl/fl*^*Rorc-cre* mice. Tuft cell enhancement was further validated by confocal microscopy analysis of DCLK1 expression (Fig. 5d, e). Together, these results demonstrated that genetic ablation of Tfam in RORγt^+^ lymphocytes causes small intestine tissue transcriptomic changes associated with increased tissue remodeling.

### Tfam deficiency in RORγt^+^ lymphocytes enhance type 2 immunity in the small intestine

A recent study showed that increased tuft cell–ILC2 circuit drives small intestine lengthening in mice deficient in *Tnfaip3* (encodes A20), a negative regulator of ILC2s^38^, phenocopying *Tfam*^*fl/fl*^*Rorc-cre* mice. Indeed, a marked enhancement of type 2 immune genes (*Il4*, *Il5*, *Il13,* and *Areg*) was observed in the small intestine tissues of *Tfam*^*fl/fl*^*Rorc-cre* mice compared with littermate control mice (Fig. 5f, g). Accordingly, type 2 immune cells, especially ILC2s, were increased in *Tfam*^*fl/fl*^*Rorc-cre* mice (Fig. 5h and Supplementary Fig. 6e). Confocal staining further confirmed a significant increase of ILC2s in the small intestine lamina propria of *Tfam*^*fl/fl*^*Rorc-cre* mice compared with littermate control mice (Fig. 5i, j). Anti-Thy1.2 neutralizing antibody treatment, which depletes Thy1.2^+^ lymphocytes including ILC2s, fully restored the small intestine length of *Tfam*^*fl/fl*^*Rorc-cre* mice to the level of littermate control mice (Fig. 5k). In addition, in vivo neutralizing type 2 cytokines (IL-4 or IL-13) either partially or completely restored the small intestine length of *Tfam*^*fl/fl*^*Rorc-cre* mice (Fig. 5l, m). Together, these data suggest that Tfam in γδT17 cells regulates small intestine tissue remodeling through a type 2 immunity-dependent manner under steady-state condition.

It was reported that the regulation of small intestine length by tuft-ILC2 cell circuit was dependent on the *Tritrichomonas* spp. (a protozoa sensitive to antibiotics metronidazole (MNZ) treatment)^38^. Intriguingly, the amounts of *Tritrichomonas* spp. had no difference or were even decreased without statistical significance in *Tfam*^*fl/fl*^*Rorc-cre* mice at various ages, including when the mice developed lengthened small intestine (Supplementary Fig. 6f). However, consistent with the literature^38^, MNZ treatment reduced *Tritricomonas* spp. and abrogated the phenotypes of increased small intestine length (Supplementary Fig. 6g–i). These data suggested that the MNZ-sensitive microbiome and/or eukaryome may be responsible for the intestinal phenotypes observed in *Tfam*^*fl/fl*^*Rorc-cre* mice.

Enhanced type 2 immunity in the small intestine of *Tfam*^*fl/fl*^*Rorc-cre* mice was also evident during infection. Upon infection of *Heligmosomoides polygyrus bakeri* (*Hpb*), increased ILC2s and Th2 cells in the siLN of *Tfam*^*fl/fl*^*Rorc-cre* mice were observed compared with littermate control mice (Supplementary Fig. 6j). There was higher *Relmb* expression (encoding resistin-like molecule β, a protein involved in protection against infection by parasitic helminth^32^) in the small intestine of *Tfam*^*fl/fl*^*Rorc-cre* mice after *Hpb* infection (Fig. 5n). As a result, *Tfam*^*fl/fl*^*Rorc-cre* mice had lower worm burden in the small intestine and fewer egg counts in feces compared to littermate control mice (Fig. 5o). Together, these results demonstrate that *Tfam*^*fl/fl*^*Rorc-cre* mice had enhanced type 2 immune responses (e.g., ILC2s and Th2 cells) that lead to better anti-helminth immunity.

### IL-22 suppresses IL-13-induced tuft cell differentiation

**Fig. 6:**
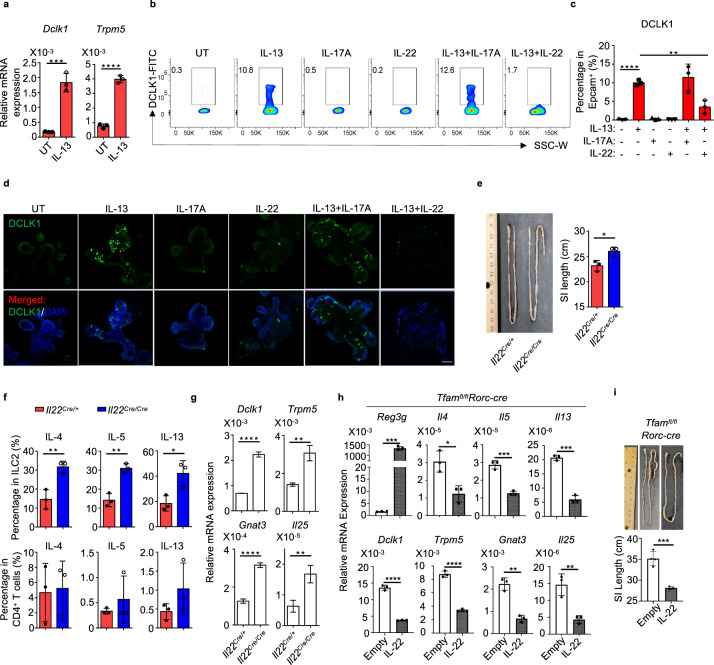
IL-22 suppresses IL-13-induced tuft cell differentiation. **a** Analysis of *Dclk1* and *Trpm5* expression by qRT-PCR in enteroid culture (*n* = 3) (mean ± SD) (*Dclk1*, ***P = 0.0008; *Trpm5*, ****P < 0.0001). Representative data of three independent experiments. **b** DCLK1 expression in enteroid culture with indicated treatment (IL-13 (50 ng/ml), IL-17A (100 ng/ml), and/or IL-22 (5 ng/ml)) measured by flow cytometry. Representative data of two independent experiments. **c** Percentages of DCLK1^+^ cells in EpCAM^+^ cells (mean ± SD) (*n* = 3) (none vs. IL-13, ****P < 0.0001; IL-13 vs. IL-13 + IL-22, **P = 0.0045). Compiled data from one experiment. **d** Confocal analysis of DCLK1 expression in enteroid culture with indicated treatment. Representative data of two independent experiments. Green, DCLK1. Blue, DAPI. Scale bar: 50 µm. **e** Left panel, picture of the small intestine of 8-week-old littermate *Il22*^*Cre*/+^ and *Il22*^*Cre/Cre*^ mice ^(^*P = 0.0223). Representative data of two independent experiments. Right panel, small intestine length in mice with indicated genotypes (*n* = 3). Compiled data from two independent experiments. **f** IL-4, IL-5, and IL-13 expression in ILC2s or CD4^+^ T cells by flow cytometry from the small intestine of 8-week-old *Il22*^*Cre/+*^ and *Il22*^*Cre/Cre*^ mice (*n* = 3) (ILC2: IL-4, **P = 0.0076; IL-5, **P = 0.0024; IL-13, *P = 0.0245; CD4^+ ^T: IL-4, *P* = 0.8613; IL-5, *P* = 0.4343; IL-13, *P* = 0.2181). Compiled data from two independent experiments. **g** Analysis of *Dclk1*, *Trpm5*, *Gnat3,* and *Il25* expression by qRT-PCR in small intestine tissues of 8-week-old *Il22*^*Cre/+*^ and *Il22*^*Cre/Cre*^ mice (*n* = 3) (*Dclk1*, ****P < 0.0001; *Trpm5*, **P = 0.0023; *Gnat3*, ****P < 0.0001; *Il25*, **P = 0.0062). Representative data of two independent experiments. **h**, **i** *Tfam*^*fl/fl*^*Rorc-cre* mice at the age of 6 weeks old received hydrodynamic tail vein injection of either empty or IL-22 vectors. Mice were sacrificed at 10 weeks of age and small intestine tissues were harvested. **h** qRT-PCR gene expression analysis of small intestine tissue IL-22-target gene *Reg3g*, type 2 cytokine genes *Il4*, *Il5*, and *Il13*, and tuft cell-associated genes *Dclk1*, *Trpm5*, *Gnat3*, and *Il25* (*n* = 3) (*Reg3g*, ***P = 0.0001; *Il4*, *P = 0.0139; *Il5*, ***P = 0.0004; *Il13*, ***P = 0.0001; *Dclk1*, ****P < 0.0001; *Trpm5*, ****P < 0.0001; *Gnat3*, **P = 0.0011; *Il25*, **P = 0.0063). Representative data of three independent experiments. **i** Top panel, picture of the small intestine length. Bottom panel, quantification of the small intestine length (*n* = 4 for empty group and *n* = 3 for IL-22 treatment group) (***P = 0.0009). Compiled data from one experiment. Data are shown as mean ± SD in **a**, **c**, **e**–**i**.

Type 2 cytokine IL-13 has been shown to promote tuft cell differentiation, highlighting the importance of tuft cell–ILC2 circuit in small intestine tissue remodeling^35–37^. As gut γδT17 cells produce large amounts of IL-22 (ref. ^64^), we hypothesized that IL-22 may directly inhibit the IL-13-induced tuft cell differentiation. As expected, IL-13 treatment enhanced tuft cell differentiation indicated by induction of *Dclk1* and *Trpm5* expression in the in vitro enteroid culture model (Fig. 6a and Supplementary Fig. 7). The increase of DCLK1 expression by IL-13 treatment was further corroborated at the protein level by flow cytometry and confocal microscopy analyses (Fig. 6b–d). Notably, IL-22 but not IL-17A, both of which are highly expressed by γδT17 cells, decreased DCLK1 expression induced by IL-13 (Fig. 6b–d), suggesting that IL-22 can inhibit IL-13-induced tuft cell differentiation in vitro. Consistently, *Il22* knockout mice (*Il22*^Cre/Cre^ knockout/in mice) showed longer small intestine length compared to their control littermates (*Il22*^Cre/+^) (Fig. 6e). Significant enhancement of ILC2s but not Th2 cells together with increased tuft cell gene expression (i.e., *Dck1*, *Trpm5*, *Gnat3*, and *Il25*) were observed in the small intestine of *Il22* knockout mice (Fig. 6f, g). More importantly, overexpression of IL-22 in vivo in *Tfam*^*fl/fl*^*Rorc-cre* mice by hydrodynamic injection reduced the expression of tuft cell and type 2 immune genes and accordingly restored the small intestine length (Fig. 6h, i). Together, these results suggest that the phenotypes of small intestine tissue remodeling upon ablation of Tfam in RORγt^+^ lymphocytes are at least in part due to lack of IL-22, a cytokine that suppresses the intestinal tuft cell–ILC2 circuit.

### *Tfam* deletion in RORγt^+^ lymphocytes lead to global metabolomic changes in the small intestine

Both GO and KEGG analyses of small intestine tissue RNA-seq data showed enrichment of DEGs associated with metabolism (e.g., small-molecule metabolic process, amino acid and lipid metabolic processes, and organic acid metabolic process) (Supplementary Fig. 8a, b). The dysregulated gene expression in metabolic pathways prompted us to directly assess the small intestine tissue metabolic changes in *Tfam*^*fl/fl*^*Rorc-cre* mice by global metabolome analysis.

**Fig. 7:**
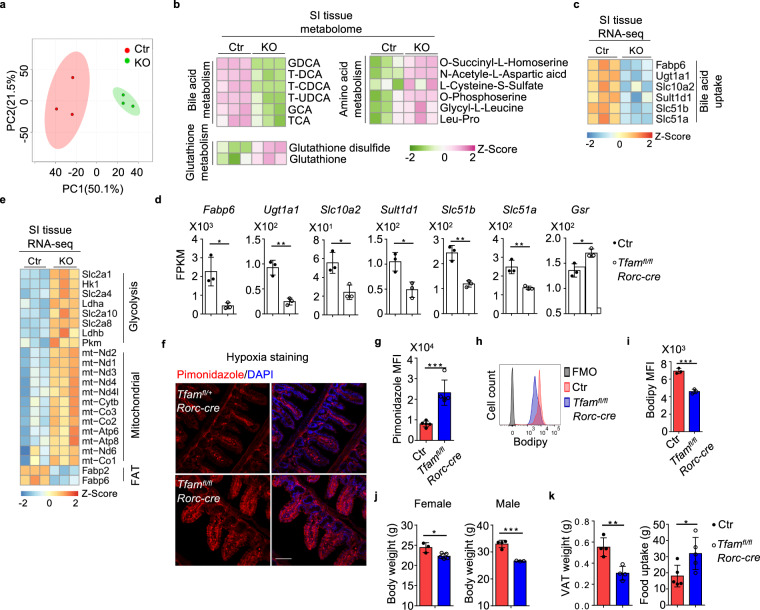
Tfam deletion in RORγt^+^ lymphocytes lead to global metabolomic changes in the small intestine. Ctr included *Tfam*^*+/+*^*Rorc-cre* and *Tfam*^*fl/+*^*Rorc-cre* mice, while KO indicated *Tfam*^*fl/fl*^*Rorc-cre* mice in **a**–**e**. **a** PCA analysis of the metabolome of small intestine tissues of 12-week-old Ctr and KO mice (*n* = 3 for each group). Each group included three individual mice. **b** Heatmap of the differentially regulated metabolites between small intestine tissues of Ctr and KO groups identified by the metabolome analysis. Metabolites were ranked in a descending order based on the fold changes of abundance (Ctr/KO; KO/Ctr). **c** Heatmap of indicated gene expression of small intestine tissue RNA-seq of Ctr and KO mice as shown in Fig. 5a. Genes were ranked in a descending order based on the fold changes of expression (Ctr/KO). **d** RNA-seq FPKM values of indicated genes in small intestine tissues of the mice (*n* = 3) (*Fabp6*, *P = 0.0155; *Ugt1a1*, **P = 0.0019; *Slc10a2*, *P = 0.0162; *Sult1d1*, *P = 0.0158; *Slc51b*, **P = 0.0027; *Slc51a*, **P = 0.0066; *Gsr*, *P = 0.0186). **e** Heatmap of DEGs in small intestine tissues of the mice identified by RNA-seq (*n* = 3). Genes were ranked in a descending order based on the fold changes of expression (KO/Ctr). Ctr included *Tfam*^*+/+*^, *Tfam*^*fl/+*^, *Tfam*^*fl/fl*^, *Tfam*^*+/+*^*Rorc-cre*, and *Tfam*^*fl/+*^*Rorc-cre* mice in **g**–**k**. **f** Confocal analysis of hypoxia by pimonidazole staining in the small intestine (ileum) of 12-week-old mice with indicated genotypes. Red, pimonidazole staining. Blue, DAPI staining. Scale bar, 200 µm. Representative data of three independent experiments. **g** Pimonidazole MFI in the epithelial cells shown in **f** (*n* = 5 for each group) (***P = 0.0006). Compiled data from one experiment. **h** Bodipy uptake in small intestine epithelial cells from Ctr and *Tfam*^*fl/fl*^*Rorc-cre* mice measured by flow cytometry. Representative data of two independent experiments. **i** Bodipy MFI in the epithelial cells as shown in **h** (*n* = 3 for each group) (***P = 0.0006). Compiled data from one experiment. **j** Body weight of 5-month-old female Ctr (*n* = 3) and *Tfam*^*fl/fl*^*Rorc-cre* mice (*n* = 5) and male Ctr (*n* = 4) and *Tfam*^*fl/fl*^*Rorc-cre* mice (*n* = 3) (female, *P = 0.0169; male, ***P = 0.0006). Compiled data from two independent experiments. **k** Visceral adipose tissue (VAT) weight (*n* = 4) and 4-day-food uptake (*n* = 5) in 5-month-old mice with indicated genotypes (VAT, **P = 0.0039; food uptake, *P = 0.0321). Compiled data from two independent experiments. Data are shown as mean ± SD in **d**, **g**, **i**–**k**.

PCA showed distinct metabolic profiles between the small intestine tissues of littermate control and *Tfam*^*fl/fl*^*Rorc-cre* mice (Fig. 7a). A total of 42 annotated metabolites (among a total of 1498 detected metabolites) were identified as differentially regulated between the small intestine tissues of littermate control and *Tfam*^*fl/fl*^*Rorc-cre* mice (fold change ≥2, *P* ≤ 0.05) (Fig. 7b and Supplementary Fig. 8c). These metabolites could be categorized into several pathways such as bile acid metabolism, glutathione metabolism, and amino acid metabolism (Fig. 7b). Of note, multiple forms of bile acids were markedly reduced in the small intestine tissues of *Tfam*^*fl/fl*^*Rorc-cre* mice (Fig. 7b and Supplementary Fig. 8c, d). Since bile acids are synthesized in the liver, secreted into bile and most of them (95%) are reabsorbed in the small intestine^65^, the reduction of bile acids could be a result of their compromised reabsorption in *Tfam*^*fl/fl*^*Rorc-cre* mice that had small intestine tissue remodeling. Consistent with this notion, the expression of several genes involved bile acid uptake (i.e., *Fabp6*, *Ugt1a1*, *Slc10a2*, *Sult1d1*, *Slc51b*, *Slc51a*)^66^ were significantly decreased in the small intestine tissues of *Tfam*^*fl/fl*^*Rorc-cre* mice (Fig. 7c, d).

Metabolome analysis also identified that several metabolites involved in glutathione metabolism (e.g., glutathione (GSH) and glutathione-disulfide (GSSG)) as well as mitochondria oxidative phosphorylation (e.g., α-ketoglutarate and 2-hydroxyglutarate) were increased in the small intestine tissues of *Tfam*^*fl/fl*^*Rorc-cre* mice (Fig. 7b and Supplementary Fig. 8c). Expression of glutathione reductase (*Gsr*), a key enzyme to produce glutathione in the cells, was increased in the small intestine tissues of *Tfam*^*fl/fl*^*Rorc-cre* mice (Fig. 7d), suggesting a compensatory elevation of antioxidants to protect tissue damage and inflammation, presumably due to oxidative stress. Indeed, several key components involved glycolysis (*Slc2a1*, *Slc2a4*, *Slc2a8*, *Hk1*, *Pkm*, *Ldha*, *Ldhb*) and most of the mitochondrial genes were up-regulated in the small intestine tissues of *Tfam*^*fl/fl*^*Rorc-cre* mice compared with littermate control mice (Fig. 7e), which could lead to enhanced oxidative stress. In line with increased tissue glycolysis and oxidative phosphorylation (OXPHOS) that consume oxygen, more hypoxic milieu was detected in the small intestine epithelial cells of *Tfam*^*fl/fl*^*Rorc-cre* mice compared with their littermate controls as measured by pimonidazole staining (Fig. 7f, g).

RNA-seq results further showed that the fatty acid uptake transporters (FAT), *Fabp2* and *Fabp6* (a gene also involved in bile acid transport^67^), were down-regulated in the small intestine tissues of *Tfam*^*fl/fl*^*Rorc-cre* mice compared with littermate control mice (Fig. 7c, e). Fatty acid uptake, indicated by Bodipy FL C16 (a fluorescence fatty acid analog), was reduced in the small intestine epithelial cells of *Tfam*^*fl/fl*^*Rorc-cre* mice (Fig. 7h, i). Consistently, *Tfam*^*fl/fl*^*Rorc-cre* mice showed a reduction of body weight and fat weight, despite of increased food intake compared with littermate control mice (Fig. 7j, k). Collectively, our results supported a model that gut tissue microenvironment shapes γδT17 cell metabolic and transcriptional programs. Metabolic changes in the small intestine tissues of *Tfam*^*fl/fl*^*Rorc-cre* mice may cause alteration of global gene expression, which reciprocally influences tissue metabolism, remodeling, and inflammation (Supplementary Fig. 8e).

## Discussion

Metabolism, such as glycolysis and mitochondrial OXPHOS, is not merely for energy generation, but also generates metabolites critical for immune cell differentiation, maintenance, and/or function^1^. Direct measurement of metabolome, oxygen consumption rate (OCR), and extra-cellular acidification rate (ECAR) in rare immune cells such as γδT cells, especially γδT17 cells, has been technically challenging due to limited cell number recovered from mice (about 10,000 γδT17 cells per mouse spleen). In this study, we took a genetic approach to ablate *Tfam*, a crucial transcription factor to regulate mitochondrial respiration gene replication and expression, in γδT cells and RORγt^+^ lymphocytes using*Tfam*^*fl/fl*^*Tcrd*^*CreER*^ and *Tfam*^*fl/fl*^*Rorc-cre* mice, respectively. Our data showed that Tfam-mediated mitochondrial metabolism was important for the maintenance of γδT17 cells and ILC3s, but not for non-γδT17 γδT cells (including γδT1 cells) or Th17 cells. Our work further revealed a critical role of Tfam in γδT17 cells to control small intestine tissue remodeling and metabolism.

Mechanistically, our data support a model that Tfam is essential for the maintenance of γδT17 cells. Consistent with this hypothesis, decreased proliferation of γδT17 cells without changes in apoptosis was observed in *Tfam*^*fl/fl*^*Rorc-cre* mice. Transcription factor PLZF has been shown to be critical for γδT17 cell development and proliferation^45^. PLZF expression was decreased in Tfam-deficient γδT17 cells. The critical role of Tfam in RORγt^+^ γδT17 cell and ILC3 maintenance was further supported by the progressive reduction of these cells in *Tfam*^*fl/fl*^*Rorc-cre* mice. Of note, we cannot rule out the possibility that Tfam regulates γδT17 cell development due to the short window of γδT17 cell development in the fetal thymus^47^, and the restricted deletion of Tfam in RORγt-expressing γδT cells in *Tfam*^*fl/fl*^*Rorc-cre* mice. Although our genetic approaches using *Tcrd*^*CreER*^ and *Rorc-cre* mice allowed us to focus on the regulation of γδT17 cell maintenance, a recent study showed that *Il7r-cre* deletion of *Maf* severely affected the γδT17 cell development^68^, warranting future detailed investigation of role of Tfam-mediated mitochondrial respiration in γδT17 cell development.

The small intestine tissue remodeling (e.g., increased tuft cells) in *Tfam*^*fl/fl*^*Rorc-cre* mice was accompanied by small intestine lengthening and inflammation (e.g., enhanced type 2 responses), consistent with the crucial role of tuft cell–ILC2 circuit in regulation of small intestine tissue homeostasis^38^. It remains to be determined whether small intestine length will change in *Rorc*^*gfp/gfp*^ mice, which lack all RORγt-expressing cells, include Th17 cells, γδT17 cells, and ILC3s. However, these mice also develop increased incidence of thymoma with age, complicating the interpretation of age-dependent small intestine lengthening and tissue remodeling. The small intestine lengthening was observed in *Tfam*^*fl/fl*^*Rorc-cre* but not in *Tfam*^*fl/fl*^*Cd4-cre* mice, suggesting that this phenotype may be CD4^+^ T cell independent in *Tfam*^*fl/fl*^*Rorc-cre* mice. Furthermore, *Tfam*^*fl/fl*^*Rorc-cre* mice had normal Th17 cells but reduced γδT17 cells and ILC3s. Together, these data prompt us to conclude that *Rorc-cre*-mediated deletion of Tfam have γδT17 and ILC3 cell type-specific effects associated with small intestine lengthening. However, our data do not rule out the possibility that the genetic strategy using *Rorc-cre* could have cell-extrinsic or secondary effects on other cell types, which in turn affect small intestine length in vivo. In contrast to bone marrow transfer that at least partially restored the ILC3 compartment, we had not been able to restore γδT17 cells in *Tfam*^*fl/fl*^*Rorc-cre* mice by adoptive transfer of purified wild-type γδT17 cells. Thus, whether γδT17 cells alone or together with other RORγt^+^ cells (i.e., ILC3s) to regulate the small intestine tissue remodeling and lengthening needs to be carefully determined.

Several potential mechanisms may count for increased type 2 immune responses (e.g., IL-13 production by ILC2s) in the small intestine of *Tfam*^*fl/fl*^*Rorc-cre* mice. Our data showed that IL-22 could inhibit the IL-13-induced tuft cell differentiation in in vitro enteroid culture. Thus, reduction of IL-22-producing γδT17 and ILC3s may relieve this negative regulation in vivo and enhances the differentiation of tuft cells, which have been shown to secrete IL-25 to promote the type 2 immune response in the gut^35–38^. In addition, mechanisms that regulate the balance of ILC2s and ILC3s in the gut have also been reported. For example, Ahr deficiency can increase ILC2s at the expense of ILC3s^32,34^. Vasoactive intestinal peptide (VIP) has been shown to regulate gut ILC2–ILC3 balance^69–71^. Thus, the reduction of γδT17 cells and ILC3s may also create a niche for expansion of type 2 immune responses in vivo. It was reported that IL-22 could activate ILC3s indirectly by inducing epithelial IL-18 during infection and IL-25-expressing epithelial cells could suppress IL-22 production by ILC3s^72,73^. Our RNA-seq analysis showed that *Il18* was indeed reduced in the small intestine of *Tfam*^*fl/fl*^*Rorc-cre* mice. However, because of normal production of IL-22 by Tfam-deficient ILC3s on a per cell basis, the mechanisms involving IL-18 or IL-25-mediated inhibition of IL-22 expression by ILC3s are less likely.

The intracellular parasite *Tritrichomonas* spp. has been shown to promote the intestinal tuft cell–ILC2 circuit by secreting succinate, a metabolite acting on the tuft cells through the succinate receptor 1 (Sucnr1)^74^. Consistently, MNZ treatment reduced *Tritricomonas* spp. and abrogated the phenotypes of increased small intestine length in *Tfam*^*fl/fl*^*Rorc-cre* mice. Of note, increased small intestine length was observed in *Tfam*^*fl/fl*^*Rorc-cre* mice compared with cohoused Tfam-sufficient littermate mice, suggesting that the phenotype could not be simply transferrable by microbiome/eukaryome. Future studies involve metagenomic studies to carefully determine the potential dysbiosis in *Tfam*^*fl/fl*^*Rorc-cre* mice and the impact of Tfam on the composition of gut microbiota, especially small intestinal flora. Also, the requirement or contribution of *Tritrichomonas* spp. to observed phenotypes of *Tfam*^*fl/fl*^*Rorc-cre* mice needs to be investigated, which requires the development of germ-free *Tfam*^*fl/fl*^*Rorc-cre* mice with mono-association of *Tritrichomonas* spp. and/or introduction of *Tritrichomonas*-free microbiome. Furthermore, bile acid metabolites changes in the small intestine tissues may also be due to altered microbiota-derived bile acid metabolism in these mice. Thus, future detailed investigation is needed to understand the role of the microbiome and/or eukaryome including *Tritrichomonas* spp. in regulation of small intestine tissue homeostasis in *Tfam*^*fl/fl*^*Rorc-cre* mice.

It has been shown that oxidative stress plays an essential role in the pathogenesis and progression of inflammatory bowel disease^75^. Global metabolome study of small intestine tissues of *Tfam*^*fl/fl*^*Rorc-cre* mice indicated an enhanced oxidative stress and hypoxic environment in the gut. Although up-regulated type 2 immune responses led to better helminth clearance, the enhanced inflammation in the small intestine of *Tfam*^*fl/fl*^*Rorc-cre* mice under the steady state might further exacerbate metabolic imbalance and worsen tissue remodeling during inflammatory challenge. The tissue transcriptome and metabolome analyses of *Tfam*^*fl/fl*^*Rorc-cre* mice further showed decreased uptake of fatty acids and bile acids in the small intestine, consistent with less body weight. The increased small intestine length may thus reflect an adaptation to the energy and nutrition malfunction due to perturbation of mitochondrial activity in *Tfam*^*fl/fl*^*Rorc-cre* mice.

In summary, our study highlighted the essential role of Tfam in the maintenance of selected IL-17-producing immune cells (γδT17 and ILC3s), and may serve as a model for further investigations to elucidate the role of mitochondrial metabolism in these cells. Understanding metabolic regulation of the gut immune-epithelial cell interactions may provide therapeutic targets to control the small intestine tissue homeostasis and immunity in health and disease.

## Methods

### Mice

Mice used in this study were maintained in specific-pathogen-free (SPF) conditions at the University of Florida. All mouse studies were approved by the Animal Care and Use Committees of the University of Florida. Mice were littermate controlled and both male and female mice were used for experiments. Mice were used at 6- to 8-week-old age unless otherwise noted. *Tfam*^*fl/fl*^ mice were kindly provided by Navdeep Chandel (Northwestern University), as previously described^22^. C57BL/6, *Tcrd*^*CreER*^ mice, *Il22*^*Cre*^ mice, and *Rorc-cre* mice were purchased from Jackson Laboratory. For *Tfam*^*fl/fl*^*Tcrd*^*CreER*^ mice, Tamoxifen was administered daily for 5 days at 2 mg/mouse by intraperitoneal injection starting from 5-week-old age. For bone marrow transfer experiments, recipient mice were irradiated with 5 Gy gamma-ray (half lethal) followed by bone marrow transfer (5 × 10^5^ cells per recipient) on the same day. Then, recipient mice received drinking water supplemented with 1 g/L ampicillin for 2 weeks. For BrdU incorporation experiment, 2 mg BrdU were i.p. injected into each mouse with indicated genotypes twice with a 12-h interval. Mice were sacrificed 12 h after the second BrdU injection. For metronidazole treatment, 5–6-week-old mice with indicated genotypes were treated with drinking water supplemented with 2.5 g/L metronidazole for 3 weeks. BrdU staining in lymphocytes were performed with Phase-Flow™ Alexa Fluor® 647 BrdU Kit (Biolegend, Cat#370706). For *Hpb* infection experiments, mice were gavaged with 200 *Hpb* infective third stage larvae (L3), a gift kindly provided by Joseph F Urban Jr. (USDA/ARS) and sacrificed at day14 after the infection. For anti-Thy1.2, anti-IL-4, and anti-IL-13 treatment experiments, mice were i.p. injected with anti-Thy1.2 (BioXCell, Cat# BE0066, 250 µg/mouse), anti-IL-4 (BioXCell, Cat# BE0045, 100 µg/mouse), or anti-IL-13 (InvivoGen, Cat# mabg-mil13, 50 µg/mouse) twice a week for 3 weeks.

### Lymphocyte isolation and flow cytometry

Lymphocytes from thymus, spleen, peripheral lymph nodes, intestinal lamina propria, lung, fat, and skin were isolated as previously described^22^. Liver lymphocyte isolation were conducted by mechanically dissociation of liver tissues after perfusion of the mouse with 10 ml sterile ice-cold phosphate-buffered saline (PBS) and then lysed with the red blood cell lysis buffer for 2 min. The cells from each tissue then went through a 70 µm filter (Sigma). The filtered cells from the thymus, spleen, and peripheral lymph nodes were ready to use for flow cytometry. The filtered cells from the liver, intestinal lamina propria, lung, fat, and skin were further purified by 37.5% and 75% Percoll gradient for 20 min spin at 1200 g. For FACS analysis, cells were firstly stained with Live and Dead violet viability kit (Invitrogen, Cat# L34955) or Zombie Aqua fixable viability kit (BioLegend, Cat# 423102) and followed by nonspecific binding blocking antibody anti-CD16/32 (Thermo Fisher). Then cells were stained with other antibodies for surface molecule staining. For intracellular protein staining, cells were fixed and permeabilized with Foxp3 staining buffer kit (eBioscience, Cat# 00-5523-00). For flow cytometry staining, all antibodies were used at 1:200 final dilution unless otherwise described. The antibodies used in the study: Anti-mouse CD3ε-FITC (Tonbo Biosciences, Cat# 35-0031-U500), Anti-mouse CD3-eFluor710 (eBioscience, Cat# 46-0032), Anti-mouse CD4-APC-eFluor®780 (eBioscience, Cat#47-0041), Anti-human/mouse CD45RB-PE (eBioscience, Cat# 12-0455-83), Anti-mouse CD16/CD32-FITC (Tonbo Biosciences, Cat# 35-0161-U100), Anti-mouse CD45.1-APC-Cy7 (Tonbo Biosciences, Cat# 25-0453-U100), Anti-human/Mouse-RORγt-PE (eBioscience, Cat# 12-6988-82), Anti-mouse KLRG1-PerCP-eFluor 710 (eBioscience, Cat# 46-5893-82), Anti-mouse IL-13-Alexa Fluor 488 (eBioscience, Cat# 53-7133-82), Anti-human/mouse IL-22-APC (eBioscience, Cat# 17-7222-82), Anti-mouse CD45.1-PerCP-Cy5.5 (eBioscience, Cat# 45-0453-82), Anti-mouse CD45.2-PerCP-Cy5.5 (eBioscience, Cat# 45-0454-82), Anti-GATA3 (BD Biosciences, Cat# 558686), Anti-mouse/human IL-5-Brilliant Violet 421 (BioLegend, Cat# 504311), Anti-mouse Foxp3-eFluor 450 (eBioscience, Cat# 48-4875-82), Anti-mouse CD4-APC-Cy7 (Tonbo Biosciences, Cat# 25-0041-U100), Anti-mouse TCRβ-PerCP-Cy5.5 (Tonbo Biosciences, Cat# 65-5961-U100), Anti-mouse TCRβ-eFluor 450 (eBioscience, Cat# 48-5961-82), Anti-mouse CD8α-APC-Cy7 (BioLegend, Cat# 100714), Anti-human/mouse CD44-APC (eBioscience, Cat# 17-0441-83), Anti-mouse CD62L-PE-Cy7 (eBioscience, Cat# 25-0621-82), Anti-mouse IL-17A-PerCP-Cy5.5 (Invitrogen, Cat# 45-7177-82), Anti-mouse IFNγ-APC (eBioscience, Cat# 17-7311-82), Anti-DCLK1 (Abcam, Cat# ab31704, 1:500 dilution), Anti-Vγ2-FITC (Biolegend, Cat# 137704), Anti-cKit-PE-Cy7 (eBioscience, Cat# 25-1171-82), Anti-mouse Ki-67-PE-cy7 (BD Biosciences, Cat# 561283). Anti-Vγ4 antibody (Clone: 17D1, 1:100 dilution) was previously described^76^. For cytokine staining, unless otherwise noted, cells were stimulated with 50 ng/ml PMA and 500 ng/ml ionomycin in IMDM medium (supplemented with 5% FBS, 100 µg/ml penicillin/streptomycin, 2 mM l-glutamine) for 4 h and Brefeldin A (2 µg/ml) was added 2 h before cells were harvested. The gating strategy of different immune cell populations shown in Supplementary Fig. 2. BD FACSCantoII was used for FACS data collection. BD FACS Diva v8.0.2 was used to collect flow cytometry data. FlowJo software (version 10.2) was used for FACS data analysis. Sony sorter (SH800) was used for cell sorting.

### Quantitative PCR

For quantification of gene expression, total RNA was isolated by RNeasy Micro kit (Qiagen) and reversely transcribed by GoScript™ Reverse Transcription kit (Promega). For quantification of mitochondrial DNA content, total DNA was isolated with DNeasy Blood &Tissue Kit (Qiagen). Quantitative real-time PCR was performed on CFX connect Real-Time PCR Detection System (Bio-Rad). SYBR Green (Bio-Rad) and gene-specific primers were used for each gene. Data are presented as technical triplicates unless otherwise indicated in the legend. All primers used are listed in Supplementary Table 6.

### Cellular ATP measurement

Cellular ATP from sorted γδT1 or γδT17 cells (5000 cells per assay well) was extracted by adding 100 µl 0.5% trichloroacetic acid (Sigma, T6399). ATP measurement was performed with the ENLITEN® ATP Assay System Bioluminescence Detection Kit (Promega, FF2000). luminescence was detected using Synergy H1 microplate reader (BioTek).

### Fatty acid uptake test

For small intestine epithelial cell isolation, the small intestine was cut open into 0.5–1 cm piece and washed with ice-cold sterile PBS for three times. Tissues were transferred into sterile calcium and magnesium-free HBSS containing HEPES (20 mM), EDTA (10 mM), and 1% FBS and shaken on ice every 1–2 min for 20 min. The cells in the supernatant were collected and then enriched by centrifuging at 800*g* for 5 min at 4 °C. Cells were digested with 0.25% trypsin at 37 °C for 1 min into single-cell suspension and washed with ice-cold sterile PBS for further use. For fatty acid uptake test, small intestine epithelial cells were stained with Bodipy FL C16 (Thermo) in PBS for 5 min. Data were collected by flow cytometry (BDCantoII) or a confocal microscope (Zeiss 710 confocal microscope).

### Immunofluorescence staining and confocal microscope analysis

Small intestine tissues were fixed overnight with 4% PFA. After three times PBS wash at room temperature and 0.5% Triton-PBS permeabilization for 15 min, tissue swiss-rolls were embedded in OCT compound (Tissue-Tek). Frozen sections at 10 µm were put on the slides followed by antibody and 4′,6-diamidino-2-phenylindole (DAPI) staining. For the primary antibody staining, anti-CD3-PE (BD, Cat#553063, 1:200 dilution), anti-KLRG1-Alexa488 (Invitrogen, Cat#53-5893-82, 1:200 dilution), and anti-DCLK1 (Abcam, Cat# ab31704, 1:800 dilution) were used under 4 °C overnight. For DCLK1 secondary antibody staining, Anti-rabbit IgG-Alexa Fluor 488 (Thermal Fisher, Cat# A11008, 1:2000 dilution) was used at room temperature for 1 h. Data were collected by a confocal microscope (Zeiss 710 confocal microscope). ZEN 2011 v14.0.10.201 was used to collect confocal images.

### Hematoxylin and eosin (H&E) staining and PAS staining

Tissues for H&E staining and PAS staining were processed as previously described^22^. Briefly, tissues were fixed overnight in 4% PFA. After washing and embedding in paraffin, tissues were cut into slices and put on the slides followed by dewaxing and dehydration. Then, tissue slices were stained with H&E or periodic acid and Schiff reagent. Histological scoring was performed under the following criteria: villous architecture (normal = 0, widened due to laminar propria inflammatory infiltration = 1, blunt = 2); IELs (no = 0, rare (<5/villus) = 1, frequent = 2); crypt hyperplasia (no = 1, yes = 1); goblet cell hyperplasia (no = 1, yes = 1); muscularis propria hypertrophy (no = 1, yes = 1); acute inflammation (no = 0, yes = 1). Total scores were summarized and quantified.

### RNA-Seq analyses

For RNA-Seq analyses of γδT17 cells, 2 × 10^3^ sorted γδT17 cells from the spleen, small intestine, or large intestine of control or *Tfam*^*fl/fl*^*Rorc-cre* littermate mice were used. RNA was isolated by RNAeasy Micro Kit. cDNA generation was performed with SMART-Seq® HT Kit (Takara). Sequencing libraries were generated with Nextera® XT DNA Library Preparation Kit (Illumina). For RNA-Seq analyses of small intestine tissues, control or *Tfam*^*fl/fl*^*Rorc-cre* littermate mice were used. RNA was isolated by RNAeasy Mini Kit (Qiagen). Libraries were generated with NEBNext Ultra II RNA library Prep Kit (NEB). Libraries were sequenced on an Illumina HiSeq 2500 instrument to produce 50 bp single-end reads. FastQC (v0.11.5) was used to ensure high per-base sequence quality of reads. Sequenced reads were mapped and raw count values quantified with STAR (v2.7.4a)^77^ to the Mus musculus genome (GRCm38/mm10 assembly) and filtered for uniquely mapped reads. Quantitated mRNA expression levels, FPKM aligned reads, were calculated based on exon regions using RSEM (v1.2.25)^78^. Differentially expressed genes (max FPKM ≥ 1, fold change ≥1.5, *q* value ≤0.05) were identified by DESeq2 (v1.26.0) analysis^79^. Gene Set Enrichment Analysis (GSEA) was performed for GO and KEGG analysis (MSigDB v6.0). Log2 transformed FPKM values were used for PCA in R with the prcomp function and then visualized using the rgl package (v0.100.30). Euclidian distance comparison was calculated based on the distance between the individual spleen samples and the mean intestine centroid coordinate, and the distance between the mean small intestine centroid coordinate and the individual large intestine samples. Heatmaps were created using the R package pheatmap (v1.0.12).

### In vitro enteroid culture

Small intestine crypts were isolated from the small intestine of 4–8-week-old C57BL/6 mice and embedded in a 50% Matrigel (Corning 352231) solution in complete enteroid media consisting of 50 ng/ml recombinant mouse EGF (Peprotech 315-09), 50 ng/ml recombinant murine noggin (Peprotech 250-38), and 50 ng/ml recombinant mouse r-spondin (R&D Systems 7150-RS) as previously described^80^. Enteroids were passaged a minimum of one time prior to experimentation in 24-well plates at approximately 150 organoids per well. For whole mount immunofluorescent staining, samples were fixed in 4% paraformaldehyde for 30 min at room temperature, permeabilized with 0.1% Triton X-100 for 30 min at room temperature, then blocked in an ice-cold PBS solution containing 5% normal goat serum and 1% BSA for 60 min. Fixed organoids were then incubated overnight at 4 °C with DCLK1 antibody (Abcam, Cat# ab31704, 1:400 dilution) and followed by overnight at 4 °C with Alexa Fluor 488 goat anti-rabbit secondary antibodies (Thermal Fisher, Cat# A11008, 1:2000 dilution). Samples were subsequently counterstained with a 1:5000 DAPI solution (Fisher Scientific 62248) for 1 h at room temperature and stored at 4 °C until visualization using a confocal microscope (Zeiss 710 confocal microscope).

### Hydrodynamic gene delivery of IL-22 in vivo

IL-22 plasmid DNA was introduced into mice using a hydrodynamic tail vein injection-based gene transfer technique as previously described^34^. Briefly, plasmid DNA was diluted in TransIT-EE Hydrodynamic Delivery Solution (Mirus) (5 μg/ml) in a volume equal to 10% body weight (0.1 ml/g). The DNA solution was injected into mice through the tail vein using a 30-gauge needle within a time period of 5 s. Hydrodynamic injection was performed in mice at the age of 6 weeks old. Mice were sacrificed 4 weeks after the hydrodynamic injection. To ensure the success of delivery, the tissue RNA was prepared and monitored for IL-22 target gene (*Reg3g*) expression.

### Tissue metabolome analysis

Tissue samples were extracted with pre-normalization to the sample protein content of 500 μg/ml as previously described^81^. Global metabolomics profiling was performed on a Thermo Q-Exactive Oribtrap mass spectrometer coupled to a Dionex UHPLC and autosampler. Samples were analyzed in both positive and negative heated electrospray ionization with a mass resolution of 35,000. Separation was achieved by gradient elution on an ACE 18-pfp 100 × 2.1 mm, 2 µm column with a flow rate of 350 μl/min at 25 °C column temperature. Mobile phase A was 0.1% formic acid in water and mobile phase B was unmodified acetonitrile. Injection volume was 4 µL for negative ionization and 2 µL for positive ionization. Data from positive and negative ion modes were separately subjected to statistical analyses. All subsequent data analyses were normalized to the sum of metabolites for each sample. MZmine2 (freeware, v2.53) was used for alignment, gap filling, and metabolite identification^82^. All adducts and complexes were identified and removed from the data set.

## Quantification and statistical analysis

All data contain at least *n* = 3 per group and are shown as mean ± SD. Two-sided Student’s *t-*test was performed for statistical analysis by GraphPad Prism 7.04 software package in the manuscript unless otherwise noted. *P* values were indicated with asterisks (*P ≤ 0.05; **P ≤ 0.01; ***P ≤ 0.001; ****P ≤ 0.0001) in the figures. No multiple comparison was performed unless otherwise noted. PCA for metabolome was performed in Metaboanalyst 3.0 on the sum normalized, log2 transformed, and autoscaled data^83^.

### Reporting summary

Further information on research design is available in the Nature Research Reporting Summary linked to this article.

## Supplementary information

Supplementary Information

Reporting summary

## Data Availability

All data supporting the findings of this study are available within the article and its supplementary materials and from the corresponding author upon reasonable request. The RNA-sequencing data of splenic, small intestine and large intestine control and Tfam-deficient γδT17 cells, and small intestine tissues of control and *Tfam*^*fl/fl*^*Rorc-cre* mice generated in this study have been deposited in the NCBI database under accession code GSE152535. Source data are provided with this paper.

## Source data

Source Data
