# Supplementary material for: Mitochondrial transcription factor A in RORγt+ lymphocytes regulate small intestine homeostasis and metabolism
Source: Nat Commun. 2021 Jul 22;12:4462. doi: 10.1038/s41467-021-24755-9 (PMC8298438; doi:10.1038/s41467-021-24755-9)
Supplement: Supplementary file 1 — Supplementary Information [file 41467_2021_24755_MOESM1_ESM.pdf]

Supplementary information

Mitochondrial transcription factor A in RORγt⁺ lymphocytes regulate small intestine homeostasis and metabolism

Zheng Fu[1], Joseph W. Dean[1], Lifeng Xiong[1], Michael W. Dougherty[2], Kristen N. Oliff[1], Zong-ming E. Chen[3], Christian Jobin[1, 2], Timothy J. Garrett[4], and Liang Zhou[1 *]

[1] Department of Infectious Diseases and Immunology, College of Veterinary Medicine, University of Florida, Gainesville, FL 32608, USA
[2] Department of Medicine, University of Florida, Gainesville, FL, 32608 USA.
[3] Department of Laboratory Medicine and Pathology, Mayo Clinic, Rochester, MN 55905, USA
[4] Department of Pathology, Immunology and Laboratory Medicine, University of Florida, Gainesville, FL, 32608 USA.
* Correspondence: Liang Zhou; Tel: 352-294-8293; Fax: 352-392-9704; liangzhou497@ufl.edu

Supplementary figures

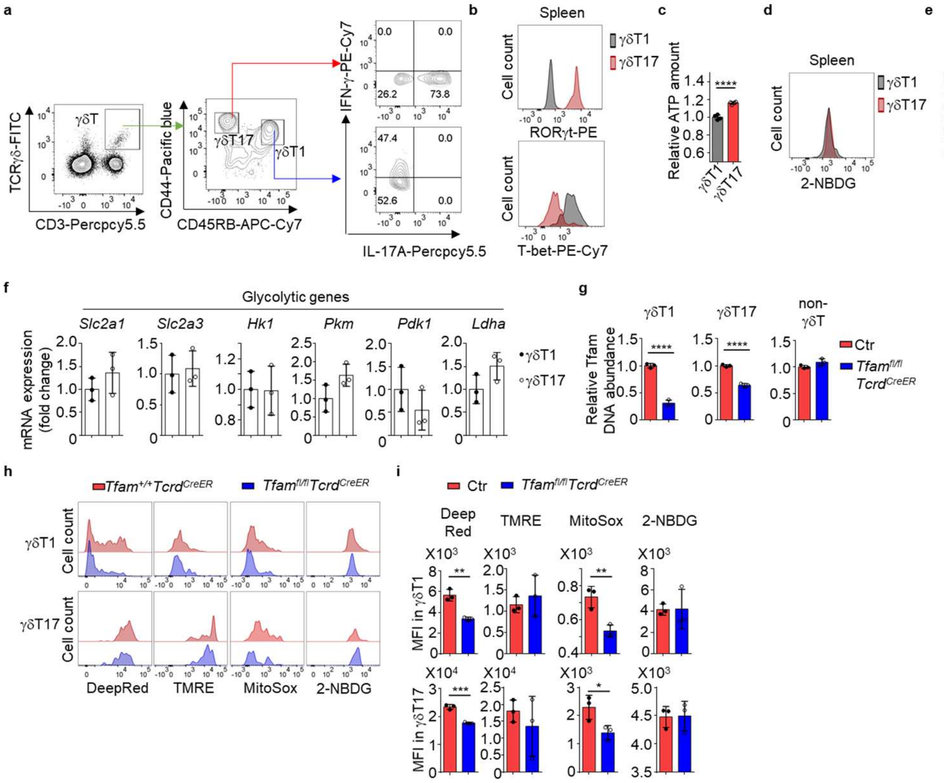

Supplementary Fig. 1 Tfam deficiency affects γδT1 and γδT17 cells differentially. **a**, Flow cytometry gating strategy of splenic γδT, γδT1 and γδT17 cells and IL-17A and IFN-γ expression in the splenic γδT1 and γδT17 cells. Representative data of three independent experiments. This gating strategy were also used for gating in Fig. 1. **b**, RORγt and T-bet expression in the splenic γδT1 and γδT17 cells by flow cytometry. Representative data of two independent experiments. **c**, Cellular ATP in splenic γδT1 cells and γδT17 cells of 3-week-old C57BL/6 mice. Each dot represented one replicate of cells sorted from pooled spleens of two 3-week-old C57BL/6 mice (n=4 for each group) (*****P*<0.0001). Representative data from three independent experiments. Data were normalized to equal cell number for each group. **d**, 2-NBDG uptake in the splenic γδT1 and γδT17 cells of C57BL/6 mice by flow cytometry. Representative data of three independent experiments. **e**, Mean fluorescence intensity (MFI) of 2-NBDG in **d** (n=3) (*P*=0.7809). Compiled data from one experiment. **f**, Expression of several key glycolytic genes in splenic γδT1 and γδT17 cells measured by qRT-PCR (n=3 biological repeats) (*Slc2a1*, *P* =0.2893; *Slc2a3*, *P* =0.7310; *Hk1*, *P* =0.9476; *Pkm*, *P* =0.0814; *Pdk1*, *P* =0.2929; *Ldha*, *P* =0.1115). Compiled data from two independent experiments. **g**, *Tfam* deletion efficiency at *Tfam* locus (exon 7) related to *Hbb* (encoding hemoglobin beta chain complex) locus in splenic γδT1 cells, γδT17 cells and non-γδT lymphocytes measured by genomic qPCR (n=3 for each group) (γδT1: *****P*<0.0001; γδT17: *****P*<0.0001; non-γδT: *P*=0.1030). Ctr or $Tfam^{fl/fl}Tcrd^{CreER}$ mice were treated with tamoxifen (2mg/mouse by IP injection) daily for 5 days. Data were collected 3 days after the last tamoxifen injection. Representative data of two independent experiments. **h**. MitoTracker-Deep Red, TMRE, MitoSox^{TM} Red and 2-NBDG staining in splenic γδT1 and γδT17 cells by flow cytometry. Representative data of two independent experiments. **i**, MitoTracker-Deep Red, TMRE, MitoSox^{TM} Red and 2-NBDG mean fluorescence intensity (MFI) (n=3) (γδT1: DeepRed, ***P* =0.0024; TMRE, *P* =0.5272; MitoSox, ***P* =0.0085; 2-NBDG, *P* =0.9688; γδT17: DeepRed,

***P =0.0004; TMRE, P =0.4551; MitoSox, *P =0.0348; 2-NBDG, P =0.9365). Compiled data from one experiment. Ctr included $Tfam^{+/+}Tcrd^{CreER}$ and $Tfam^{fl/+}Tcrd^{CreER}$ mice in **g** and **i**. Data are shown as mean ± SD in **c**, **e**-**g**, **i**.

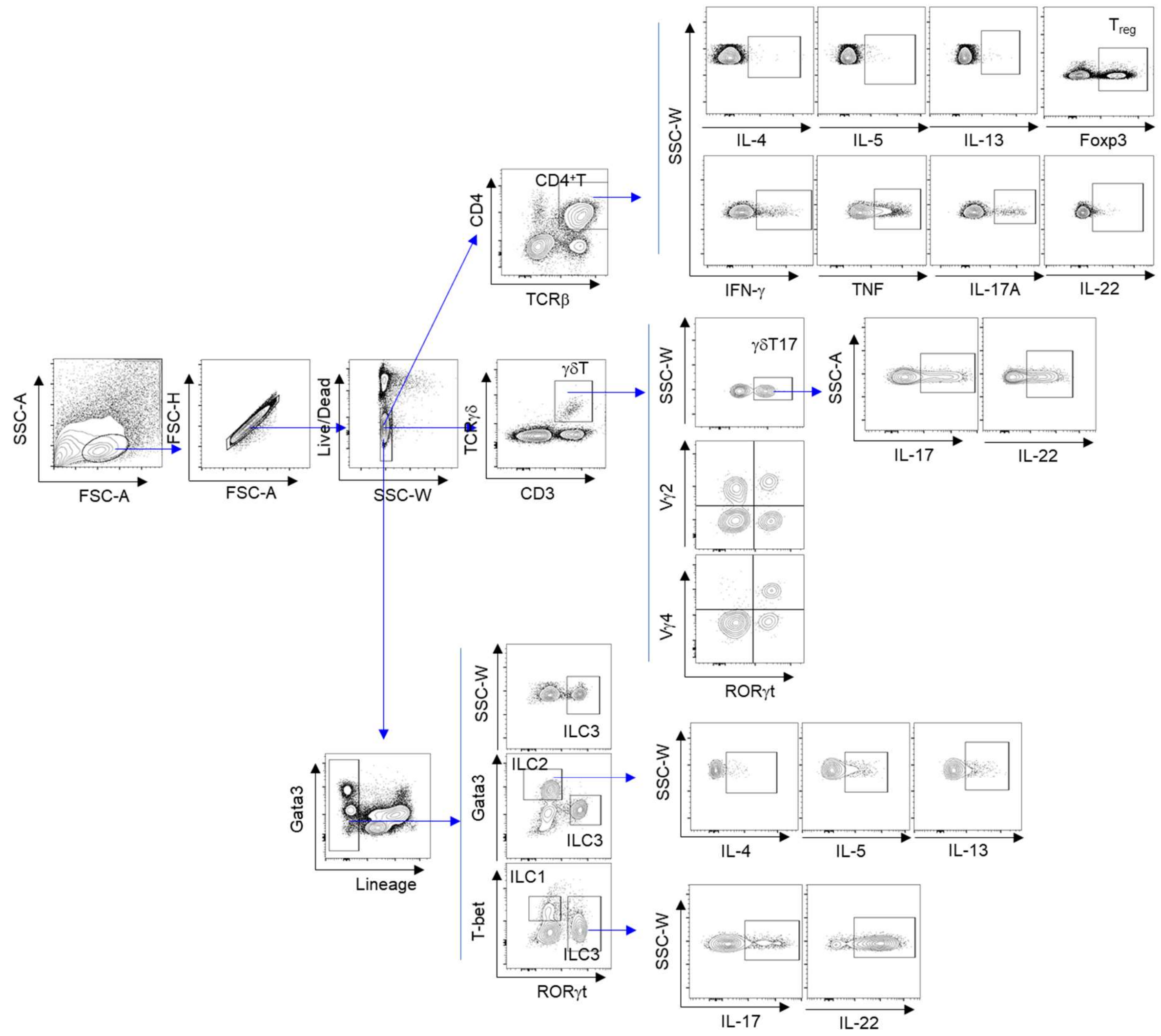

Supplementary Fig. 2 Gating strategies used for identification of different cell types. These gating strategies were used in main figures from Figure 2 to Figure 6 and Supplementary Figure 3 to 5.

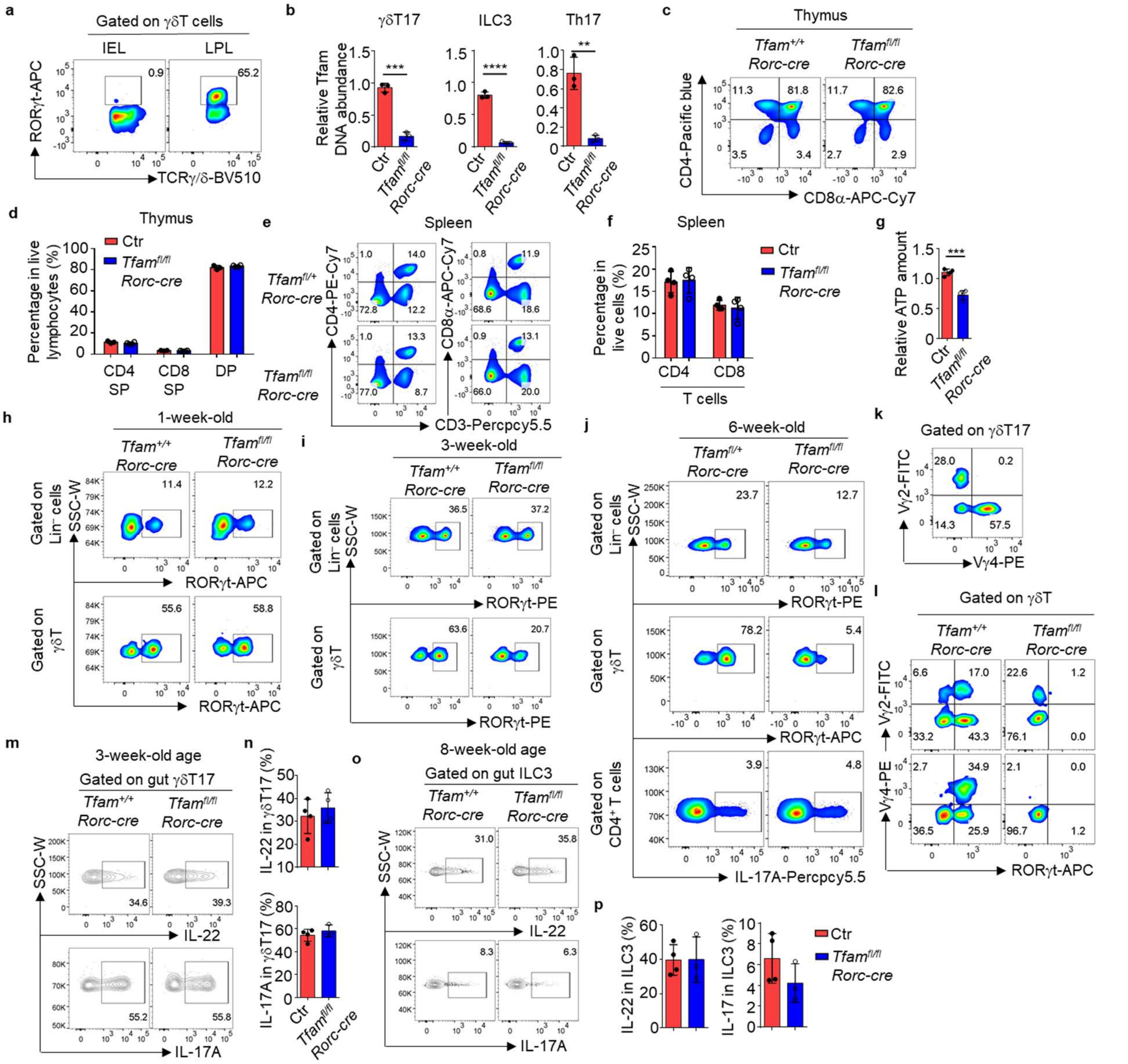

Supplementary Fig. 3 Tfam is critical for ILC3 and γδT17 cell maintenance in the gut. Ctr included *Tfam*$^{+/+}$, *Tfam*$^{fl/+}$, *Tfam*$^{fl/fl}$, *Tfam*$^{+/+}$*Rorc-cre* and *Tfam*$^{fl/+}$*Rorc-cre* mice. **a**, RORγt expression in γδT cells of the intraepithelial lymphocyte (IEL) and lamina propria lymphocytes (LPL) of the large intestine of C57BL/6 mice measured by flow cytometry. Gated on CD3$^+$TCRγ/δ$^+$ lymphocytes. Representative data of two independent experiments. **b**, *Tfam* deletion efficiency at *Tfam* locus (exon 7) related to *Hbb* in large intestinal γδT17 cells (left) (****P* =0.0002), ILC3s (middle) (*****P* <0.0001) and Th17 cells (right) (***P* =0.0024) (n=3 biological repeats). Ctr included *Tfam*$^{+/+}$*Rorc-cre* and *Tfam*$^{fl/+}$*Rorc-cre* mice. Representative data of two independent experiments. **c**, CD4 and CD8α expression in the thymus of mice with indicated genotypes assessed by flow cytometry. Representative data of two independent experiments. **d**, CD4$^+$CD8α$^-$ cells (CD4 SP) (*P* =0.2595), CD4$^-$CD8α$^+$ cells (CD8 SP) (*P* =0.4569) and CD4$^+$CD8α$^+$ cells (DP) percentages (*P* =0.2422) in total lymphocytes shown in **c** (Ctr, n=3, *Tfam*$^{fl/fl}$*Rorc-cre*, n=4). Compiled data from one experiment. **e**, CD4$^+$T cells and CD8$^+$T cells in the spleen of mice with indicated genotypes assessed by flow cytometry. Representative data of two independent experiments. **f**, CD4$^+$T cells and CD8$^+$T cells percentages in total lymphocytes (n=4) (CD4$^+$T: *P* =0.8378; CD8$^+$T: *P* =0.6332).

Compiled data from two independent experiments. **g**, Cellular ATP in splenic γδT17 cells of 3-week-old Ctr and *Tfam*$^{fl/fl}$*Rorc-cre* mice (***P =0.0002). Each dot represented one replicate of cells sorted from pooled spleens of two 3-week-old mice of indicated genotypes (n=4 for each group). Representative data from two independent experiments. Data were normalized to equal cell number for each group. **h**, ILC3s (upper panel, gated on Lin^{-} lymphocytes) and γδT17 cells (lower panel, gated on γδT cells) in the large intestine of 1-week-old mice with indicated genotypes measured by flow cytometry. Representative data of three independent experiments. Lineage (Lin) markers include CD3, CD5, CD19, Ly6G, CD11b, CD11c. **i**, ILC3s (upper panel, gated on Lin^{-} lymphocytes) and γδT17 cells (lower panel, gated on γδT cells) in the large intestine of 3-week-old mice with indicated genotypes measured by flow cytometry. Representative data of three independent experiments. **j**, ILC3s (upper panel, gated on Lin^{-} lymphocytes), γδT17 cells (middle panel, gated on γδT cells) and Th17 cells (lower panel, gated on CD4^{+}T cells) in the large intestine of 6-week-old mice with indicated genotypes measured by flow cytometry. Representative data of three independent experiments. **k**, T cell receptor (TCR) Vγ2 and Vγ4 expression on γδT17 cells in the large intestine LPL of 6-week-old wild-type mice measured by flow cytometry. Representative data of three independent experiments. **l**, TCR Vγ2, Vγ4 and RORγt expression on total γδT cells from large intestine LPL of 6-week-old mice with indicated genotypes measured by flow cytometry. Representative data of two independent experiments. **m**, Flow cytometry analyses of IL-22 and IL-17A expression by γδT17 cells in small intestine LPL of 3-week-old mice with indicated genotypes. **n**, Percentages of IL-22^{+} γδT17 and IL-17A^{+} γδT17 within total γδT17 cells shown in **m** (Ctr, n=4; *Tfam*$^{fl/fl}$*Rorc-cre,* n=4for IL-22^{+} γδT17, n=3 for IL-17A^{+} γδT17) (IL-22^{+}, P =0.8713; IL-17^{+}, P =0.7020). Compiled data from two independent experiments. **o**, Flow cytometry analyses of IL-22 and IL-17A expression in ILC3s from large intestine LPL of 8-week-old mice with indicated genotypes. **p**, Percentages of IL-22^{+} ILC3 and IL-17A^{+} ILC3s within total ILC3s shown in **o** (Ctr, n=4; *Tfam*$^{fl/fl}$*Rorc-cre,* n=3) (IL-22^{+}, P =0.5633; IL-17^{+}, P =0.2237). Compiled data from two independent experiments. Data are shown as mean ± SD in **b**, **d**, **f**, **g**, **n**, **p**.

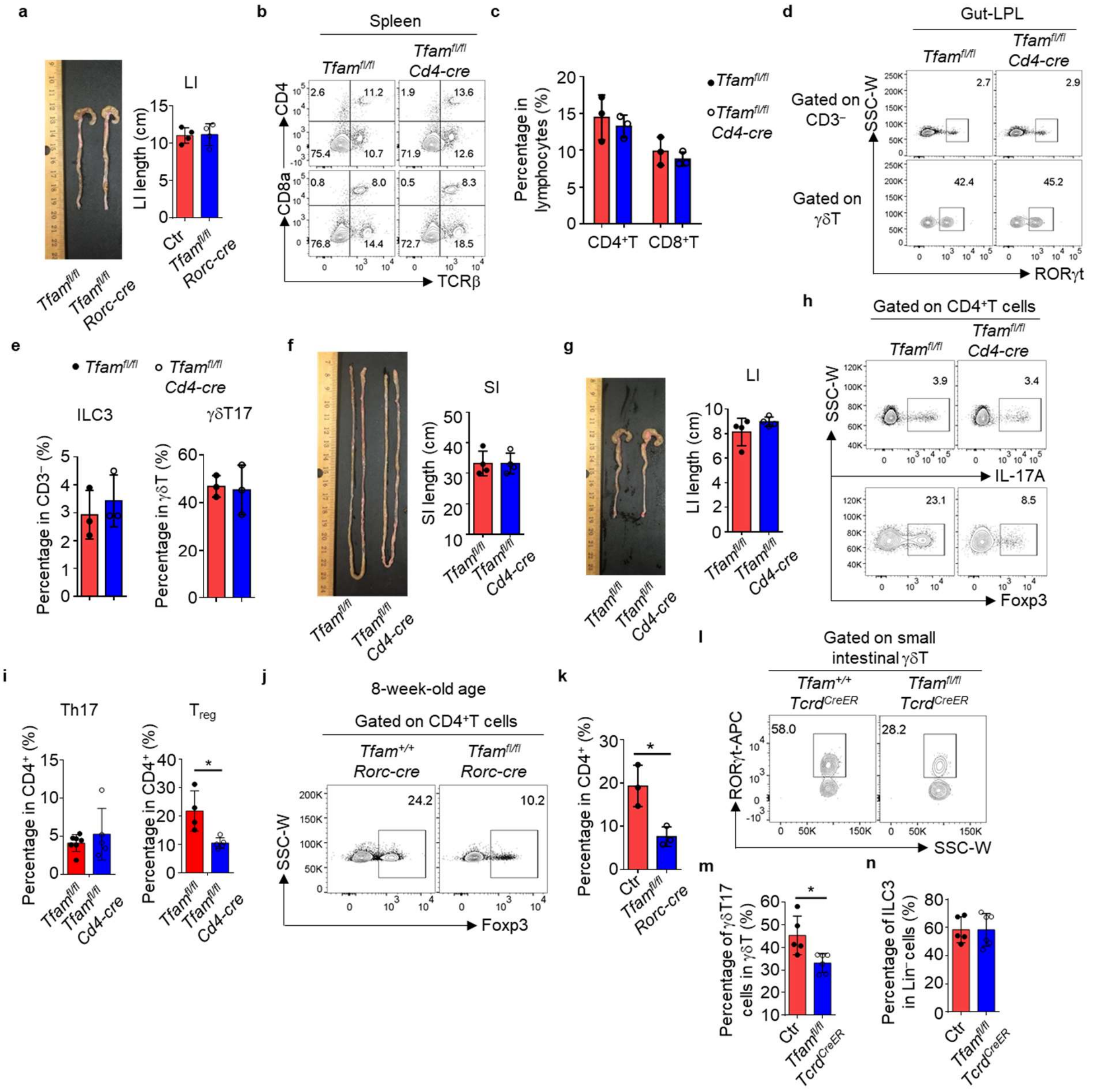

Supplementary Fig. 4 *Tfam* deficiency in γδT17 cells perturbs the small intestine tissue homeostasis. Ctr included *Tfam*$^{fl/+}$, *Tfam*$^{fl/fl}$, *Tfam*$^{+/+}$*Rorc-cre* and *Tfam*$^{fl/+}$*Rorc-cre* mice in **a** and **k**. **a**, Left panel, picture of the large intestine of 12-week-old control (Ctr) and *Tfam*$^{fl/fl}$*Rorc-cre* mice. Representative data of three independent experiments. Right panel, large intestine length in different ages of mice with indicated genotypes (n=4) (P=0.8933). Compiled data from two independent experiments. **b**, Flow cytometry analyses of TCRβ, CD4 and CD8α expression in splenic lymphocytes from 8-week-old mice with indicated genotypes. **c,** Percentages of CD4$^+$ and CD8$^+$ T cells within live lymphocytes shown in **b** (n=3 for each group) (CD4$^+$ T, P=0.1275; CD8$^+$ T, P=0.0874). Compiled data from two independent experiments. **d**, Flow cytometry analyses of RORγt expression in CD3$^-$ lymphocytes and total γδT cells from large intestine LPL of 8-week-old mice with indicated genotypes. **e**, Percentages of ILC3s in CD3$^-$ lymphocytes (P=0.5537) and γδT17 cells in total γδT cells (P=0.8267) shown in **d** (n=3 for each group). Compiled data from one experiment. **f**, Left panel, picture of the small intestine of 3-month-old littermate mice with indicated genotypes. Right panel, small intestine length in mice with indicated genotypes (n=4 for

each group) (P >0.9999). Compiled data from two independent experiments. **g**, Left panel, picture of the large intestine of 3-month-old littermate mice with indicated genotypes. Right panel, large intestine length in mice with indicated genotypes (n=4 for each group) (P =0.2134). Compiled data from two independent experiments. **h**, Flow cytometry analyses of IL-17A and Foxp3 expression by CD4$^+$ T cells in large intestinal LPL of 8-week-old mice of indicated genotypes. **i,** Percentages of Th17 cells (*Tfam*$^{fl/fl}$, n=7; *Tfam*$^{fl/fl}$*Cd4-cre,* n=5) and T_{reg} cells (*Tfam*$^{fl/fl}$, n=4; *Tfam*$^{fl/fl}$*Cd4-cre,* n=5) in CD4$^+$ T cells shown in **h** (Th17: P =0.4111; T_{reg} cells: *P =0.0103). Compiled data from two independent experiments. **j**, Flow cytometry analyses of Foxp3 expression by CD4$^+$ T cells in large intestine LPL of 8-week-old mice with indicated genotypes. **k**, Percentages of T_{reg} cells within CD4$^+$ T cells shown in **j** (n=3 for each group) (*P =0.0186). **l**, RORγt expression in small intestinal $\gamma\delta$T cells in mice with indicated genotypes measured by flow cytometry. Representative data of three independent experiments. Mice were treated with tamoxifen daily for 5 days. Data were collected three weeks after the last tamoxifen injection. **m**, RORγt$^+\gamma\delta$T (i.e., $\gamma\delta$T17) cell percentages within total small intestinal $\gamma\delta$T cells (Ctr, n=5; *Tfam*$^{fl/fl}$*Tcrd*^{CreER}, n=6) (*P =0.0284). Compiled data from three independent experiments. **n**, ILC3s in Lin$^-$ lymphocytes in the small intestine of mice with indicated genotypes (Ctr, n=5; *Tfam*$^{fl/fl}$*Tcrd*^{CreER}, n=6) (P =0.9941). Compiled data from three independent experiments. Ctr included *Tfam*$^{+/+}$*Tcrd*^{CreER} and *Tfam*$^{fl/+}$*Tcrd*^{CreER} mice in **m** and **n**. Data are shown as mean ± SD in **a**, **c**, **e-g**, **i**, **k**, **m**, **n.**

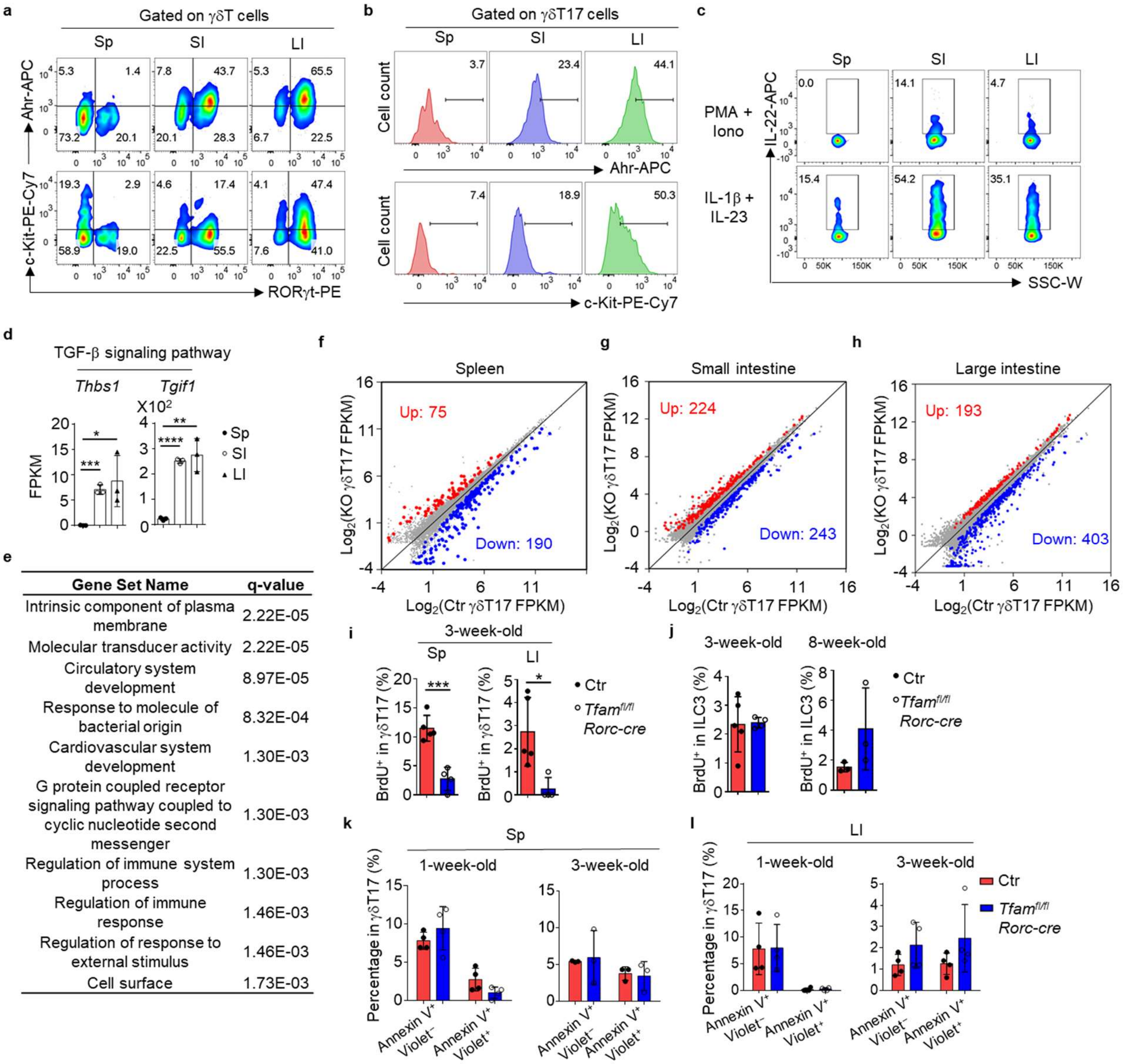

e

Gene Set Name	q-value
Intrinsic component of plasma membrane	2.22E-05
Molecular transducer activity	2.22E-05
Circulatory system development	8.97E-05
Response to molecule of bacterial origin	8.32E-04
Cardiovascular system development	1.30E-03
G protein coupled receptor signaling pathway coupled to cyclic nucleotide second messenger	1.30E-03
Regulation of immune system process	1.30E-03
Regulation of immune response	1.46E-03
Regulation of response to external stimulus	1.46E-03
Cell surface	1.73E-03

Supplementary Fig. 5 Tissue-specific transcriptomic signature of γδT17 cells. a, Expression of Ahr, c-Kit and RORγt by γδT cells in Sp, SI and LI of wild-type mice measured by flow cytometry. Representative data of two independent experiments. **b**, Ahr and c-Kit expression by γδT17 cells in Sp, SI and LI of wild-type mice measured by flow cytometry. Representative data of two independent experiments. **c**, Expression of IL-22 by γδT17 cells in Sp, SI and LI of wild-type mice measured by flow cytometry. Cells were stimulated with IL-1β (10ng/ml) and IL-23 (10ng/ml) for 6 hours. Representative data of one independent experiment. **d**. RNA-seq FPKM values of indicated gene expression by γδT17 cells in Sp, SI, and LI of wild-type mice (mean ± SD) (n=3) (Sp *vs.* SI: *Thbs1*, ****P* =0.0002, *Tgif1*, *****P* <0.0001; Sp *vs.* LI: *Thbs1*, **P* =0.0398, *Tgif1*, ***P* =0.0026). **e**, Gene Ontology (GO) analysis of the 50 splenic TSGs shown in **Fig. 4b**. **f-h**, Differential gene expression by RNA-seq analysis of splenic (**f**), small intestinal (**g**) or large intestinal (**h**) control (Ctr) and Tfam-deficient (KO) γδT17 cells (fold change ≥ 1.5, q value ≤ 0.05) (n=3). Each sample was sorted from an individual mouse. **i**, Percentages of BrdU+ γδT17 cells within total splenic and large intestinal γδT17 cells from 3-week-old mice by flow cytometry analysis (Ctr, n=5; *Tfam$^{fl/fl}$Rorc-cre*, n=4) (Sp, ****P* =0.0005; LI, **P* =0.0159). Compiled data from

two independent experiments. **j**, Percentages of BrdU$^+$ ILC3s within total large intestinal ILC3s from mice of indicated ages by flow cytometry analysis (3-week-old: Ctr, n=5; *Tfam*$^{fl/fl}$*Rorc-cre*, n=4; 8-week-old: Ctr, n=3; *Tfam*$^{fl/fl}$*Rorc-cre*, n=3) (3-week-old, P=0.7101; 8-week-old, P=0.3182). Compiled data from two independent experiments. **k-l**, Percentages of Annexin V$^+$Violet$^-$ and Annexin V$^+$Violet$^+$ cells in Sp (**i**) and LI (**j**) $\gamma\delta$T17 cells of 1-week-old and 3-week-old mice with indicated genotypes (n=4) measured by flow cytometry (Sp: 1-week-old, Annexin V$^+$Violet$^-$, P=0.3270, Annexin V$^+$Violet$^+$, P=0.0832; 3-week-old, Annexin V$^+$Violet$^-$, P=0.5499, Annexin V$^+$Violet$^+$, P=0.4648; LI: 1-week-old, Annexin V$^+$Violet$^-$, P=0.9592, Annexin V$^+$Violet$^+$, P=0.7199; 3-week-old, Annexin V$^+$Violet$^-$, P=0.1693, Annexin V$^+$Violet$^+$, P=0.1977). Compiled data from one experiment. Ctr included *Tfam*$^{+/+}$, *Tfam*$^{fl/+}$, *Tfam*$^{fl/fl}$, *Tfam*$^{+/+}$*Rorc-cre* and *Tfam*$^{fl/+}$*Rorc-cre* mice in **f-l**. KO represented *Tfam*$^{fl/fl}$*Rorc-cre* in **f-h**. Data are shown as mean ± SD in **d**, **i-l.**

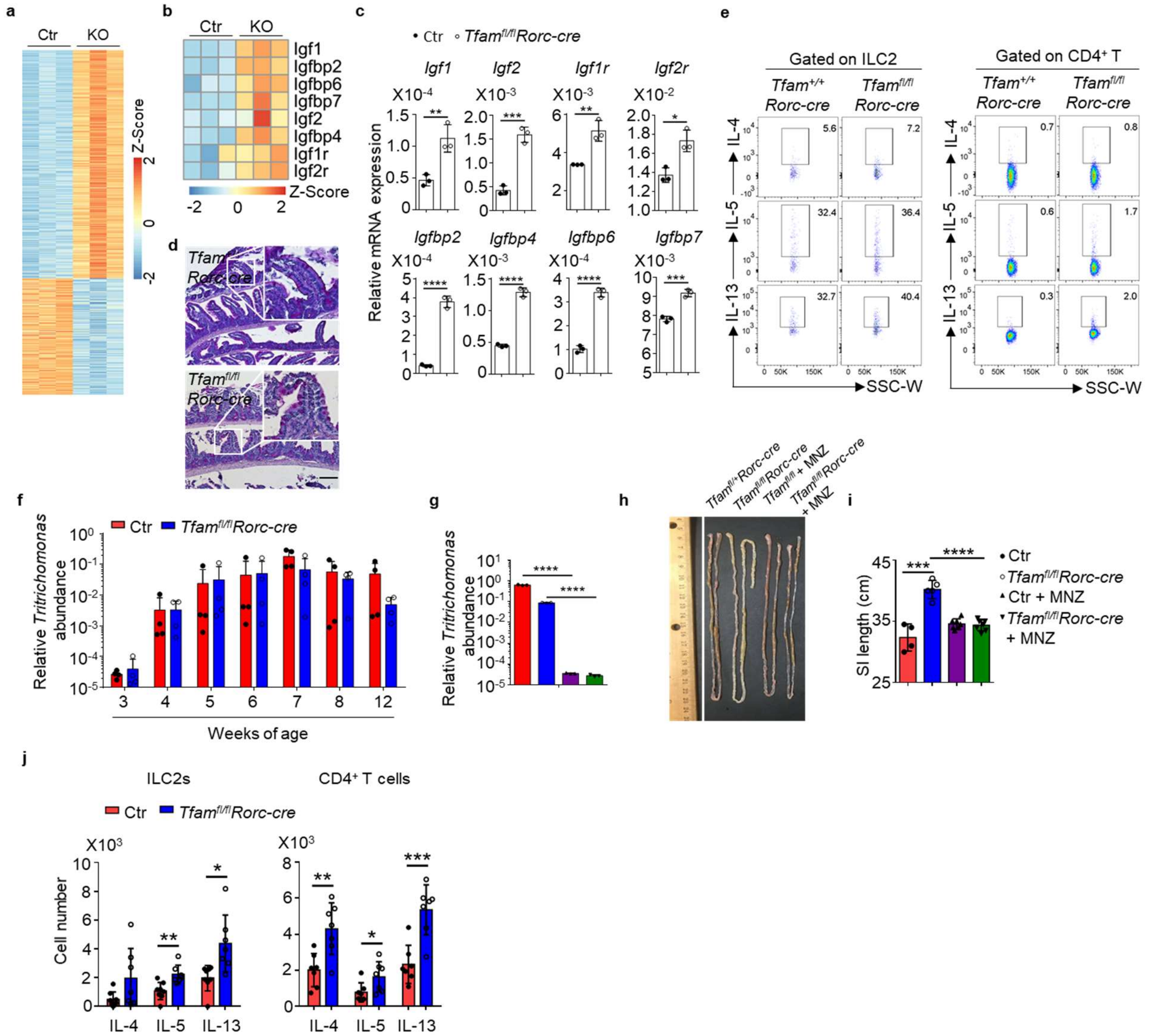

Supplementary Fig. 6 Enhanced tuft cell-type 2 immune circuit in the small intestine of *Tfam*$^{fl/fl}$*Rorc-cre* mice. Ctr included *Tfam*$^{+/+}$*Rorc-cre* and *Tfam*$^{fl/+}$*Rorc-cre* mice. KO indicated *Tfam*$^{fl/fl}$*Rorc-cre* mice in **a**-**c**. **a**, Heatmap of the 1838 differentially regulated genes from the RNA-seq analysis of small intestine tissues of Ctr and KO (n=3 for each group). Genes were ranked in a descending order based on the fold changes of expression (KO/Ctr). **b**, Heatmap of genes related to insulin growth factor (IGF) signaling by RNA-seq analysis of small intestinal tissues of Ctr and KO mice (n=3). Genes were ranked in a descending order based on the fold changes of expression (KO/Ctr). **c**, qRT-PCR of indicated genes in small intestine tissues of 3-month-old mice with indicated genotypes (n=3 for each group) (*Igf1*, ***P* =0.0083; *Igf2*, ****P* =0.0004; *Igf1r*, ***P* =0.0047; *Igf2r*, **P* =0.0105; *Igfbp2*, *****P* <0.0001; *Igfbp4*, *****P* <0.0001; *Igfbp6*, *****P* <0.0001; *Igfbp7*, ****P* =0.0006). Compiled data from one independent experiments. **d**, PAS staining of small intestinal tissue sections of 12-week-old *Tfam*$^{+/+}$*Rorc-cre* and *Tfam*$^{fl/fl}$*Rorc-cre* mice. Representative data of three mice in each group. Scale bar, 200 μm. **e,** Flow cytometry analysis of IL-4, IL-5 and IL-13 expression by ILC2s (gated on Lineage^{-}Gata3^{+}) and CD4^{+} T cells (gated on CD3^{+} CD4^{+}) in small intestine draining lymph nodes (siLN) of 3-month-old mice with indicated genotypes. Representative data of two independent experiments. Lineage markers include CD3, CD5, CD19, Ly6G, CD11b, CD11c. **f**, q-PCR of *Tritrichomonas* abundance in feces of the mice with indicated genotypes at different ages (n=4 for each group) (week3, *P* =0.5919;

week4, P=0.9738; week5, P=0.8352; week6, P=0.9168; week7, P=0.1559; week8, P=0.5413; week12, P=0.1898). Ctr included *Tfam*$^{+/+}$, *Tfam*$^{fl/+}$, *Tfam*$^{fl/fl}$, *Tfam*$^{+/+}$*Rorc-cre* and *Tfam*$^{fl/+}$*Rorc-cre* mice in **f**, **g**, **i** and **j**. **g**, *Tritrichomonas* abundance in feces of the mice treated with or without metronidazole (MNZ) (n=3) (Ctr *vs.* Ctr + MNZ: ****P<0.0001; *Tfam*$^{fl/fl}$*Rorc-cre vs. Tfam*$^{fl/fl}$*Rorc-cre* + MNZ: ****P<0.0001). Representative data from two independent experiments, and shown as triplicates of q-PCR. **h**, Picture of the small intestine of mice treated with or without MNZ shown in **g**. **i**, Small intestine length in mice with indicated genotypes shown in **h** (Ctr, n=4; *Tfam*$^{fl/fl}$*Rorc-cre*, n=5; Ctr + MNZ, n=7; *Tfam*$^{fl/fl}$*Rorc-cre* + MNZ, n=7) (Ctr *vs. Tfam*$^{fl/fl}$*Rorc-cre*, ***P=0.0004; *Tfam*$^{fl/fl}$*Rorc-cre vs. Tfam*$^{fl/fl}$*Rorc-cre* + MNZ, ****P<0.0001). Compiled data from two independent experiments. **j**, IL-4, IL-5 and IL-13 expression by ILC2s and $CD4^+$ T cells in small intestine draining lymph nodes (siLN) of 12-week-old Ctr and *Tfam*$^{fl/fl}$*Rorc-cre* mice infected with *Hpb* (n=7 for each group) (ILC2: IL-4, P=0.1098; IL-5, **P=0.0048; IL-13, *P=0.0163; $CD4^+$ T: IL-4, **P=0.0041; IL-5, *P=0.0392; IL-13, ***P=0.0006). Data were collected 14 days after the infection. Compiled data from three independent experiments. Data are shown as mean ± SD in **c**, **f**, **g**, **i**, **j**.

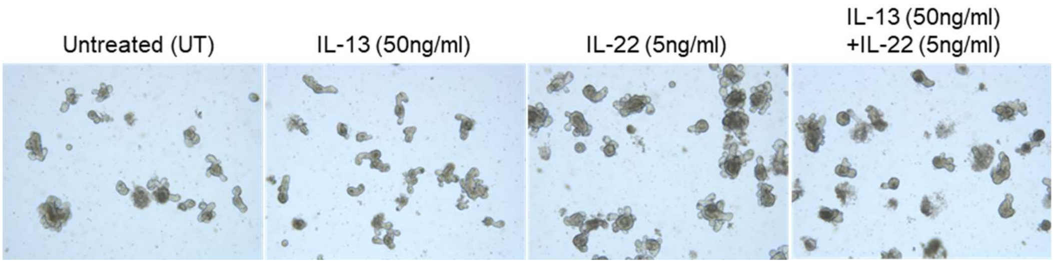

Supplementary Fig. 7 IL-22 suppresses IL-13-induced tuft cell differentiation. Enteroid culture with or without treatment of indicated cytokines for 2 days. Representative data of three independent experiments.

a

Gene Set Name (GO)	q-value
Response to oxygen containing compound	2.03E-64
Biological adhesion	1.86E-61
Small molecule metabolic process	1.52E-58
Transmembrane transport	3.93E-54
Defense response	6.18E-52
Homeostatic process	6.65E-50
Response to cytokine	4.33E-48
Regulation of immune system process	7.59E-45
Inflammatory response	1.03E-44
Lipid metabolic process	1.70E-41
Organic acid metabolic process	2.06E-41
Response to lipid	3.91E-39
Regulation of cell population proliferation	3.32E-37
Cellular response to endogenous stimulus	6.98E-37
Negative regulation of multicellular organismal process	1.32E-36
Cell-cell signaling	4.63E-36
Regulation of anatomical structure morphogenesis	3.30E-34
Animal organ morphogenesis	5.20E-34

b

Description (KEGG)	q-value
Hematopoietic cell lineage	6.92E-18
ECM-receptor interaction	1.29E-17
Focal adhesion	3.59E-16
Arginine and proline metabolism	1.69E-14
Vascular smooth muscle contraction	9.69E-14
Cytokine-cytokine receptor interaction	4.24E-13
Chemokine signaling pathway	8.44E-13
Arachidonic acid metabolism	7.94E-12
Viral myocarditis	4.05E-11
Cell adhesion molecules (CAMs)	1.21E-10
Leukocyte transendothelial migration	1.37E-10
Type I diabetes mellitus	3.19E-10
Graft-versus-host disease	1.66E-09
Drug metabolism - cytochrome P450	3.22E-09
Leishmania infection	3.22E-09
Allograft rejection	3.33E-09
PPAR signaling pathway	1.03E-08
Calcium signaling pathway	1.58E-08
Complement and coagulation cascades	6.91E-08
Glycolysis / Gluconeogenesis	8.37E-08

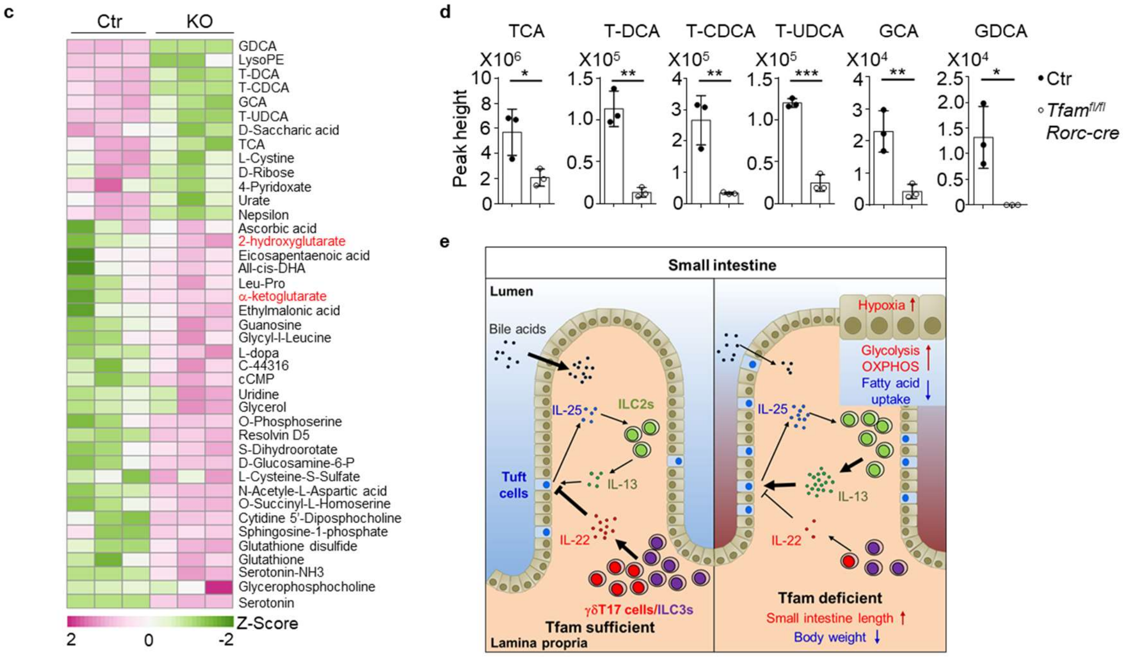

Supplementary Fig. 8 *Tfam* deletion in RORγt⁺ lymphocytes leads to global metabolomic changes in the small intestine. Ctr included *Tfam*$^{+/+}$*Rorc-cre* and *Tfam*$^{fl/+}$*Rorc-cre* mice. KO indicated *Tfam*$^{fl/fl}$*Rorc-cre* mice. **a-b**, GO analysis (**a**) or KEGG analysis (**b**) of the 1838 DEGs in RNA-seq data of the small intestinal tissues as shown in **Fig. 5a**. **c,** Heatmap of differentially regulated metabolites between the small intestinal tissues of Ctr and *Tfam*$^{fl/fl}$*Rorc-cre* groups (n=3). Metabolites were ranked in a descending order based on the fold changes of abundance (Ctr/KO). **d**, Peak height values of differentially regulated bile acids in the small intestinal tissues identified by the metabolome analysis (n=3) (mean ± SD) (TCA, **P* =0.0332; T-DCA, ***P* =0.0014; T-CDCA, ***P* =0.0069; T-UDCA, ****P* =0.0001; GCA, ***P* =0.0089; GDCA, **P* =0.0192). **e,** Working model of Tfam-mediated mitochondrial metabolism in regulation of γδT17 cell and ILC3 maintenance, as well as small intestine tissue remodeling and immunity.

Supplementary Tables

Supplementary Table 1. Tissue specific genes (TSGs) in the splenic (Sp), small (SI) and large intestinal (LI) γδT17 cells.

Category	Genes
Sp TSGs (50)	*Adgre4, Apoc1, Arl4c, Cadm1, Ccr10, Ccr3, Cd5l, Cfp, Cmbl, Cyp2s1, E2f2, Ear2, Epb4.1l3, Fcna, Fstl4, Fut7, Fxyd4, Gfra2, Ggt1, Gpr146, Gypa, Hmox1, Hpgd, Il18, Itgad, Lrrc75b, Mal, Mertk, Mrap, Nccrp1, Nhsl2, Nr1h3, Pdlim4, Popdc2, Rom1, S1pr1, Sbk1, Sema4a, Serp2, Slc22a4, Slc40a1, Snx22, Spic, Stmn2, Tbxa2r, Tlr1, Tmem51, Tppp3, Treml4, Vcam1, Vipr1*
Intestinal TSGs (113)	*2010300C02Rik, 4930503L19Rik, Abcb1a, Acta2, Actg2, Ahrr, Areg, Asb2, Atf3, B3galt5, Bambi, Bgn, Bhlhe40, Ccdc162, Ccl3, Ccl4, Ccl8, Ccno, Ccrl2, Cd160, Cd200r4, Cd93, Cdkn1a, Col1a1, Col1a2, Col3a1, Col6a1, Col6a2, Crem, Ctla4, Cxcl10, Cxcl2, Cxcl3, Cyp2j6, Dusp4, Dusp6, Eaf2, Egr1, Eln, Emid1, Fam167a, Fbln1, Fhl2, Fosb, Fosl2, Gadd45b, Gem, Gimap7, Gjb2, Gm5616, Gpnmb, Gstm5, Hbegf, Hic1, Hilpda, Hist1h1d, Hs3st1, Hspa1a, Hspa1b, Il17a, Il1r2, Il1rn, Il22, Itih5, Jchain, Jun, Kit, Lilr4b, Lrrn2, Ltbp4, Lum, Maff, Mfap4, Mgp, Mmp2, Mmp23, Myl10, Myl9, Mzb1, Neurl3, Nfkbia, Nfkbid, Nfkbiz, Nr4a1, Nr4a2, Nr4a3, Nrgn, Osgin1, P2rx7, Pdcd1lg2, Pdgfrb, Phlda1, Plk2, Ppp1r3b, Pram1, Ptgs2, Rarres2, Rgs1, Serpinb9, Serping1, Serpinh1, Sik1, Skil, Sparc, Spry1, Tbx21, Tgif1, Thbs1, Tnfaip3, Tnfsf11, Tpm2, Ucp3, Vps37b*
SI TSGs (12)	*Agr2, Art2b, Clu, Flt4, Gkn3, Grtp1, Il17f, Mmp9, Ogdhl, Pcp4, Ret, Tff2*
LI TSGs (7)	*Adgrg1, Agt, Ctgf, Gm9780, Klrb1c, Postn, Serpina3n*

Supplementary Table 2. Differentially expressed genes (DEGs) between splenic control and Tfam-deficient γδT17 cells.

Category	Genes (Tfam-deficient versus control)
Sp upregulated (75)	*Abcb1a, Abcb9, Acadsb, Acss2, Ajuba, Als2, Apol7e, Aqp9, Art2b, Atp8a2, Btla, Ccr7, Cd160, Cd27, Cd6, Chchd6, Ctsw, Dpp4, Enpp5, Ets2, Fam101b, Fam189b, Gimap4, Gimap7, Gm20696, Gramd3, Ift80, Il12rb2, Inpp4b, Izumo1r, Lancl1, Lck, Ldlrap1, Mboat1, Mctp2, Metrnl, Myc, Nipal1, Nop2, Nsg2, P2rx7, Padi2, Papss2, Pcsk1, Prf1, Prkch, Ptger4, Rab6b, Rapgef3, Rrp1b, Rundc3b, Scml4, Sell, Serpina3f, Sh2d1a, Slamf7, Slc14a1, Slc2a1, Slc9a2, Smyd3, Tcf7, Tgfb3, Themis, Tlr12, Tnfrsf23, Tnfrsf26, Tsc22d1, Ubash3a, Vav3, Wdr24, Wdr41, Wee1, Xcl1, Zbtb20, Zfp683*
Sp downregulated (190)	*1500009L16Rik, 6330408A02Rik, 9430020K01Rik, Abi3bp, Ablim3, Acpp, Acsbg1, Adam12, Adam8, Adrbk2, Agpat4, Agpat9, Amica1, Anxa1, Anxa2, Ap1s2, Aqp3, Arap3, Arnt2, Arrb1, Atg7, Atrnl1, B3gnt5, Bcl2a1a, Bsn, Capg, Car5b, Carhsp1, Casp1, Ccdc50, Ccnb2, Cd163l1, Cd48, Cdc14b, Cdc25b, Cenpa, Cers4, Chst10, Cib2, Cldnd1, Clnk, Cmah, Cnksr1, Cnot6, Cpe, Cpm, Crmp1, Cryba4, Cyp4f16, Daam1, Dap, Ddx28, Dennd5a, Dnah8, Dusp14, Dusp3, E2f2, Elf3, Endod1, Erbb2, Fah, Fam109b, Fam129b, Fam179a, Fam89a, Fgl2, Flnb, Fut7, Gcnt1, Gdpd5, Ggh,*

	Glipr1, Gm9961, Gng2, Gpr160, Hip1r, Hopx, Hs6st2, Igsf9b, Irf4, Itga4, Itga5, Itgb1, Itgb4, Itpr3, Kcnk1, Klf10, Klf12, Klf8, Klk13, Klrb1b, L1cam, Lgals1, Lmnb1, Lpcat1, Ltb4r1, Mal, Man1c1, Map6, Mapk6, Msc, mt-Co1, mt-Cytb, mt-Nd1, mt-Nd2, mt-Nd4, mt-Nd5, mt-Nd6, Myo1e, Myo1f, Naga, Nbeal2, Nccrp1, Ndst1, Nid1, Nipal3, Npnt, Nqo2, Nrp1, Nsmaf, Nxnl2, Osbpl3, Osbpl6, Pde1c, Pepd, Phf11b, Pik3cg, Plcb4, Plcd1, Plekho2, Plxdc1, Popdc2, Ppap2c, Ppp2cb, Prex1, Prr29, Prr5l, Psd2, Ptpn4, Pycard, Rap1gap2, Rln3, Rnf43, Rnpep, Rom1, Runx2, Ryk, S100a10, S100a4, Scpep1, Sdcbp2, Sec11c, Selm, Sepp1, Serinc2, Sgk1, Slc15a3, Slc1a5, Slc22a15, Slc52a3, Slc6a13, Socs2, Spcs3, Spink2, Spn, Spon2, St6galnac6, Steap3, Stmn2, Stx11, Sun1, Syt11, Tbc1d2, Tdrd9, Tfam, Thbd, Ticam1, Tmem171, Tmem37, Tmem50b, Tns4, Tppp3, Tspan6, Tubb3, Txndc5, Upp1, Vill, Wdfy2, Zbtb16, Zbtb32

Supplementary Table 3. DEGs between small intestinal control and Tfam-deficient γδT17 cells.

Category	Genes (Tfam-deficient versus control)
SI upregulated (224)	*1190002N15Rik, 1500012F01Rik, 1700025G04Rik, 5830411N06Rik, Abtb2, Acer2, Actn1, Acvr2a, Adam19, Ado, Alcam, Aldh2, Als2, Amz1, Angptl6, Anxa4, Arl4c, Arrdc3, Asns, Atf5, Atxn1, Aurkb, Bag1, Basp1, Bbc3, Bcl6, Bend5, Btla, C3, Ccnb2, Ccr7, Cd200, Cd27, Cd274, Cd9, Cfl2, Chdh, Ciart, Cited4, Cks1b, Clic4, Clybl, Coa7, Cox6a2, Cxcl1, Cyp2s1, Cyp51, Dapk2, Ddit3, Dzip1, Eef1g, Eif4ebp1, Eif5, Eif5a2, Emilin1, Faf1, Fam129c, Fam58b, Fam92a, Fas, Fgr, Gab2, Galm, Gbp2, Gbp3, Gbp5, Gbp6, Gbp8, Ggt5, Gja1, Gla, Glrx, Gm2a, Gpr146, Gucd1, Gzmb, H2-Ob, Hcar2, Hells, Hist1h1b, Hist1h1d, Hlf, Hpgds, Hs3st1, Hspa2, Hspa8, Hspe1, Hvcn1, Id3, Idh2, Ifngr2, Igfbp4, Iigp1, Il12rb2, Il17rb, Il1r2, Il21r, Il4, Izumo1r, Kcnn4, Kif23, Klf2, Klf3, Kntc1, Lgals3, Lilrb4a, Mboat1, Mcm3, Mcm8, Metrnl, Mettl13, Mettl22, Mfsd6, Mki67, Mmgt1, Mrpl30, Msmo1, Mta1, Mthfd1l, Mthfd2, Mturn, N4bp2, Nab2, Nabp2, Nampt, Nck2, Ndc80, Nipal1, Nqo1, Nupr1, Oas3, P4ha2, Parp11, Parp3, Pbx2, Pcsk1, Pdlim1, Pecr, Phgdh, Phka2, Pik3ip1, Pira2, Pkmyt1, Plac8, Plaur, Plscr3, Plxdc2, Pmepa1, Ppp3ca, Prkch, Ptger2, Ptger4, Ptgir, Ptms, Pus7, Pygl, Rasal1, Rasl11a, Rcan3, Rgs12, Rhbdd3, Rhob, Rpl13, Rpl22l1, Rpl32, Rpl4, Rps19, Rps7, Rras2, Sars, Satb1, Scarb1, Senp2, Sepw1, Shmt2, Slc16a1, Slc1a4, Slc35g1, Slc6a9, Slc7a3, Slc7a5, Ssbp2, Stat1, Stom, Taf10, Tapt1, Tbc1d4, Tcf7, Tef, Tesc, Tfdp1, Tgfb3, Tgfbr3, Tgm2, Thbs1, Themis, Thra, Tmem107, Tmem97, Tnfrsf23, Tnfrsf26, Tnfrsf4, Top2a, Trat1, Trib2, Trib3, Trim59, Tsc22d1, Tspan31, Ttc3, Tuba8, Ubash3a, Ubc, Ugcg, Uhrf1, Usp18, Wdr86, Xaf1, Xdh, Ybx1, Zcwpw1, Zdhhc8, Zfp281, Zfp524*
SI downregulated (243)	*2010300C02Rik, 5430421N21Rik, A830080D01Rik, Ablim3, Acot9, Acpp, Actrt3, Adam12, Ahrr, Aifm1, Aifm2, Akr1c12, Ang, Anks3, Arap3, Areg, Arg1, Arrdc4, Art2b, B3galt5, Bbs4, Bcl2a1a, Bcl2a1c, Bcl2a1d, Bhlhe40, Bmp4, Bspry, Btbd10, Cap2, Capg, Ccbl2, Ccdc125, Ccdc137, Ccl20, Ccnl1, Ccr2, Cd163l1, Cd69, Cdc14a, Cdh10, Cdk14, Cep44, Cers4, Chad, Clnk, Clstn3, Cnksr3, Comt, Cpe, Crmp1, Csf2, Csn3, Cxcl13, Cxcr3, Cxcr6, Cyp2j6, Cyp4f16, Daam1, Ddx25, Derl3, Dkk3, Dlg5, Dmrta1, Dnajb2, Dnajb5, Dnajc12, Dusp4, Dusp6, Enc1, Eps8l3, Ern1, Exog, F2r, Fam110a, Fasl, Fastkd3, Fhl2, Frmd5, Gadd45b, Gch1, Gdpd5, Gem, Gimap4, Gpatch2, Gpd2, Hbegf, Hexim1, Hjurp, Hlcs, Hs6st2, Hsd11b1, Icam1, Icam2, Igf1r,*

	Igsf5, Ikzf2, Ikzf3, Il1r1, Il2ra, Impg1, Irf5, Itga3, Itga5, Itih5, Jmjd6, Kcnk1, Kctd5, Klrb1, Klrk1, Lats2, Lax1, Ldhd, Leng9, Llgl2, Lrrc49, Lrrn2, Lsr, Ltb4r1, Ly6g5b, Maff, Magi1, Man1c1, Map3k14, March3, Marveld2, Mex3c, Mgat4a, Mitf, Mmp23, Morn3, Mrc2, Mtcl1, mt-Co1, mt-Cytb, Mtmr7, mt-Nd1, mt-Nd2, mt-Nd3, mt-Nd4, mt-Nd5, mt-Nd6, Myd88, N4bp3, Ncoa7, Nedd4l, Nfatc2, Nfkbia, Nfkbiz, Nid1, Nif3l1, Npas2, Npnt, Nrgn, Nxnl2, Ogdhl, Pde11a, Pde1c, Pde2a, Pdzd2, Pik3ap1, Plcb4, Plcd1, Plekhg1, Plekhm3, Pnisr, Pnpla6, Podnl1, Ppp1r10, Ppt2, Pqlc1, Prss12, Psd2, Ptgs2, Ptpn6, Ptprz1, Rai14, Rbpms2, Rcn2, Rffl, Rgs1, Rln3, Rnf43, Rrnad1, Runx2, Scg5, Sdc4, Sec16b, Sema6a, Serinc2, Serpinb6b, Serpinb9, Sertad1, Sla, Sla2, Slc16a6, Slc23a2, Slc27a6, Slc41a3, Slc7a6os, Smyd1, Socs2, Sox13, Spn, Sppl2b, Spry1, Srsf5, Srsf6, Srxn1, St3gal3, St6galnac3, Stx11, Tbx21, Tceal1, Tex2, Tg, Tgif1, Thsd7b, Thtpa, Tifa, Tjp2, Tnf, Tnfaip3, Tnfsf14, Tns4, Tpbg, Traf1, Trim39, Tshz1, Tspan14, Tuba1a, Tuba1c, Tuba4a, Tubb4b, Txk, Ublcp1, Ucp3, Usp20, Zbtb16, Zbtb7c, Zcchc18, Zfp36, Zfp831, Zfp959

Supplementary Table 4. DEGs between large intestinal control and Tfam-deficient γδT17 cells.

Category	Genes (Tfam-deficient versus control)
LI upregulated (193)	*1190002N15Rik, Aars, Abcb1b, Abce1, Aktip, Aldoc, Alg2, Amz1, Angptl6, Apoa1bp, Arl4c, Arv1, Asns, Atf5, Bbc3, Bcl6, Blvrb, Bola1, Cars, Ccdc107, Ccdc25, Ccl1, Ccr8, Cd27, Cdca7, Cdk5rap1, Cebpg, Chchd10, Chdh, Cited2, Clybl, Cpd, Crcp, Cryl1, Ctla2a, Cyb5r1, Cyp51, Dctpp1, Ddit3, Dennd2d, Dgcr6, Dnajc9, Dpf2, Dpp4, Dtnb, Dusp28, Eci2, Eef1b2, Eef1g, Eif4ebp1, Faah, Fam118a, Fam136a, Fam206a, Fam213a, Fam26f, Fam78a, Fam92a, Flot1, Fmo5, Galm, Gars, Gbp6, Gfer, Gm2a, Gm5595, Gnb2l1, Gns, Gpt2, Guk1, Hddc2, Hist1h1d, Hist1h4c, Hist2h2bb, Hlf, Hspa9, Hyi, Iars, Ifng, Igsf6, Il9r, Izumo1r, Kdelc2, Kdsr, Khk, Kif3a, Klf2, Lair1, Lars, Ldhb, Lif, Lilrb4a, Lyz2, Lztr1, Mars, Mboat1, Metrnl, Mpnd, Mrpl30, Mrpl57, Mrps28, Msto1, Mtg1, Mthfd2, Mvb12a, Nagk, Nars, Nek4, Nelfe, Noc2l, Nphp1, Nudt2, Nupr1, Nvl, Ogfod3, Ormdl1, P4ha2, Parn, Pck2, Phgdh, Pja2, Pogk, Ptger4, Ptgir, Pwp1, Rab27b, Rce1, Rhob, Rnf145, Rpl13, Rpl18, Rpl18a, Rpl23, Rpl29, Rpl30, Rpl34, Rpl4, Rpl7a, Rps15, Rps18, Rps19, Rps24, Rps3, Rps4x, Rps5, Rps8, Rpsa, Rsad2, Ruvbl2, Sars, Scamp1, Shmt2, Sigmar1, Slamf6, Slc1a4, Slc25a23, Slc35c1, Slc6a9, Slc7a3, Slc7a5, Sprn, Ssbp2, St7, Stom, Suclg1, Sumf1, Susd1, Tmem107, Tmem205, Tmem231, Tmem29, Tmem41a, Tmem9, Tnfrsf22, Tnfrsf26, Tnfsf10, Tpi1, Trappc4, Trappc6a, Trib3, Tsc22d3, Tspan31, Tspan32, Ubash3a, Ubc, Ubqln1, Uros, Vps8, Wrb, Yars, Yif1b, Zc4h2, Zfp759*
LI downregulated (403)	*2010111I01Rik, 2810417H13Rik, 4930550C14Rik, 5031414D18Rik, 8430408G22Rik, 9430020K01Rik, Abi3bp, Ablim3, Acoxl, Acpp, Acsbg1, Acta2, Actg2, Adcy3, Adgrg1, Adora2a, Agpat4, Agt, Akap12, Akna, Ang, Anks1, Anp32a, Anxa1, Arap3, Areg, Arhgap31, Arl5c, Arntl, Arrdc4, Ash1l, Atg7, Atoh8, Atp2a3, Bcl2a1a, Bcl2a1c, Bcl2a1d, Bcl2l1, Bhlhe40, Birc3, Blnk, Btk, C2cd2, Car2, Cblb, Ccdc125, Ccdc162, Ccdc88c, Ccnd1, Ccnl1, Ccnt1, Ccr10, Ccrl2, Cd163l1, Cd59a, Cd63, Cd83, Cdh13, Cenpl, Cep250, Cercam, Chad, Chst12, Clspn, Cnn1, Col12a1, Col5a1, Col5a3, Cpm, Cpxm1, Cpz, Crem, Crmp1, Cryab, Cryba4, Csrnp1, Ctgf, Cyp2j6, D930048N14Rik, Dag1, Ddr2, Ddx25, Dennd5a, Des, Dgkh, Dkk3, Dlg5, Dmpk, Dmrta1, Dot1l, Dusp4, Dusp6, Eaf2, Ece1, Ednrb, Egr1, Ehd2, Ehd4, Elf3, Ephx1, Esam, Etv3, Extl3, F3, Fam110a, Fam214a, Fasl, Fastkd3, Fbln5, Fbxo46, Fem1b,*

	Fem1c, Ffar4, Fhl1, Fkbp9, Flt3, Fn1, Fosl2, Foxo3, Frmd5, Fryl, Fscn1, Furin, Fxyd1, Gabarapl1, Gadd45b, Gdpd5, Gem, Gfod1, Gimap4, Gjb2, Glp1r, Gm15800, Gm166, Golga2, Gpr160, Gpr55, Gse1, Gys1, H3f3b, H6pd, Havcr2, Hilpda, Hip1r, Hivep1, Hopx, Hpse, Hs6st2, Hspg2, Htra1, Icam1, Ifi205, Ikzf3, Il1r1, Il2rb, Ildr1, Inpp1, Irf8, Itga5, Itgb2, Itgb5, Jam2, Jarid2, Junb, Jup, Kdm2b, Kdm6a, Kdm6b, Klf10, Klhl25, Klrb1c, Klrc1, Klrk1, Krt18, L3mbtl2, Lad1, Lama5, Lamb3, Lax1, Lims2, Litaf, Lpl, Lrmp, Lrrc75a, Lrrc8c, Lrrn2, Lum, Ly6c2, Ly6d, Lyn, Magi1, Mamdc2, Man1c1, Map3k14, Mapk6, March3, Mast4, Mcc, Mex3c, Mkl1, Mmp2, Mmp3, Mpzl1, Mrc2, Msl3l2, Msrb3, mt-Co1, mt-Cytb, mt-Nd1, mt-Nd2, mt-Nd4, mt-Nd5, mt-Nd6, Myh11, Myl9, Mylk, Myo1c, Myo1e, Mzb1, Nab2, Nbeal2, Nbl1, Nbr1, Ncoa7, Neu3, Nfatc1, Nfatc2, Nfic, Nfil3, Nfkbia, Nfkbiz, Nlrc5, Notch1, Nox1, Npnt, Nr1d1, Nr4a2, Nr4a3, Nrgn, Nrp1, Nusap1, Nxnl2, Ogn, Pabpc1l, Palld, Pcsk7, Pcyt1a, Pdcd1, Pdgfa, Pdzd2, Pglyrp1, Phactr2, Phc3, Pik3ap1, Pik3cg, Pik3r1, Pik3r5, Pim1, Pim3, Pira2, Pknox1, Plek, Plekhm3, Pls1, Plvap, Podnl1, Ppp1r16b, Ppp6r3, Prf1, Procr, Prom1, Prom2, Prr15l, Prr5l, Prss32, Psd2, Ptgs2, Ptpre, Ptprz1, Ptrf, R3hcc1l, Rab33b, Radil, Rbm41, Rbm5, Rbp7, Rdh10, Rel, Rem1, Rerg, Rest, Retnla, Rftn1, Rgs1, Rgs11, Rgs3, Rgs5, Rilpl2, Rin2, Rln3, Rnase2b, Rnd1, Rnf125, Rnf157, Rnf43, Rora, Rtn4rl1, Runx1, Runx2, S100a11, Samsn1, Sap130, Scube1, Sdcbp2, Sema3c, Sema4b, Serinc2, Serpina3f, Serpina3g, Serpinb6b, Sgip1, Sh2d2a, Sidt1, Sipa1, Ski, Skil, Slc15a3, Slc16a6, Slc22a15, Slc25a43, Slc27a6, Slc7a6os, Slc8b1, Slc9a3r2, Smad3, Smim3, Smoc2, Snx33, Sod3, Sorl1, Sox13, Sox18, Spry1, Srpk3, Srsf5, St14, Strn4, Stx11, Sulf1, Syne2, Synj2, Syt11, Tagln, Tbc1d1, Tbx21, Tesk2, Tex2, Tgfb1i1, Tgif1, Tmem88, Tnfaip2, Tnfaip3, Tnfrsf17, Tnfrsf1b, Tnfrsf9, Tnip1, Tns4, Tob2, Tox, Tpbg, Tpm1, Tpm2, Traf1, Traf3, Trim11, Trio, Trp53inp2, Tspan1, Tspan8, Tspyl4, Tubb6, Txndc5, Ube2l6, Ublcp1, Utf1, Vdr, Vgll4, Vps37b, Wipi1, Wsb1, Zbtb1, Zbtb16, Zbtb21, Zbtb24, Zbtb25, Zbtb46, Zc3h12a, Zfp36l1, Zfp870, Zfp871, Zmat4, Zswim4, Zswim8

Supplementary Table 5. DEGs between the small intestinal tissues of control and Tfam-deficient mice.

Category	Genes (Tfam-deficient versus control)
Upregulated (1217)	*1700019L03Rik, 1810011H11Rik, 1810041L15Rik, 1810046K07Rik, 2210011C24Rik, 3632451O06Rik, 4930404N11Rik, 5330417C22Rik, 6330403A02Rik, 8430408G22Rik, 9130008F23Rik, 9430020K01Rik, A4galt, A4gnt, AA467197, Aard, Ablim3, Abtb2, Acacb, Ackr3, Acsf2, Acsl1, Acsl3, Acss1, Acta2, Actc1, Actg2, Actn1, Adam33, Adam8, Adamts15, Adamts2, Adamts7, Adamtsl1, Adcy4, Adcy5, Adgrb2, Adgre4, Adgrg1, Adgrg3, Adm, Adora3, Adrb3, Aebp1, Afap1, Agbl3, Agtpbp1, Ahnak2, Aif1l, Ajuba, Ak1, Ak4, Aldh3b2, Aldoa, Aldoc, Alox15, Alox5, Alox5ap, Alyref2, Amy1, Ang4, Angpt2, Ank1, Ankrd22, Ankrd29, Ankrd35, Ankrd37, Ano1, Ano7, Antxr1, Anxa1, Anxa6, Aoc3, Apcdd1, Aplnr, Apobec2, Apobr, Apoh, Apold1, Aqp7, Aqp9, Arg1, Arhgap35, Arhgef15, Arhgef28, Arhgef37, Arhgef9, Armcx3, Arntl2, Arsi, Art3, Asic3, Aspn, Ass1, Atoh1, Atp1b2, Atp2a3, Atp2b4, Atp7b, AU021092, Avil, Avpi1, Avpr1a, Azin2, B4galt2, B930041F14Rik, Bace2, Bag2, Basp1, Batf3, BC051019, Bcam, Bcl6b, Bgn, Bhlha15, Bhlhe22, Bhlhe40, Bicc1, Bmx, Bnip3, Bok, Bves, C1qtnf1, C1qtnf6, C1qtnf9, C3, C4b, C4bp, C6, C7, Cabp1, Cacna1c, Cacna1h, Cacna1s, Cacna2d1, Cacna2d2, Cadm4, Calcb, Cald1, Calhm3, Camk2b, Camk2n2, Camkk1, Camkk2, Capn13, Capn9, Car4, Car8, Casp3, Casq2, Cav1, Cav2, Cbfa2t3, Cbl, Cbr2, Cbr3, Ccdc109b, Ccdc126, Ccdc129, Ccdc28b,*

	Ccdc62, Ccdc64, Ccdc8, Ccdc80, Ccdc92, Ccl11, Ccl24, Ccl8, Ccl9, Ccm2l, Ccnj, Ccr3, Cd248, Cd24a, Cd300ld, Cd300lf, Cd34, Cd44, Cd55, Cd59a, Cd84, Cd9, Cdh13, Cdh5, Cdkl2, Cdkl3, Cdkn2a, Cdr2l, Ceacam18, Ceacam2, Cebpe, Cela1, Cend1, Cercam, Ces1c, Ces2b, Cfh, Chad, Chat, Chpf, Chrdl1, Chrm3, Chst1, Chst12, Cib2, Cidea, Cidec, Cilp, Cish, Cited4, Ckb, Ckm, Clca1, Clca2, Cldn5, Cldn9, Clec14a, Clip3, Clip4, Clps, Cma1, Cma2, Cnn1, Cnpy1, Col12a1, Col15a1, Col16a1, Col18a1, Col1a1, Col1a2, Col24a1, Col3a1, Col4a1, Col4a2, Col5a1, Col5a2, Col5a3, Col6a1, Col6a2, Col6a3, Col6a5, Colec10, Copz2, Coro6, Cox4i2, Cpa3, Cpe, Cpeb1, Cped1, Cpne5, Cpvl, Cpxm1, Cpxm2, Cracr2a, Creb3l1, Creb3l4, Crip2, Crlf1, Crtap, Cryab, Csdc2, Csf2rb, Csf2rb2, Csn3, Csrp1, Ctgf, Ctla2a, Ctsk, Ctsl, Ctso, Ctxn1, Ctxn3, Cutal, Cwh43, Cxcl16, Cybrd1, Cygb, Cyp11a1, Cyp2e1, Cyp2j9, Cyp4f18, Cyp7b1, Cyr61, Cysltr1, Cyyr1, Dact3, Dbn1, Dcbld2, Dclk1, Ddah2, Ddr2, Ddx26b, Defa17, Defa24, Defa26, Defa3, Defa-rs7, Defb1, Des, Dhrs1, Diras2, Dkk2, Dmpk, Dmxl2, Dnah8, Dnajb5, Dner, Dock9, Dok2, Dpep2, Dpysl2, Dpysl3, Dtna, Duox2, Duoxa1, Duoxa2, Dusp1, Dusp18, Dusp3, Dusp4, Dusp5, E130012A19Rik, E330009J07Rik, Ear2, Ecm1, Ecscr, Edn1, Ednra, Eef2k, Efemp1, Efemp2, Efhd1, Egfl7, Egln3, Ehd2, Eid2, Eln, Emilin1, Emilin2, Emp3, Enah, Endod1, Enpp3, Epdr1, Ephb3, Epn3, Erg, Ern2, Ero1l, Ero1lb, Esam, Ethe1, Etv4, Eva1b, Evpl, Exoc3l, Fabp4, Fabp5, Fads3, Fads6, Fam101a, Fam124a, Fam129a, Fam161a, Fam171a1, Fam171a2, Fam174b, Fam19a3, Fam221a, Fam222a, Fam3c, Fam43a, Fam46b, Fam46c, Fam57a, Fam64a, Fam73a, Fam73b, Fam83b, Faxc, Fbln2, Fbln7, Fbn1, Fbxl16, Fbxl21, Fbxl22, Fbxo2, Fbxo44, Fcer1a, Fcna, Fcrlb, Fdps, Fer1l4, Fer1l6, Fes, Fetub, Ffar2, Ffar3, Fgfr4, Fhl1, Fhl2, Fhl3, Filip1l, Fkbp10, Fkbp11, Fkbp14, Fkbp9, Flna, Flnb, Flnc, Flrt3, Fn1, Fnip2, Folr2, Foxp2, Frem2, Frmd5, Frmd6, Frzb, Fstl1, Fstl3, Fut2, Fxyd1, Fxyd3, Fxyd5, Fxyd6, Fyb, Fzd2, Fzd4, Fzd9, G0s2, G6pdx, Gabre, Galnt18, Galnt5, Gapt, Garnl3, Gata2, Gata3, Gatsl3, Gcgr, Gfi1b, Gfra4, Gga2, Ggh, Gja1, Gjc2, Gkn3, Glce, Glipr2, Glt1d1, Gm10384, Gm11627, Gm13889, Gm14137, Gm15284, Gm15292, Gm15299, Gm20939, Gm2a, Gm38394, Gm4980, Gm5148, Gm6696, Gm684, Gm7325, Gm766, Gm8113, Gm973, Gm9938, Gml, Gml2, Gmpr, Gna14, Gnai1, Gnat1, Gnat3, Gne, Gng13, Got1, Gp1ba, Gpc1, Gpc2, Gpc6, Gper1, Gpi1, Gpm6b, Gpnmb, Gpr153, Gpr161, Gpr183, Gpr55, Gprc5c, Gpsm1, Gpx3, Grasp, Grem1, Grk5, Grp, Grrp1, Gsdma2, Gsdmc2, Gsdmc3, Gsdmc4, Gsn, Gsto1, Gucy1b3, Gyg, Gys1, H2-Q10, Habp2, Hacd4, Hapln4, Hck, Hcn1, Hcn2, Hcn3, Hcrtr1, Hdc, Hebp2, Hey1, Hgfac, Hid1, Higd1b, Hivep3, Hk1, Hlf, Hmcn1, Hmgn3, Hmox1, Hmx2, Hmx3, Hnmt, Homer3, Hoxa2, Hoxa3, Hoxa7, Hoxc8, Hpd, Hpgds, Hpse2, Hs3st1, Hsbp1l1, Hsd11b1, Hspb1, Hspb6, Hspb7, Htra1, Htra3, Hyal1, Id4, Iffo2, Ifi27l2b, Ifitm1, Igf1, Igfbp2, Igfbp4, Igfbp6, Igfbp7, Igsf23, Il13, Il17b, Il17rb, Il18r1, Il1a, Il1rl1, Il1rn, Il4, Il9r, Inca1, Inhba, Inhbb, Inpp5d, Insig1, Insrr, Itga2b, Itga5, Itga7, Itgb3, Itpr2, Iyd, Jph2, Kank2, Kcnb1, Kcnc3, Kcnd3, Kcne4, Kcnf1, Kcnh2, Kcnh3, Kcnh6, Kcnip2, Kcnip3, Kcnj16, Kcnj2, Kcnma1, Kcnmb1, Kcnq1, Kcnq4, Kctd1, Kctd12, Kctd15, Kctd17, Kif1a, Kif26a, Kirrel3, Kit, Klf2, Klhdc7a, Klhdc8a, Klhdc8b, Klk1, Krt17, Krt18, Krt23, Krt7, Krt79, Krt80, Krt84, Lama3, Lamc2, Large, Lbh, Lcor, Ldha, Ldlrad3, Ldlrad4, Lect2, Lgals1, Lgi3, Lilr4b, Limk1, Lims2, Lin7b, Lix1l, Lmcd1, Lmod1, Lox, Loxl1, Loxl2, Loxl4, Lpin1, Lpl, Lrmp, Lrp11, Lrrc10b, Lrrfip1, Ltbp1, Ltbp3, Ltc4s, Lum, Ly6c2, Ly6d, Ly6g6d, Ly6g6f, Lyl1, Lynx1, Madcam1, Map1a, Map1b, Marcks, Marveld1, Matk, Matn2, Mcam, Mcpt1, Mcpt2, Mcpt4, Mcpt9, Mctp1, Mdfi, Me1, Mecom, Medag, Mfap2, Mfap3l, Mfsd4, Mgl2, Mgll, Mical3, Mlph, Mmp10, Mmp11, Mmp14, Mmp15, Mmp17, Mmp19, Mmp2, Mmp23, Mmp25, Mmp3, Mmp9, Mns1, Mnx1, Mpp3, Mptx1, Mptx2, Mr1, Mrc2, Mrgpra9, Mrgpre, Mrgprf, Mrvi1, Ms4a2, Msi1, Msrb1, Msrb2, Msrb3, Mt3, Mtss1l, Muc2, Muc3a, Mustn1, Mycn, Myh10, Myh11, Myl9, Myo18b, Myo1b, Myo1c, Myo5c, Myocd, Myom1, Myrip, Naaa, Napepld, Natd1, Nav2, Ncf1, Ncs1, Ndrg2, Ndufa4l2, Neb, Nebl, Nedd9, Nes, Neto1, Neu3, Neurl1a, Nexn, Nfe2, Nlgn2, Nmu, Nmur1, Nmur2, Nol3, Nos3, Notch3, Notch4, Notum, Npas4, Npdc1, Nptx2, Nr2f1, Nr4a2, Nradd, Nrep, Nrgn, Nrp2, Nrros,

	Nrtn, Nt5c2, Nt5dc2, Nt5e, Ntng2, Nucb2, Nxpe4, Oas1c, Oas1f, Oaz2, Obfc1, Odf2l, Ogdhl, Olfml2b, Oosp1, Opn3, Optc, Otud3, Oxct1, P2rx1, P2rx7, P2ry14, P3h1, P3h2, P3h4, P4ha1, P4ha2, Pacsin1, Pacsin3, Padi2, Pak6, Palmd, Pam, Pamr1, Paqr8, Pcdh7, Pcolce, Pcp4, Pcp4l1, Pcsk6, Pcx, Pcyox1l, Pde2a, Pde7a, Pde8a, Pdlim3, Pdlim4, Pdlim7, Pdzd2, Pdzd7, Pea15a, Pecam1, Penk, Per3, Pex5l, Pfkl, Pfkm, Pgap1, Pgf, Pgm2l1, Pgm5, Phf21b, Phlda1, Phlda2, Phlda3, Phldb2, Phospho1, Pi16, Piezo2, Pik3c2g, Pik3cg, Pik3r3, Pik3r5, Pla1a, Pla2g10, Pla2g2e, Pla2g2f, Pla2g4a, Pla2g4c, Pla2g7, Plagl1, Plcb1, Plcb2, Plcb4, Plcg2, Plcl1, Plek, Plekhg1, Plekhg3, Plk2, Plod1, Pls3, Pltp, Plxnb1, Plxnd1, Pmepa1, Pmm1, Pnck, Pnliprp1, Pnliprp2, Postn, Pou2af1, Pou2f3, Ppef1, Ppef2, Ppic, Ppl, Ppm1e, Ppm1j, Ppp1r12b, Ppp1r14a, Ppp1r14c, Ppp1r16b, Ppp1r3b, Ppp1r3c, Ppp1r9a, Ppp2r3a, Prkaa2, Prkcb, Prkcdbp, Prnp, Proc, Prom2, Pros1, Prox1, Prss35, Prss53, Prune2, Ptafr, Ptges, Ptgis, Ptgs2, Pth1r, Ptpdc1, Ptpn18, Ptpn7, Ptprn2, Ptpro, Ptprt, Ptrf, Pygl, Rab11fip5, Rab15, Rab27a, Rab27b, Rab44, Rac2, Ramp1, Ramp2, Ramp3, Rap1gap, Rarb, Rasa4, Rasgef1b, Rasgrp3, Rasgrp4, Rasip1, Raver2, Rbm38, Rbp1, Rbp2, Rbp4, Rbpms, Rbpms2, Rcan3, Rcn3, Rec8, Reep2, Reep5, Reg4, Rem1, Rep15, Retnla, Retnlb, Retnlg, Rexo2, Rgs11, Rgs13, Rgs18, Rgs22, Rgs4, Rgs5, Rhbdl3, Rhoj, Rnase1, Rnase2a, Rnd1, Rnf183, Rnf207, Rnf32, Rprml, Rps6ka2, Rras, Rsad1, Rspo3, Rtn2, Rundc3a, Ryr3, S100a1, S100a13, S100a6, S100g, S1pr1, Samd14, Samd4, Samd9l, Samsn1, Scarb1, Scd1, Scgb2b17, Scgb2b20, Scgb3a1, Scin, Scn1b, Scnn1a, Scube2, Sdc3, Selm, Selp, Sema3g, Sema4c, Sema6c, Sema7a, Sepn1, Serp2, Serpina3n, Serpinb5, Serpine1, Serpine2, Serpinf1, Serpinf2, Serping1, Serpinh1, Serpini1, Setbp1, Sez6l2, Sftpa1, Sfxn3, Sgca, Sgcd, Sh2d6, Sh2d7, Sh3bgr, Sh3d21, Sh3kbp1, Sh3pxd2b, Sh3rf2, Sh3yl1, Shf, Shisa4, Siglecf, Sik1, Six5, Slamf9, Slc16a1, Slc16a12, Slc16a3, Slc16a7, Slc17a9, Slc18a1, Slc18a2, Slc18a3, Slc1a4, Slc20a1, Slc23a3, Slc25a12, Slc27a1, Slc28a1, Slc28a2, Slc2a1, Slc2a10, Slc2a4, Slc2a8, Slc35e4, Slc35g2, Slc36a4, Slc45a3, Slc4a7, Slc4a8, Slc5a9, Slc6a17, Slc8a2, Slc9a3, Slco4a1, Slit2, Slk, Smim1, Smim3, Smpx, Smtn, Smyd1, Snai2, Sncaip, Snrnp25, Soat1, Sobp, Sod3, Sorbs2, Sorcs2, Sox17, Sox18, Sox4, Sox9, Spaca4, Spaca6, Sparc, Spdef, Speg, Spib, Spink4, Spon1, Spon2, Sprr2a3, Spry1, Spta1, Sptb, Srgn, Srpx2, Ssc5d, St18, St3gal2, St3gal3, St3gal5, St3gal6, St6galnac6, Stk32c, Stk38l, Stmn3, Stra6l, Strip2, Stxbp1, Sucnr1, Sulf1, Sult4a1, Susd1, Sval1, Sybu, Sycn, Syngap1, Syngr1, Synj2, Synm, Synpo2, Syt15, Sytl4, Tagln, Tal1, Tbxa2r, Tcaf1, Tcaf2, Tcf21, Tead2, Tenm3, Tesc, Tespa1, Tg, Tgfb1i1, Thbs1, Thbs2, Thbs3, Thrsp, Thsd4, Timp1, Timp2, Tle6, Tlr4, Tm6sf1, Tmed6, Tmeff1, Tmem100, Tmem116, Tmem120b, Tmem121, Tmem132a, Tmem141, Tmem198, Tmem200b, Tmem229a, Tmem255b, Tmem38a, Tmem44, Tmem45a, Tmem47, Tmem71, Tmem88, Tmtc1, Tnc, Tnfrsf11b, Tnfrsf12a, Tnfrsf21, Tnfrsf23, Tnfsf13b, Tnnt3, Tns1, Tox2, Tox3, Tpd52l1, Tph1, Tpm2, Tpsb2, Tpsg1, Trak1, Trak2, Treml2, Trib2, Trim46, Trim47, Trnp1, Trp53inp2, Trpm5, Trpv2, Tsc22d1, Tspan2, Tspan6, Ttc28, Ttll11, Ttll7, Tuba1a, Tubb2b, Tubb3, Tubb4a, Tubb6, Twist1, U90926, Ugt1a9, Unc45b, Unc5a, Unc93a, Upb1, Utp14b, Vash1, Vasn, Vav1, Vcan, Vcl, Vim, Vmn2r26, Vsig2, Vstm4, Vtn, Vwf, Wee2, Wipi1, Wnt5a, Wscd1, Wwtr1, Zdhhc14, Zeb2, Zfand2a, Zfp37, Zfp428, Zfp667, Zfp697, Zfp775, Zfp871, Zfp9, Zscan2
Downregulated (621)	*1500009L16Rik, 1700011H14Rik, 1700019G17Rik, 1700024P16Rik, 1810065E05Rik, 2010001E11Rik, 2010005H15Rik, 2010106E10Rik, 2210407C18Rik, 2310007B03Rik, 4931406C07Rik, 9930012K11Rik, AA986860, Aadac, Abca1, Abcb1a, Abcg2, Abcg5, Abcg8, Abhd6, Acot12, Acsm5, Acta1, Acy1, Adamtsl5, Adap1, Adgrd1, Adgrg5, Adh1, Adh6a, Adk, Adprm, Afp, Agmat, Agmo, Agpat9, Aim1, Akr1b7, Akr1c19, Aldh1a1, Aldh1a7, Aldh1l1, Aldh4a1, Aldh9a1, Als2cr12, Amn, Angptl4, Ano2, Anpep, Anxa13, Aoc1, Apitd1, Aplp1, Apoc3, Apol10a, Apol11b, Apol7b, Apol7e, Aqp1, Aqp3, Aqp4, Arhgef10l, Arl4d, Asah2, Asns, Aspa, Aspg, AY761184, B2m, B3galt5, B3glct, B3gnt6, B4galnt4, Baat, Bach2,*

	Baiap3, Barx2, BC025446, BC089597, Bcl2l15, Bco1, Bend7, Bex1, Bfsp1, Bmp3, Bmp8b, Btla, Btnl6, C2, C2cd4a, C530008M17Rik, Cadm1, Canx, Car13, Car2, Card11, Card9, Cars, Casp1, Casp4, Casp8, Cat, Ccdc14, Ccl20, Ccl22, Ccl25, Ccl28, Ccl5, Ccnd1, Ccr5, Ccrn4l, Cd14, Cd160, Cd177, Cd247, Cd27, Cd274, Cd302, Cd36, Cd38, Cd3d, Cd3e, Cd3g, Cd6, Cd7, Cd72, Cd74, Cd86, Cd8a, Cd8b1, Cd96, Cdca7l, Ceacam1, Ceacam10, Ces1e, Ces1f, Ces2a, Chac1, Chst4, Ciita, Clca3a1, Clca3a2, Clca3b, Clca4a, Clca4b, Cldn2, Cldn8, Clic5, Cmpk2, Cndp1, Cndp2, Cobl, Cox6b2, Cpm, Crip1, Cryl1, Ctdspl, Cth, Ctrl, Ctsw, Cubn, Cxcl10, Cxcl13, Cxcl9, Cxcr3, Cxcr6, Cyba, Cyp27a1, Cyp2b10, Cyp2c65, Cyp2c68, Cyp2d22, Cyp3a11, Cyp3a13, Cyp3a25, Cyp4b1, Cyp4f40, Cyp4v3, D130043K22Rik, Dctd, Ddah1, Ddc, Defa21, Defa22, Defa-rs1, Dfna5, Dhrs11, Dio1, Dmbt1, Dnaaf1, Dnase1, Dpf3, Dpp4, Dpyd, Dtx1, E2f8, Efr3b, Enpep, Enpp7, Epb4.1l3, Epb4.1l4a, Eps8, Ereg, Exo1, Exoc3l4, Ezr, Faah, Fabp2, Fabp6, Fam118a, Fam132a, Fam151a, Fam213b, Fam26f, Far2, Farp2, Fbp1, Fbp2, Fcamr, Fcgbp, Fcgr1, Fcrl1, Ffar4, Fgfbp1, Fmo2, Fmo4, Fst, G630090E17Rik, G6pc, Gal3st2, Gbp10, Gbp2, Gbp3, Gbp4, Gbp5, Gbp6, Gbp7, Gbp8, Gcnt1, Gemin6, Ggct, Ggt1, Gimap3, Gimap4, Gimap7, Gip, Gjb2, Gjb3, Glod5, Glrx, Glud1, Gm10104, Gm11127, Gm11437, Gm12216, Gm13199, Gm15293, Gm15315, Gm28051, Gm4951, Gm7030, Gm8909, Gm9994, Gp1bb, Gpr160, Gpr17, Gpr18, Gpt, Gramd1b, Gramd1c, Grina, Gsta1, Gsta3, Gsta4, Gzma, Gzmb, H2-Aa, H2-Ab1, H2-DMa, H2-DMb1, H2-DMb2, H2-Eb1, H2-M2, H2-Q1, H2-Q2, H2-Q4, H2-Q6, H2-Q7, H2-T10, H2-T23, H2-T3, Hao2, Hes2, Hk2, Hmga2, Hnf4g, Hsd17b13, Hsd17b6, Hsd3b3, Hspa12a, Hyi, Id1, Ido1, Ifi205, Ifi47, Ifit1bl1, Igtp, Iigp1, Il12rb1, Il18, Il18bp, Il21r, Il2rb, Il7, Il7r, Irf8, Irgm1, Irgm2, Isoc1, Itgad, Itk, Itpka, Kbtbd11, Kcnj10, Kctd5, Kif21b, Klra2, Klrb1b, Klrd1, Krt20, Ky, Lap3, Lck, Lct, Leap2, Lgr5, Lilra5, Lipe, Lrr1, Lrrc19, Lrrc75a, Ltb, Ltbp2, Ltf, Lypd8, Lyz1, Lyz2, Maf, Mafb, Maoa, Maob, Map3k6, Mapkbp1, Me2, Mep1a, Mep1b, Mettl7b, Mfhas1, Mfsd7b, Mgam, Mgst1, Mgst2, Mid1, Mid2, Mme, Mmp13, Mob3c, Mocs1, Mocs2, Mov10, Mreg, Mrm1, Mro, Mroh7, Ms4a12, Ms4a18, Ms4a4b, Mtf1, Mthfd1, Mthfd2, Muc13, Mx2, Myo1a, Myo7a, Myom3, Naaladl1, Nars, Ndufa4, Nfil3, Nfkbia, Nfkbiz, Nkg7, Nlrc5, Nmi, Nos2, Nostrin, Nox1, Npc1l1, Nr1h3, Nr1h4, Nudt5, Nxf7, Oat, Ocm, Odf3b, Olfr165, Olfr56, Orc1, Osbpl1a, Otc, P2ry2, P2ry4, Panx1, Papln, Pax8, Pbld2, Pdk3, Pdlim2, Pdzd3, Pdzk1, Pepd, Perm1, Pglyrp2, Phgr1, Pianp, Pik3ap1, Pla2g5, Plb1, Plekhn1, Plet1, Pls1, Plscr1, Podnl1, Polr2k, Pram1, Prf1, Prodh, Prr15, Prr5l, Prrt1, Prss12, Prss27, Prss30, Psmb8, Psmb9, Psme2, Psme2b, Ptgr1, Ptk6, Ptpn22, Rab19, Rasd1, Rasl12, Rassf4, Rbp7, Rdh7, Rdh9, Reg3a, Reg3b, Reg3g, Retsat, Rhebl1, Rmi2, Rnf145, Rnf180, Rnf208, Rnps1, Rundc3b, Rwdd2b, S100a10, Saa2, Saa3, Satb2, Scrn2, Sec14l2, Sec14l4, Sectm1b, Sema6a, Serpina1b, Sesn1, Sgk1, Sgpl1, Sh2d2a, Shank2, Shroom1, Sla2, Slamf8, Slc10a2, Slc10a5, Slc13a1, Slc13a2, Slc15a1, Slc16a10, Slc16a5, Slc22a4, Slc22a5, Slc23a1, Slc25a13, Slc25a15, Slc25a36, Slc25a37, Slc25a45, Slc25a48, Slc26a2, Slc27a4, Slc28a3, Slc30a1, Slc34a2, Slc35f2, Slc35f5, Slc35g1, Slc36a1, Slc3a1, Slc3a2, Slc40a1, Slc41a3, Slc43a2, Slc51a, Slc51b, Slc52a3, Slc5a11, Slc5a12, Slc5a4b, Slc5a6, Slc5a8, Slc6a14, Slc6a19, Slc6a20a, Slc6a20b, Slc7a15, Slc7a7, Slc7a8, Slc9a3r1, Slco2a1, Slfn9, Smad6, Smpdl3b, Snph, Soat2, Socs3, Sowaha, Spink1, St3gal1, Stat1, Steap1, Stk10, Stom, Sult1d1, Suox, Susd2, Suv39h1, Syne4, Tap1, Tap2, Tat, Tcea3, Tfg, Tfpi, Tgtp1, Tgtp2, Tha1, Thpo, Ticrr, Tifa, Tigit, Tlcd2, Tldc2, Tle4, Tlr2, Tm4sf5, Tmc5, Tmcc3, Tmem236, Tmem86b, Tmigd1, Tnf, Tnfaip8l2, Tnfaip8l3, Tnfrsf14, Tnfsf10, Trat1, Treh, Trib3, Trpm2, Trpm6, Tsku, Ttc36, Ttll2, Ttyh3, Tubal3, Tvp23a, Tymp, Ubd, Ugt1a1, Ugt1a6a, Ugt2a3, Ugt2b34, Ugt2b36, Ugt2b5, Uhrf1, Upk1b, Upp1, Urah, Usp2, Vnn1, Vsig10, Vwa1, Wdpcp, Wnt6, Xcl1, Xlr3a, Xpnpep1, Xpnpep2, Zap70, Zbp1, Zc3h12a, Zcchc11, Zdhhc15, Zdhhc19, Zfp385b, Zg16

Supplementary Table 6. Primer sequences used.

Primer name	Sequence (5' - 3')
Actb-F	TGTGACGTTGACATCCGTAA
Actb-R	GCTAGGAGCCAGAGCAGTAA
Tfam-F	CCAAAAAGACCTCGTTCAGC
Tfam-R	ATGTCTCCGGATCGTTTCAC
genome-β-globin-F	GAGTTGAGACTGTGCTTGGC
genome-β-globin-R	TCTGCACCCAAATCATTGTT
Dclk1-F	GCTGTCAGTAGCTGGCAAAA
Dclk1-R	CAAGAGCGGTGGTTGCTATT
Trpm5-F	CCAGCATAAGCGACAACATCT
Trpm5-R	GAGCATACAGTAGTTGGCCTG
Plcb2-F	CTCGCTTTGGGAAGTTTGC
Plcb2-R	GCATTGACTGTCATCGGGT
Gnat3-F	GTTCAGAGAGCAAGGAATCAGCC
Gnat3-R	GTGCTTTTCCCAGATTCACCTGC
Il4-F	AACTCCATGCTTGAAGAAGAACTC
Il4-R	CCAGGAAGTCTTTCAGTGATGTG
Il25-F	TGGAGCTCTGCATCTGTGTC
Il25-R	TCAAGTCCCTGTCCAACTCA
Il5-F	GTTGACAAGCAATGAGACGATGAG
Il5-R	CCCACGGACAGTTTGATTCTTC
Il13-F	GCAACATCACACAAGACCAGAC
Il13-R	GAATCCAGGGCTACACAGAACC
Relmb-F	TGGTGGATCAAAGGATCAAG
Relmb-R	CCACAAGCACATCCAGTGAC
Reg3g-F	CAAGGTGAAGTTGCCAAGAA
Reg3g-R	CCTCTGTTGGGTTCATAGCC
Igf1-F	ACCGAGGGGCTTTTACTTCA
Igf1-R	TGGCTCACCTTTCCTTCTCC
Igfbp2-F	CTGAAGGCGCTTGTCACAGG
Igfbp2-R	AAGGCGCATGGTGGAGATCC
Igf2-F	GATCCCAGTGGGGAAGTCG
Igf2-R	GCTGGACATCTCCGAAGAGGCTC
Igfbp6-F	GGTCTACAGCCCTAAGTGCG
Igfbp6-R	GCAGGGGCCCATCTCACTAT
Igf1r-F	GGAGAAGCCCATGTGTGAG
Igf1r-R	GTCGTGGATAACGAAGCCATC
Igf2r-F	CCAACAGCTACCGGATGTCTG
Igf2r-R	ATTCCCACCACAAGGATAGC

Igfbp4-F	GAAGCCATCCAGGAAAGCCTG
Igfbp4-R	CTCGCTCTGGCAGGAACCCT
Igfbp7-F	AAGAGGCGGAAGGGTAAAGC
Igfbp7-R	TGGGGTAGGTGATGCCGTT
Tritrichomonas-F	AGAGGAAGGAGAAGTCGTAACAAGG
Tritrichomonas-R	CTCGTGTAAGAAGCCAAGACATCC
Eubacteria-F	ACTCCTACGGGAGGCAGCAGT
Eubacteria-R	ATTACCGCGGCTGCTGGC
