# Supplementary material for: Mitochondrial transcription factor A in RORγt+ lymphocytes regulate small intestine homeostasis and metabolism
Source: Nat Commun. 2021 Jul 22;12:4462. doi: 10.1038/s41467-021-24755-9 (PMC8298438; doi:10.1038/s41467-021-24755-9)
Supplement: Supplementary file 2 — Reporting summary [file 41467_2021_24755_MOESM2_ESM.pdf]

nature research

Corresponding author(s): Liang Zhou

Last updated by author(s): 2021/06/15

Reporting Summary

Nature Research wishes to improve the reproducibility of the work that we publish. This form provides structure for consistency and transparency in reporting. For further information on Nature Research policies, see our Editorial Policies and the Editorial Policy Checklist.

Statistics

For all statistical analyses, confirm that the following items are present in the figure legend, table legend, main text, or Methods section.

n/a	Confirmed	
☐	☒	The exact sample size (*n*) for each experimental group/condition, given as a discrete number and unit of measurement
☐	☒	A statement on whether measurements were taken from distinct samples or whether the same sample was measured repeatedly
☐	☒	The statistical test(s) used AND whether they are one- or two-sided<br>*Only common tests should be described solely by name; describe more complex techniques in the Methods section.*
☒	☐	A description of all covariates tested
☐	☒	A description of any assumptions or corrections, such as tests of normality and adjustment for multiple comparisons
☐	☒	A full description of the statistical parameters including central tendency (e.g. means) or other basic estimates (e.g. regression coefficient) AND variation (e.g. standard deviation) or associated estimates of uncertainty (e.g. confidence intervals)
☐	☒	For null hypothesis testing, the test statistic (e.g. *F*, *t*, *r*) with confidence intervals, effect sizes, degrees of freedom and *P* value noted<br>*Give P values as exact values whenever suitable.*
☒	☐	For Bayesian analysis, information on the choice of priors and Markov chain Monte Carlo settings
☒	☐	For hierarchical and complex designs, identification of the appropriate level for tests and full reporting of outcomes
☒	☐	Estimates of effect sizes (e.g. Cohen's *d*, Pearson's *r*), indicating how they were calculated

Our web collection on statistics for biologists contains articles on many of the points above.

Software and code

Policy information about availability of computer code

Data collection	BD FACS Diva v8.0.2 has been used to collect flow cytometry data. ZEN 2011 v14.0.10.201 has been used to collect confocal images.
Data analysis	GraphPad Prism v7.04 has been used to analyze and visualize the data.<br>For RNAseq FastQC v0.11.5 was used to ensure high per-base sequence quality of reads.<br>STAR v2.7.4a was used to align and acquire raw count values then RSEM v1.2.25 was used to quantify FPKM expression values.<br>R v3.6.2 with the following packages was used to perform downstream analysis and visualization of RNAseq data (DEseq2 v1.26.0, pheatmap v1.0.12, rgl v0.100.30).<br>Expression scatter plots were produced with Excel(version2105 from Microsoft 365).<br>Kegg pathway and GO analysis were analyzed with online Kegg and GO website at GSEA (MSigDB v6.0, http://software.broadinstitute.org/gsea/index.jsp).<br>FlowJo v10.2 has been used to analyze flow cytometry data.<br>For Metabolite data MZmine2 (freeware, v2.53) was used for alignment, gap filling and metabolite identification then PCA was performed using Metaboanalyst 3.0.

For manuscripts utilizing custom algorithms or software that are central to the research but not yet described in published literature, software must be made available to editors and reviewers. We strongly encourage code deposition in a community repository (e.g. GitHub). See the Nature Research guidelines for submitting code & software for further information.

Data

Policy information about availability of data

All manuscripts must include a data availability statement. This statement should provide the following information, where applicable:

- Accession codes, unique identifiers, or web links for publicly available datasets
- A list of figures that have associated raw data
- A description of any restrictions on data availability

All data supporting the findings of this study are available within the article and its supplementary materials and from the corresponding author upon reasonable request. The RNA-sequencing data of splenic, small intestinal and large intestinal control and Tfam-deficient gdT17 cells, and small intestine tissues of control and Tfamfl/flRorc-cre mice generated in this study have been deposited in the NCBI database under accession code GSE152535 Source data are provided with this paper.

Field-specific reporting

Please select the one below that is the best fit for your research. If you are not sure, read the appropriate sections before making your selection.

- [x] Life sciences
- [ ] Behavioural & social sciences
- [ ] Ecological, evolutionary & environmental sciences

For a reference copy of the document with all sections, see nature.com/documents/nr-reporting-summary-flat.pdf

Life sciences study design

All studies must disclose on these points even when the disclosure is negative.

Sample size	No sample size calculation was performed. The exact n values used to calculate the statistics are provided and a reasonable sample size was chosen to ensure adequate reproducibility of results. The sample size was chosen equal or more than 3 to run a student's t-test or other statistics analysis.
Data exclusions	Data were excluded when sorted gdT17 cells were of poor quality, for example decreased viability (<50%) due to the long time kept on ice and measurement were failed. Cytokine data were excluded when the PMA and Ionomycin stimulation failed to induced cytokine in any samples.
Replication	RNA-seq and metabolome included 3 duplicates for each group. All attempts at replication for RNA-seq and metabolome were successful. Other experiments were replicated several times (2-4) with reproducible results, as indicated in each figure legend.
Randomization	For experiments comparing knockout vs control mice, or different gdT cell subsets from control mice, or gdT17 cells from different tissues, aged matched male and female littermate mice were randomized.
Blinding	Figure 3c, d: the pathologist who evaluated samples for histology scores was blinded of genotypes during evaluation.<br>Other figures were not blinded since the experiment performing, data collection and analysis were completed by the same author, although the results were confirmed by the rest authors.

Behavioural & social sciences study design

All studies must disclose on these points even when the disclosure is negative.

Study description	*Briefly describe the study type including whether data are quantitative, qualitative, or mixed-methods (e.g. qualitative cross-sectional, quantitative experimental, mixed-methods case study).*
Research sample	*State the research sample (e.g. Harvard university undergraduates, villagers in rural India) and provide relevant demographic information (e.g. age, sex) and indicate whether the sample is representative. Provide a rationale for the study sample chosen. For studies involving existing datasets, please describe the dataset and source.*
Sampling strategy	*Describe the sampling procedure (e.g. random, snowball, stratified, convenience). Describe the statistical methods that were used to predetermine sample size OR if no sample-size calculation was performed, describe how sample sizes were chosen and provide a rationale for why these sample sizes are sufficient. For qualitative data, please indicate whether data saturation was considered, and what criteria were used to decide that no further sampling was needed.*
Data collection	*Provide details about the data collection procedure, including the instruments or devices used to record the data (e.g. pen and paper, computer, eye tracker, video or audio equipment) whether anyone was present besides the participant(s) and the researcher, and whether the researcher was blind to experimental condition and/or the study hypothesis during data collection.*
Timing	*Indicate the start and stop dates of data collection. If there is a gap between collection periods, state the dates for each sample cohort.*
Data exclusions	*If no data were excluded from the analyses, state so OR if data were excluded, provide the exact number of exclusions and the rationale behind them, indicating whether exclusion criteria were pre-established.*

Non-participation	*State how many participants dropped out/declined participation and the reason(s) given OR provide response rate OR state that no participants dropped out/declined participation.*
Randomization	*If participants were not allocated into experimental groups, state so OR describe how participants were allocated to groups, and if allocation was not random, describe how covariates were controlled.*

Ecological, evolutionary & environmental sciences study design

All studies must disclose on these points even when the disclosure is negative.

Study description	*Briefly describe the study. For quantitative data include treatment factors and interactions, design structure (e.g. factorial, nested, hierarchical), nature and number of experimental units and replicates.*
Research sample	*Describe the research sample (e.g. a group of tagged Passer domesticus, all Stenocereus thurberi within Organ Pipe Cactus National Monument), and provide a rationale for the sample choice. When relevant, describe the organism taxa, source, sex, age range and any manipulations. State what population the sample is meant to represent when applicable. For studies involving existing datasets, describe the data and its source.*
Sampling strategy	*Note the sampling procedure. Describe the statistical methods that were used to predetermine sample size OR if no sample-size calculation was performed, describe how sample sizes were chosen and provide a rationale for why these sample sizes are sufficient.*
Data collection	*Describe the data collection procedure, including who recorded the data and how.*
Timing and spatial scale	*Indicate the start and stop dates of data collection, noting the frequency and periodicity of sampling and providing a rationale for these choices. If there is a gap between collection periods, state the dates for each sample cohort. Specify the spatial scale from which the data are taken*
Data exclusions	*If no data were excluded from the analyses, state so OR if data were excluded, describe the exclusions and the rationale behind them, indicating whether exclusion criteria were pre-established.*
Reproducibility	*Describe the measures taken to verify the reproducibility of experimental findings. For each experiment, note whether any attempts to repeat the experiment failed OR state that all attempts to repeat the experiment were successful.*
Randomization	*Describe how samples/organisms/participants were allocated into groups. If allocation was not random, describe how covariates were controlled. If this is not relevant to your study, explain why.*
Blinding	*Describe the extent of blinding used during data acquisition and analysis. If blinding was not possible, describe why OR explain why blinding was not relevant to your study.*

Did the study involve field work? ☐ Yes ☒ No

Field work, collection and transport

Field conditions	*Describe the study conditions for field work, providing relevant parameters (e.g. temperature, rainfall).*
Location	*State the location of the sampling or experiment, providing relevant parameters (e.g. latitude and longitude, elevation, water depth).*
Access & import/export	*Describe the efforts you have made to access habitats and to collect and import/export your samples in a responsible manner and in compliance with local, national and international laws, noting any permits that were obtained (give the name of the issuing authority, the date of issue, and any identifying information).*
Disturbance	*Describe any disturbance caused by the study and how it was minimized.*

Reporting for specific materials, systems and methods

We require information from authors about some types of materials, experimental systems and methods used in many studies. Here, indicate whether each material, system or method listed is relevant to your study. If you are not sure if a list item applies to your research, read the appropriate section before selecting a response.

Materials & experimental systems

n/a	Involved in the study	
	x	Antibodies
x		Eukaryotic cell lines
x		Palaeontology and archaeology
	x	Animals and other organisms
x		Human research participants
x		Clinical data
x		Dual use research of concern

Methods

n/a	Involved in the study	
x		ChIP-seq
	x	Flow cytometry
x		MRI-based neuroimaging

Antibodies

Antibodies used

Anti-CD3-eFluor710 eBioscience 46-0032
Anti-CD4-APC-eFluor® 780 eBioscience 47-0041
Anti-CD8a-APC-Cy7 Biolegend 100714
Anti-IL-22-APC Invitrogen 17-7222-82
Anti-Brdu-APC Biolegend 339807
Anti-CD44-APC Tonbo 20-0441-U100
Anti-TCRgd-BV510 Biolegend 118131
Anti-CD45Rb-PE eBioscience 12-0455-83
Anti-RORgt-PE eBioscience 12-6988-82
Anti-RORgt-APC eBioscience 17-6988-82
Anti-IFN-g-APC eBioscience 17-7311
Anti-IL-17A-PerCP Cy5.5 eBioscience 45-7177
Anti-IL-17A-PE eBioscience 12-7177
Anti-IL-5-APC Biolegend 504306
Anti-IL-13-Alexa Fluor488 Invitrogen 53-7133
Anti-IL-4-Percp-Cy5.5 Biolegend 504124
Anti-CD45.1-PE eBioscience 12-0453
Anti-CD45.1-BV450 BD 560520
Anti-CD45.2-Percp-Cy5.5 eBioscience 45-0454
Anti-Ki67-PE-Cy7 BD 561283
Anti-Annexin V-APC Invitrogen 17-8007
Anti-DCLK1 Abcam ab31704
Anti-EpCAM-APC BD 563478
Anti-PLZF-PE Biolegend 145803
Anti-IL-4 BIO X CELL BE0045
Anti-IL-13 InvivoGen mabg-mil13-5
Anti-Thy1.2 BioXcell BE0066
Anti-CD16/CD32 Invitrogen 14-0161-86
Anti-Vr4(A gift from Dr. Robert Tigelaar, Clone 17D1)
Anti-CD3-PE, BD, Cat#553063
Anti-KLRG1-Alexa488, Invitrogen, Cat#53-5893-82
Goat anti-Rabbit secondary Antibody, Alexa Fluor 488, Thermal Fisher, Cat# A11008

Validation

Anti-Vr4 antibody is a gift from Dr. Robert Tigelaar and validated in his publication (Christina L, et al,. Journal of Leukocyte Biology. 2004). All the rest antibodies used are from commercial sources and have been validated by the vendors. Validation data are available on the manufacturer's website.

Eukaryotic cell lines

Policy information about cell lines

Cell line source(s)

State the source of each cell line used.

Authentication

Describe the authentication procedures for each cell line used OR declare that none of the cell lines used were authenticated.

Mycoplasma contamination

Confirm that all cell lines tested negative for mycoplasma contamination OR describe the results of the testing for mycoplasma contamination OR declare that the cell lines were not tested for mycoplasma contamination.

Commonly misidentified lines
(See ICLAC register)

Name any commonly misidentified cell lines used in the study and provide a rationale for their use.

Palaeontology and Archaeology

Specimen provenance	*Provide provenance information for specimens and describe permits that were obtained for the work (including the name of the issuing authority, the date of issue, and any identifying information).*
Specimen deposition	*Indicate where the specimens have been deposited to permit free access by other researchers.*
Dating methods	*If new dates are provided, describe how they were obtained (e.g. collection, storage, sample pretreatment and measurement), where they were obtained (i.e. lab name), the calibration program and the protocol for quality assurance OR state that no new dates are provided.*

☐ Tick this box to confirm that the raw and calibrated dates are available in the paper or in Supplementary Information.

Ethics oversight	*Identify the organization(s) that approved or provided guidance on the study protocol, OR state that no ethical approval or guidance was required and explain why not.*

Note that full information on the approval of the study protocol must also be provided in the manuscript.

Animals and other organisms

Policy information about studies involving animals; ARRIVE guidelines recommended for reporting animal research

Laboratory animals	Mice used in this study were maintained in specific-pathogen-free (SPF) conditions at the University of Florida. All mouse studies were approved by the Animal Care and Use Committees of the University of Florida. Mice were littermate controlled and both male and female mice were used for experiments. Mice were used at 6 to 8-week-old age unless otherwise noted. Tfamfl/fl mice were kindly provided by Navdeep Chandel (Northwestern University). C57BL/6, TcrdCreER mice and Rorc-cre mice were purchased from Jackson Laboratory. Tfamfl/flTcrdCreER mice were generated by crossing Tfamfl/fl mice to TcrdCreER mice.
Wild animals	No wild animals were used in the study
Field-collected samples	No field collected samples were used in the study.
Ethics oversight	All mouse studies were approved by the Animal Care and Use Committees of the University of Florida.

Note that full information on the approval of the study protocol must also be provided in the manuscript.

Human research participants

Policy information about studies involving human research participants

Population characteristics	*Describe the covariate-relevant population characteristics of the human research participants (e.g. age, gender, genotypic information, past and current diagnosis and treatment categories). If you filled out the behavioural & social sciences study design questions and have nothing to add here, write "See above."*
Recruitment	*Describe how participants were recruited. Outline any potential self-selection bias or other biases that may be present and how these are likely to impact results.*
Ethics oversight	*Identify the organization(s) that approved the study protocol.*

Note that full information on the approval of the study protocol must also be provided in the manuscript.

Clinical data

Policy information about clinical studies
All manuscripts should comply with the ICMJEguidelines for publication of clinical research and a completedCONSORT checklist must be included with all submissions.

Clinical trial registration	*Provide the trial registration number from ClinicalTrials.gov or an equivalent agency.*
Study protocol	*Note where the full trial protocol can be accessed OR if not available, explain why.*
Data collection	*Describe the settings and locales of data collection, noting the time periods of recruitment and data collection.*
Outcomes	*Describe how you pre-defined primary and secondary outcome measures and how you assessed these measures.*

Dual use research of concern

Policy information about dual use research of concern

Hazards

Could the accidental, deliberate or reckless misuse of agents or technologies generated in the work, or the application of information presented in the manuscript, pose a threat to:

No	Yes	
☐	☐	Public health
☐	☐	National security
☐	☐	Crops and/or livestock
☐	☐	Ecosystems
☐	☐	Any other significant area

Experiments of concern

Does the work involve any of these experiments of concern:

No	Yes	
☐	☐	Demonstrate how to render a vaccine ineffective
☐	☐	Confer resistance to therapeutically useful antibiotics or antiviral agents
☐	☐	Enhance the virulence of a pathogen or render a nonpathogen virulent
☐	☐	Increase transmissibility of a pathogen
☐	☐	Alter the host range of a pathogen
☐	☐	Enable evasion of diagnostic/detection modalities
☐	☐	Enable the weaponization of a biological agent or toxin
☐	☐	Any other potentially harmful combination of experiments and agents

ChIP-seq

Data deposition

☐ Confirm that both raw and final processed data have been deposited in a public database such as GEO.

☐ Confirm that you have deposited or provided access to graph files (e.g. BED files) for the called peaks.

Data access links<br>*May remain private before publication.*	*For "Initial submission" or "Revised version" documents, provide reviewer access links. For your "Final submission" document, provide a link to the deposited data.*
Files in database submission	*Provide a list of all files available in the database submission.*
Genome browser session<br>(e.g. UCSC)	*Provide a link to an anonymized genome browser session for "Initial submission" and "Revised version" documents only, to enable peer review. Write "no longer applicable" for "Final submission" documents.*

Methodology

Replicates	*Describe the experimental replicates, specifying number, type and replicate agreement.*
Sequencing depth	*Describe the sequencing depth for each experiment, providing the total number of reads, uniquely mapped reads, length of reads and whether they were paired- or single-end.*
Antibodies	*Describe the antibodies used for the ChIP-seq experiments; as applicable, provide supplier name, catalog number, clone name, and lot number.*
Peak calling parameters	*Specify the command line program and parameters used for read mapping and peak calling, including the ChIP, control and index files used.*
Data quality	*Describe the methods used to ensure data quality in full detail, including how many peaks are at FDR 5% and above 5-fold enrichment.*
Software	*Describe the software used to collect and analyze the ChIP-seq data. For custom code that has been deposited into a community repository, provide accession details.*

Flow Cytometry

Plots

Confirm that:

- [x] The axis labels state the marker and fluorochrome used (e.g. CD4-FITC).
- [x] The axis scales are clearly visible. Include numbers along axes only for bottom left plot of group (a 'group' is an analysis of identical markers).
- [x] All plots are contour plots with outliers or pseudocolor plots.
- [x] A numerical value for number of cells or percentage (with statistics) is provided.

Methodology

Sample preparation	The isolation of lymphocytes from intestinal lamina propria was conducted by digesting minced lung tissues with collagenase VIII (Sigma) and DNase I (Sigma) at 37°C for 90 min. Lung lymphocyte isolation was conducted by digesting minced lung tissues with collagenase IV (Sigma) and DNase I (Sigma) at 37°C for 90 min. Fat lymphocyte isolation was conducted by digesting minced peritoneal adipose tissues with collagenase II (Sigma) with DNase I at 37°C for 90 min. Skin lymphocyte isolation was conducted by digesting minced ear skin tissues with collagenase IA (Sigma) with DNase I at 37°C for 60 min. After digestion, cells were further purified by 37.5% and 75% Percoll gradient for 20 min spin at 2,500 rpm. For flow cytometry analysis, the live and dead cells were stained by Live and Dead violet viability kit (Invitrogen) or Zombie Aqua fixable viability kit (BioLegend). anti-CD16/CD32 antibody (Thermo Fisher) was used to block the nonspecific binding followed by surface molecule staining at 4°C for 30 min. Cells were fixed and permeabilized with Foxp3 staining buffer Kit (eBioscience) for transcription factor staining. For cytokine staining, cells were stimulated with 50 ng/ml PMA and 500 ng/ml ionomycin for 4 hours and Brefeldin A (2 μg/ml) was added 2 hours before cells were harvested.
Instrument	BD FACSCantoII and LSRFortessa flow cytometer.
Software	BD FACSDIVA and FlowJo software (version 10) softwares were used for data collection and data analysis respectively.
Cell population abundance	Post-sort gdT17 cells were analyzed on BD LSRII and the purity of gdT17 cells was at least 95%.
Gating strategy	Lymphocytes were gated on FSC-A/SSC-A. Single cells were gated on FSC-A/FSC-H. Live cells were gated on Zombie Aqua negative population. gdT17 cells were gated as CD3+TCRd+RORgt+ or CD3+TCRd+CD44HighCD45Rb-.

- [x] Tick this box to confirm that a figure exemplifying the gating strategy is provided in the Supplementary Information.

Magnetic resonance imaging

Experimental design

Design type	*Indicate task or resting state; event-related or block design.*
Design specifications	*Specify the number of blocks, trials or experimental units per session and/or subject, and specify the length of each trial or block (if trials are blocked) and interval between trials.*
Behavioral performance measures	*State number and/or type of variables recorded (e.g. correct button press, response time) and what statistics were used to establish that the subjects were performing the task as expected (e.g. mean, range, and/or standard deviation across subjects).*

Acquisition

Imaging type(s)	*Specify: functional, structural, diffusion, perfusion.*
Field strength	*Specify in Tesla*
Sequence & imaging parameters	*Specify the pulse sequence type (gradient echo, spin echo, etc.), imaging type (EPI, spiral, etc.), field of view, matrix size, slice thickness, orientation and TE/TR/flip angle.*
Area of acquisition	*State whether a whole brain scan was used OR define the area of acquisition, describing how the region was determined.*

Diffusion MRI ☐ Used ☐ Not used

Preprocessing

Preprocessing software	*Provide detail on software version and revision number and on specific parameters (model/functions, brain extraction, segmentation, smoothing kernel size, etc.).*

Normalization	*If data were normalized/standardized, describe the approach(es): specify linear or non-linear and define image types used for transformation OR indicate that data were not normalized and explain rationale for lack of normalization.*
Normalization template	*Describe the template used for normalization/transformation, specifying subject space or group standardized space (e.g. original Talairach, MNI305, ICBM152) OR indicate that the data were not normalized.*
Noise and artifact removal	*Describe your procedure(s) for artifact and structured noise removal, specifying motion parameters, tissue signals and physiological signals (heart rate, respiration).*
Volume censoring	*Define your software and/or method and criteria for volume censoring, and state the extent of such censoring.*

Statistical modeling & inference

Model type and settings	*Specify type (mass univariate, multivariate, RSA, predictive, etc.) and describe essential details of the model at the first and second levels (e.g. fixed, random or mixed effects; drift or auto-correlation).*
Effect(s) tested	*Define precise effect in terms of the task or stimulus conditions instead of psychological concepts and indicate whether ANOVA or factorial designs were used.*

Specify type of analysis: ☐ Whole brain ☐ ROI-based ☐ Both

Statistic type for inference (See Eklund et al. 2016)	*Specify voxel-wise or cluster-wise and report all relevant parameters for cluster-wise methods.*
Correction	*Describe the type of correction and how it is obtained for multiple comparisons (e.g. FWE, FDR, permutation or Monte Carlo).*

Models & analysis

n/a	Involved in the study
☐	☐ Functional and/or effective connectivity
☐	☐ Graph analysis
☐	☐ Multivariate modeling or predictive analysis

Functional and/or effective connectivity	*Report the measures of dependence used and the model details (e.g. Pearson correlation, partial correlation, mutual information).*
Graph analysis	*Report the dependent variable and connectivity measure, specifying weighted graph or binarized graph, subject- or group-level, and the global and/or node summaries used (e.g. clustering coefficient, efficiency, etc.).*
Multivariate modeling and predictive analysis	*Specify independent variables, features extraction and dimension reduction, model, training and evaluation metrics.*
